# Synthesis of (Het)aryl 2-(2-hydroxyaryl)cyclopropyl Ketones

**DOI:** 10.3390/molecules25235748

**Published:** 2020-12-05

**Authors:** Alexander A. Fadeev, Alexey O. Chagarovskiy, Anton S. Makarov, Irina I. Levina, Olga A. Ivanova, Maxim G. Uchuskin, Igor V. Trushkov

**Affiliations:** 1 Department of Organic Chemistry, Faculty of Science, Charles University, Hlavova 8, 12800 Prague 2, Czech Republic; fadeeva@natur.cuni.cz; 2 Department of Chemistry, Perm State University, Bukireva 15, Perm 614990, Russia; antony.s.makarov@psu.ru; 3 N. D. Zelinsky Institute of Organic Chemistry, Leninsky pr. 47, Moscow 119991, Russia; alex.chagarovskiy@gmail.com (A.O.C.); iv@kinet.chem.msu.ru (O.A.I.); 4 Dmitry Rogachev National Medical Research Center of Pediatric Hematology, Oncology and Immunology, Samory Mashela 1, Moscow 117997, Russia; 5 N. M. Emanuel Institute of Biochemical Physcis Russian Academy of Sciences, Kosygina 4, Moscow 117198, Russia; iilevina@inbox.ru; 6 Department of Chemistry, M. V. Lomonosov Moscow State University, Leninskie gory 1-3, Moscow 119991, Russia

**Keywords:** 2-hydroxychalcones, Corey–Chaykovsky cyclopropanation, donor–acceptor cyclopropanes

## Abstract

A simple general method for the synthesis of 1-acyl-2-(*ortho*-hydroxyaryl)cyclopropanes, which belong to the donor–acceptor cyclopropane family, has been developed. This method, based on the Corey–Chaykovsky cyclopropanation of 2-hydroxychalcones, allows for the preparation of a large diversity of hydroxy-substituted cyclopropanes, which can serve as promising building blocks for the synthesis of various bioactive compounds.

## 1. Introduction

**Scheme 1:**
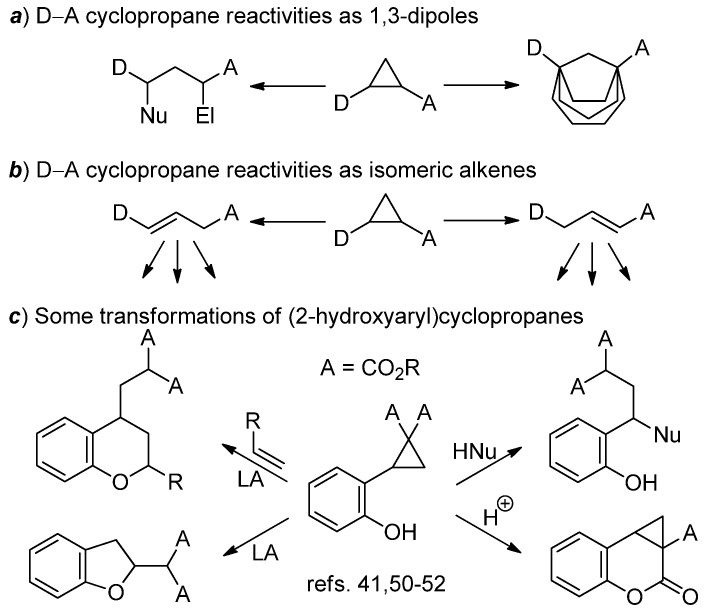
(**a**,**b**) Examples of reactivity of D–A cyclopropanes; (**c**) selected reactions of 2-hydroxyaryl-derived D–A cyclopropanes.

During last decades donor–acceptor (D–A) cyclopropanes [1,2,3,4,5,6] attracted a significant attention of organic chemists due to the excellent combination of their availability and high reactivity toward diverse classes of reaction partners: nucleophiles [4,7,8,9,10], electrophiles [11,12], radicals [13,14], dipolarophiles [15,16,17,18,19,20,21,22], dipoles [23,24,25], 1,3-dienes [26,27,28], etc. (Scheme 1a). In these reactions D–A cyclopropanes serve typically as synthetic equivalents of 1,3-dipoles providing approach to compounds, which are not easily accessible by other methods. In addition, D–A cyclopropanes can isomerize to alkenes conjugated to either electron-releasing [29,30,31] or electron-withdrawing groups [29,32,33] and exhibit reactivity of substituted styrenes (or their heterocyclic analogues) [31,34,35,36,37,38] or Michael acceptors [39,40,41,42,43,44,45], respectively (Scheme 1b). Moreover, D–A cyclopropanes can provide their acceptor [4,46,47,48] or donor [5,40,42,49] substituents for new bond formations. Among others, D–A cyclopropanes, bearing the hydroxy group at the *ortho*-position of a donor aromatic substituent, are especially interesting. Such cyclopropanes were shown to react as equivalents of *o*-quinone methide with alkenes affording chromane derivatives [50], undergo rearrangement to 2,3-dihydrobenzofurans [41], and participate in other transformations [50,51,52], including preparation of pharmacological agents (Scheme 1c) [53]. Furthermore, 2-hydroxyaryl-derived D–A cyclopropanes demonstrated bioactivity themselves, being selective antagonists of orexin 2 receptors [54] and showing antimicrobial and nematicidal activity [55].

Despite the promising reactivity and bioactivity of 2-hydroxyaryl-substituted cyclopropanes, their investigation is restricted by the absence of simple and efficient methods for their synthesis. In particular, the preparation of the corresponding cyclopropane-1,1-diesters requires protection of the phenolic oxygen [41,50], while the Corey–Chaykovsky cyclopropanation of easily available 2-hydroxychalcones produced a variety of products [56,57,58,59,60]. This presumably resulted from the highly activating effect of *ortho*-hydroxy group [50,60] on three-membered ring opening as well as the possible involvement of the nucleophilic phenoxy moiety into diverse transformations of 2-hydroxyaryl-derived D–A cyclopropanes. We report here the efficient procedure for the preparation of 1-acyl-2-(2-hydroxyaryl)cyclopropanes as potent bioactive compounds and promising building blocks for the synthesis of various acyclic, alicyclic and heterocyclic compounds.

## 2. Results and Discussion

**Scheme 2:**
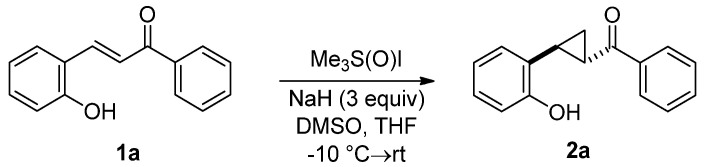
Corey–Chaykovsky cyclopropanation of 2-hydroxychalcone **1a**.

We investigated the reaction of trimethylsulfoxonium iodide with 2-hydroxychalcone **1a** as a model substrate. Varying base, solvent, temperature, ratio of the reacting compounds and order of their additiion, we found that cyclopropane **2a** can be obtained in 70% yield, when the solution of trimethylsulfoxonium iodide in DMSO/THF mixture was treated with 3 equivalents of sodium hydride followed by addition of enone **1a** to the formed reaction mixture at −10 °C and stirring for 3 h (Scheme 2).

The control of temperature and quenching procedure were found to be important for the good yield of the target product. Thus, the yield dropped significantly, if sodium hydride was added to the ice-cooled solution of starting compounds followed by removal of cooling bath. Moreover, compound **2a** was formed in trace amounts only, when all steps of process occurred at room temperature, the reaction being performed in DMF. Nevertheless, when addition of **1a** to the preformed ylide and stirring the reaction mixture were performed at 0 °C, product **2a** was obtained with acceptable yield. Quenching of the highly basic reaction mixture with ammonium chloride afforded **2a** in a good yield, while only trace amounts of cyclopropane **2a** was obtained when acetic acid was used for quenching.

**Scheme 3:**
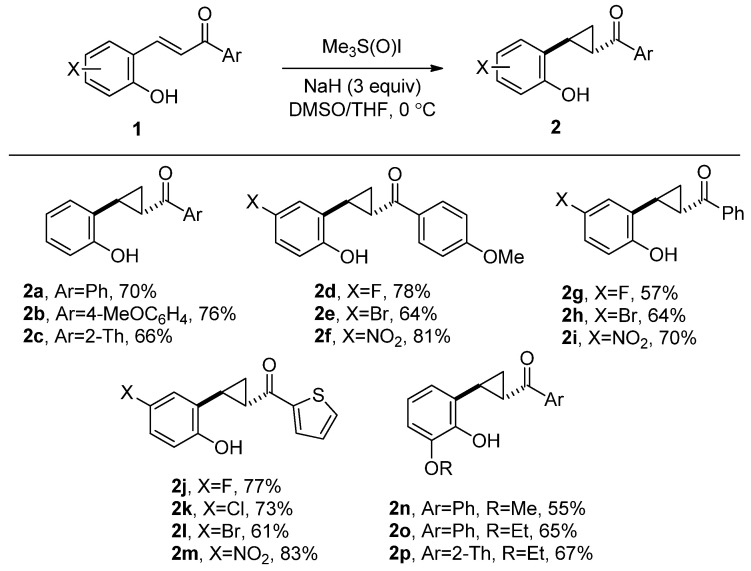
Synthesis of donor–acceptor (D–A) cyclopropanes **2**.

With the optimized reaction conditions in hand, we synthesized a series of 2-hydroxychalcone derivatives (see Experimental part) and studied the scope of Corey–Chaykovsky cyclopropanation of these substrates. We found that diverse substituents in the phenolic moiety (alkoxy, halogens, nitro group) were tolerant to the reaction conditions, and the corresponding cyclopropanes **2** were obtained in reasonable to high yields (Scheme 3). Electron-releasing substituents in the aroyl fragment have also no significant effect on the reaction yield. Oppositely, enones **1** with electron-depleted aroyl group, such as nicotinoyl or 4′-nitrobenzoyl, failed to produce the desired cyclopropane **2** due to side processes realization.

**Figure 1:**
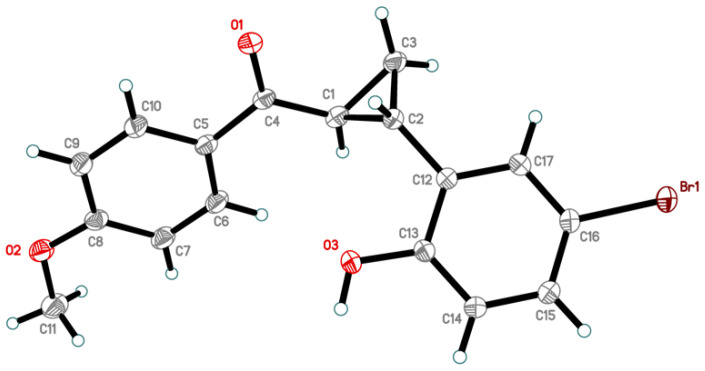
Single-crystal X-ray data for compound **2e** (CCDC 2042862).

Earlier it was pointed out that Corey–Chaykovsky cyclopropanation of the related enones proceeds typically with the retention of stereochemistry [61,62]. Indeed, all products were obtained as single diasteromers; *trans*-arrangement of donor and acceptor substituents in cyclopropane **2e** was unambiguously proved by single-crystal X-ray analysis (Figure 1). Crystal data for compound **2e** (C_17_H_15_BrO_3_, M = 347.20 g/mol): Orthorhombic, space group Pbca (no. 61), a = 9.4040(2) Å, b = 11.8308(3) Å, c = 27.0748(6) Å, α = 90°, β = 90°, γ = 90°, V = 3012.26(12) Å^3^, Z = 8, T = 100(2) K, μ(Mo Kα) = 2.736 mm^−1^, D_calc_ = 1.531 g/cm^3^, 92,781 reflections measured (2.637 < Θ < 37.788), 8080 unique (R(int) = 0.0454) which were used in all calculations. The final R1 was 0.03225 (I > 2σ(I)) and ωR2 was 0.0857 (all data).

Similar values of coupling constants for protons of three-membered rings support the conclusion that all synthesized cyclopropanes **2** have the same relative configuration of two stereocenters. It is noteworthy that C_(1)_–C_(2)_ bond length in **2e** (1.536 Å) is significantly larger than the bond length in the unsubstituted cyclopropane (1.510 Å, [63]). This bond elongation results from the significant polarization of the C_(1)_–C_(2)_ bond due to the cooperative effect of donor and acceptor substituents at the vicinal atoms of three-membered ring. On the other hand, this bond is significantly shorter than the corresponding bond in dimethyl 2-(5-bromo-2-hydroxyphenyl)cyclopropane-1,1-dicarboxylate (1.558 Å, [50]). This allows to suppose the lower reactivity of **2e** bearing 4-methoxybenzoyl group as an acceptor in comparison with the aforementioned diester.

## 3. Materials and Methods

### 3.1. General Information

The structures of synthesized compounds were elucidated with the aid of 1D (^1^H, ^13^C) and 2D (HSQC, NOESY) NMR spectroscopy. NMR spectra were acquired on Avance 500 and Avance 400 (Bruker, Billerica, MA, USA) spectrometers at room temperature; the chemical shifts *δ* were measured in ppm with respect to solvent (^1^H: CDCl_3_, *δ* = 7.26 ppm, DMSO-*d*_6_, *δ* = 2.50 ppm; ^13^C: CDCl_3_, *δ* = 77.16; DMSO-*d*_6_, *δ* = 39.52 ppm). Splitting patterns are designated as s, singlet; d, doublet; m, multiplet; dd, double doublet; br., broad. Coupling constants (*J*) are in Hertz. ^19^F NMR spectra were recorded at 471 MHz with fluorobenzene as an external reference (*δ* = −113.1 in DMSO-*d*_6_). Infrared spectra were recorded on an FTIR spectrometer ALPHA II (Bruker, Billerica, MA, USA) in KBr for solid substances and in nujol for oils. High resolution and accurate mass measurements were carried out using a micrOTOF-Q^TM^ ESI-TOF (Electrospray Ionization/Time of Flight, Bruker, Billerica, MA, USA) using ESI modes. X-Ray diffraction data were collected at 100 K on a Quest D8 diffractometer (Bruker, Billerica, MA, USA) equipped with a Photon-III area-detector (graphite monochromator, shutterless φ- and ω-scan technique) using Mo K_α_-radiation. Elemental analyses were performed with an EA-1108 CHNS elemental analyser instrument (Fisons, Ipswich, UK). Melting points (mp) are uncorrected and were measured on a 9100 capillary melting point apparatus (Electrothermal, Stone, UK). Analytical thin layer chromatography (TLC) was done on silica gel plates (silica gel 60, F_254_, supported on aluminium); visualization was done using a UV lamp (365 and 254 nm). Column chromatography was performed on silica gel 60 (230–400 mesh, Merck, Darmstadt, Germany). All reactions were performed using freshly distilled and dry solvents. Compound **1a** and other commercial reagents employed in the synthesis were analytical grade, obtained from Aldrich (St. Louis, MI, USA) or Alfa Aesar (Ward Hill, MO, USA). The NMR spectra for new compounds are available in the Supplementary Materials.

### 3.2. General Procedure for the Synthesis of 2-Hydroxychalcones ***1***

To a solution of aryl methyl ketone (4–10 mmol, 1 equiv) and (substituted) salicylaldehyde (4–10 mmol, 1 equiv), in EtOH (5–12 mL) was added 40% aq. NaOH (0.6–1.5 mL, 2.5 equiv) and the mixture was stirred at room temperature (or elevated temperature, if precipitation of intermediates occurred after addition of NaOH) for 12–48 h until the disappearance of starting material (monitored by thin layer chromatography). The reaction was poured into cold water (100–250 mL) and the mixture was neutralized with 2 M HCl to neutral or slightly acidic pH. The resulting precipitate was filtered, washed with water and air dried to afford the desired product. Crude product can be purified by recrystallization from appropriate solvent.

*(E)-3-(2-Hydroxyphenyl)-1-(4-methoxyphenyl)prop-2-en-1-one* (**1b**). Salicylaldehyde (1.22 g, 10.0 mmol), 4-methoxyacetophenone (1.50 g, 10.0 mmol), NaOH (1.00 g, 25.0 mmol), water (1.5 mL) 50 °C, 12 h. Yield 2.09 g (84%); light-yellow solid; mp = 147–148 °C (lit. 151–153 °C [64]; 148–149 °C [65]). Spectral data are consistent with the reported ones [64,65].

*(E)-3-(2-Hydroxyphenyl)-1-(2-thienyl)prop-2-en-1-one* (**1c**). Salicylaldehyde (611 mg, 5.0 mmol), 2-acetylthiophene (631 mg, 5.0 mmol), ethanol (6 mL), NaOH (500 mg, 12.5 mmol), water (0.75 mL), rt, 24 h. Yield 610 mg (53%); yellow solid; mp = 164–165 °C (lit. 165–168 °C [66], 158–159 °C [65]). Spectral data are consistent with the reported ones [65,66].

*(E)-3-(5-Fluoro-2-hydroxyphenyl)-1-(4-methoxyphenyl)prop-2-en-1-one* (**1d**). 5-Fluorosalicylaldehyde (561 mg, 4.0 mmol), 4-methoxyacetophenone (600 mg, 4.0 mmol), ethanol (5 mL), NaOH (400 mg, 10.0 mmol), water (0.6 mL), rt, 30 h. Yield 825 mg (76%); beige solid; mp = 161 °C (dec.). ^1^H-NMR (DMSO-*d*_6_, 500 MHz) *δ* = 3.86 (s, 3H, CH_3_O), 6.92–6.95 (m, 1H, Ar), 7.07 (d, ^3^*J =* 8.9 Hz, 2H, Ar), 7.08–7.13 (m, 1H, Ar), 7.80–7.82 (m, 1H, Ar), 7.92 (d, ^3^*J =* 15.7 Hz, 1H, HC=), 8.01 (d, ^3^*J =* 15.7 Hz, 1H, HC=), 8.14 (d, ^3^*J =* 8.9 Hz, 2H, Ar), 10.23 (br.s, 1H, OH). ^13^C NMR (DMSO-*d*_6_, 126 MHz) *δ* = 55.5 (CH_3_), 113.5 (d, ^2^*J_C–F_ =* 24 Hz, CH), 114.0 (2 × CH), 117.3 (d, ^3^*J_C–F_ =* 8 Hz, CH), 118.3 (d, ^2^*J_C–F_ =* 24 Hz, CH), 122.0 (CH), 122.5 (d, ^3^*J_C–F_ =* 8 Hz, C), 130.6 (CH), 130.9 (2 × CH), 137.2 (CH), 153.4 (C), 155.6 (d, ^1^*J_C–F_ =* 233 Hz, C), 163.2 (C), 187.5 (CO). ^19^F NMR (DMSO-*d*_6_, 471 MHz) *δ* = −125.0. IR (cm^−1^) 3280, 1650, 1600, 1585, 1560, 1515, 1420, 1370, 1320, 1265, 1245, 1175, 1025, 975, 835. HRMS ESI-TOF: *m*/*z* 273.0927 [M + H]^+^ (273.0921 calcd for C_16_H_14_FO_3_^+^). Anal. calcd. for C_16_H_13_FO_3_: C, 70.58; H, 4.81. Found: C, 70.56; H, 4.64.

*(E)-3-(5-Bromo-2-hydroxyphenyl)-1-(4-methoxyphenyl)prop-2-en-1-one* (**1e**). 5-Bromosalicylaldehyde (1.61 g, 8.0 mmol), 4-methoxyacetophenone (1.20 g, 8.0 mmol), ethanol (10 mL), NaOH (800 mg, 20.0 mmol), water (1.2 mL), rt, 24 h. Yield 2.24 mg (84%); bright yellow solid; mp = 174–175 °C (dec.) (lit. 177.0–177.3 °C [67]). Spectral data are consistent with the reported ones [67].

*(E)-3-(2-Hydroxy-5-nitrophenyl)-1-(4-methoxyphenyl)prop-2-en-1-one* (**1f**). 5-Nitrosalicylaldehyde (668 mg, 4.0 mmol), 4-methoxyacetophenone (600 mg, 4.0 mmol), ethanol (7.5 mL), NaOH (400 mg, 10.0 mmol), water (0.6 mL), 50 °C, 12 h. Yield 822 mg (69%); brick-red solid; mp = 215–216 °C (dec.). ^1^H NMR (DMSO-*d*_6_, 500 MHz) *δ* = 3.86 (s, 3H, CH_3_O), 6.55–6.57 (m, 1H, Ar), 7.07 (d, ^3^*J =* 8.9 Hz, 2H, Ar), 7.91 (d, ^3^*J =* 15.6 Hz, 1H, HC=), 7.91–7.94 (m, 1H, Ar), 8.11 (d, ^3^*J =* 8.9 Hz, 2H, Ar), 8.19 (d, ^3^*J =* 15.6 Hz, 1H, HC=), 8.48–8.51 (m, 3H, Ar). ^13^C NMR (DMSO-*d*_6_, 126 MHz) *δ* = 55.5 (CH_3_), 113.9 (2 × CH), 119.8 (CH), 120.1 (CH), 122.1 (CH), 127.1 (CH), 127.3 (CH), 130.6 (2 × CH), 131.1 (C), 133.4 (C), 140.4 (CH), 162.9 (C), 163.9 (C), 173.1 (C), 188.0 (CO). IR (cm^−1^) 3490, 3080, 1650, 1605, 1500, 1340, 1305, 1265, 1255, 1220, 1170, 1160, 1025, 835, 745. HRMS ESI-TOF: *m*/*z* 300.0871 [M + H]^+^ (300.0866 calcd for C_16_H_14_NO_5_^+^).

*(E)-3-(5-Fluoro-2-hydroxyphenyl)-1-phenylprop-2-en-1-one* (**1g**). 5-Fluorosalicylaldehyde (700 mg, 5.0 mmol), acetophenone (600 mg, 5.0 mmol), ethanol (6 mL), NaOH (500 mg, 12.5 mmol), water (0.75 mL), rt, 30 h. Yield 913 mg (75%); bright yellow solid; mp = 163 °C (dec). ^1^H NMR (DMSO-*d*_6_, 500 MHz) *δ* = 6.93–6.96 (m, 1H, Ar), 7.11–7.15 (m, 1H, Ar), 7.55–7.58 (m, 2H, Ar), 7.65–7.67 (m, 1H, Ar), 7.81–7.83 (m, 1H, Ar), 7.92 (d, ^3^*J =* 15.7 Hz, 1H, HC=), 8.03 (d, ^3^*J =* 15.7 Hz, 1H, HC=), 8.14–8.15 (m, 2H, Ar), 10.26 (br.s, 1H, OH). ^13^C NMR (DMSO-*d*_6_, 126 MHz) *δ* = 113.6 (d, ^2^*J_C–F_ =* 24 Hz, CH), 117.3 (d, ^3^*J_C–F_ =* 8 Hz, CH), 118.6 (d, ^2^*J_C–F_ =* 24 Hz, CH), 122.0 (CH), 122.3 (d, ^3^*J_C–F_ =* 8 Hz, C), 128.5 (2 × CH), 128.8 (2 × CH), 133.1 (CH), 137.7 (C), 138.1 (CH), 153.6 (C), 155.5 (d, ^1^*J_C–F_ =* 234 Hz, C), 189.3 (CO). ^19^F NMR (DMSO-*d*_6_, 471 MHz) *δ* = −125.0. IR (cm^−1^) 3365, 1655, 1600, 1585, 1570, 1500, 1450, 1350, 1280, 1245, 1210, 1180, 1155, 1020, 990, 850, 820, 720. HRMS ESI-TOF: *m*/*z* 243.0823 [M + H]^+^ (243.0816 calcd for C_15_H_12_FO_2_^+^).

*(E)-3-(5-Bromo-2-hydroxyphenyl)-1-phenylprop-2-en-1-one* (**1h**). 5-Bromosalicylaldehyde (1.61 g, 8.0 mmol), acetophenone (961 mg, 8.0 mmol), ethanol (10 mL), NaOH (800 mg, 20.0 mmol), water (1.2 mL), rt, 24 h. Yield 1.95 g (80%); bright yellow solid; mp = 162–163 °C (lit. 163.7–164.2 °C [67]). Spectral data are consistent with the reported ones [67,68].

*(E)-3-(2-Hydroxy-5-nitrophenyl)-1-phenylprop-2-en-1-one* (**1i**). 5-Nitrosalicylaldehyde (1.67 g, 10.0 mmol), acetophenone (1.2 g, 10.0 mmol), 20% aq. NaOH (5.5 mL, 27.5 mmol), EtOH (7 mL), 50 °C, 24 h. Yield 99 mg (70%); pale yellow solid; mp = 217–218 °C (ethyl acetate). Spectral data are consistent with the reported ones [68].

*(E)-3-(5-Fluoro-2-hydroxyphenyl)-1-(2-thienyl)prop-2-en-1-one* (**1j**). 5-Fluorosalicyl aldehyde (701 mg, 5.0 mmol), 2-acetylthiophene (631 mg, 5.0 mmol), ethanol (6 mL), NaOH (500 mg, 12.5 mmol), water (0.75 mL), rt, 24 h. Yield 829 mg (67%); bright yellow solid; mp = 158 °C (dec.). ^1^H NMR (DMSO-*d*_6_, 500 MHz) *δ* = 6.93–6.96 (m, 1H, Ar), 7.10–7.14 (m, 1H, Ar), 7.29–7.31 (m, 1H, Ar), 7.80–7.82 (m, 1H, Ar), 7.86 (d, ^3^*J =* 15.7 Hz, 1H, HC=), 8.00–8.04 (m, 2H, Ar + HC=), 8.29–8.30 (m, 1H, Ar), 10.19 (br.s, 1H, OH). ^13^C NMR (DMSO-*d*_6_, 126 MHz) *δ* 113.5 (d, ^2^*J_C–F_ =* 24 Hz, CH), 117.4 (d, ^3^*J_C–F_ =* 8 Hz, CH), 118.7 (d, ^2^*J_C–F_ =* 24 Hz, CH), 121.8 (CH), 122.2 (d, ^3^*J_C–F_ =* 8 Hz, C), 128.9 (CH), 133.5 (CH), 135.4 (CH), 137.2 (CH), 145.7 (C), 153.7 (C), 155.6 (d, ^1^*J_C–F_ =* 234 Hz, C), 181.7 (CO). ^19^F NMR (DMSO-*d*_6_, 471 MHz) *δ* = −124.9. IR (cm^−1^) 3230, 3115, 1635, 1570, 1505, 1440, 1415, 1355, 1280, 1245, 1180, 1150, 990, 840, 720. HRMS ESI-TOF: *m*/*z* 249.0383 [M + H]^+^ (249.0380 calcd for C_13_H_10_FO_2_S^+^).

*(E)-3-(5-Chloro-2-hydroxyphenyl)-1-(2-thienyl)prop-2-en-1-one* (**1k**). 5-Chlorosalicylaldehyde (626 mg, 4.0 mmol), 2-acetylthiophene (505 mg, 4.0 mmol), ethanol (5 mL), NaOH (400 mg, 10.0 mmol), water (0.6 mL), rt, 24 h. Yield 652 mg (62%); dark yellow solid; mp = 159–160 °C (dec.) (lit. 185–187 °C (AcOH) [69]). ^1^H NMR (DMSO-*d*_6_, 500 MHz) *δ* = 6.96 (d, ^3^*J =* 8.5 Hz, 1H, Ar), 7.29 (dd, ^3^*J =* 8.5 Hz, ^4^*J =* 2.6 Hz, 1H, Ar), 7.31 (dd, ^3^*J =* 5.0 Hz, ^3^*J =* 3.6 Hz, 1H, Th), 7.88 (d, ^3^*J =* 15.7 Hz, 1H, HC=), 7.98 (d, ^3^*J =* 15.7 Hz, 1H, HC=), 8.02 (d, ^4^*J =* 2.6 Hz, 1H, Ar), 8.04 (dd, ^3^*J =* 5.0 Hz, ^4^*J =* 0.8 Hz, 1H, Th), 8.33 (dd, ^3^*J =* 3.6 Hz, ^4^*J =* 0.8 Hz, 1H, Th), 10.59 (br.s, 1H, OH). ^13^C NMR (DMSO-*d*_6_, 126 MHz) *δ* *=* 117.9 (CH), 121.8 (CH), 122.9 (C), 123.3 (C), 127.3 (CH), 128.8 (CH), 131.4 (CH), 133.5 (CH), 135.3 (CH), 136.7 (CH), 145.6 (C), 155.9 (C), 181.7 (CO). IR (cm^−1^) 3235, 1640, 1565, 1515, 1490, 1415, 1340, 1285, 1235, 1170, 1115, 1065, 985, 845, 730. HRMS ESI-TOF: *m*/*z* 265.0083 [M + H]^+^ (265.0085 calcd for C_13_H_10_ClO_2_S^+^). Anal. calcd. for C_13_H_9_ClO_2_S: C, 58.98; H, 3.43. Found: C, 58.92; H, 3.35.

*(E)-3-(5-Bromo-2-hydroxyphenyl)-1-(2-thienyl)prop-2-en-1-one* (**1l**). 5-Bromosalicylaldehyde (1.61 g, 8.0 mmol), 2-acetylthiophene (1.01 g, 8.0 mmol), NaOH (800 mg, 20.0 mmol), ethanol (10 mL), water (1.2 mL), 50 °C, 12 h. Yield 2.21 g (89%); yellow solid; mp = 153–154 °C (dec.). ^1^H NMR (DMSO-*d*_6_, 500 MHz) *δ* = 6.91 (dd, ^3^*J =* 8.7 Hz, ^4^*J =* 1.4 Hz, 1H, Ar), 7.32 (ddd, ^3^*J =* 3.8 Hz, ^3^*J =* 5.0 Hz, ^4^*J =* 1.7 Hz, 1H, Th), 7.40 (dd, ^3^*J =* 5.0 Hz, ^4^*J =* 1.7 Hz, 1H, Th), 7.88 (dd, ^3^*J =* 15.7 Hz, ^4^*J =* 1.4 Hz, 1H, HC=), 7.99 (d, ^3^*J =* 15.7 Hz, 1H, HC=), 8.03–8.04 (m, 1H, Ar), 8.13–8.14 (m, 1H, Ar), 8.33–8.34 (m, 1H, Ar), 10.61 (br.s, 1H, OH). ^13^C NMR (DMSO-*d*_6_, 126 MHz) *δ =* 110.9 (C), 118.4 (CH), 121.8 (CH), 123.5 (C), 128.9 (CH), 130.2 (CH), 133.7 (CH), 134.3 (CH), 135.4 (CH), 136.6 (CH), 145.7 (C), 156.4 (C), 181.7 (CO). IR (cm^−1^) 3250, 3100, 1640, 1580, 1470, 1410, 1355, 1310, 1265, 1240, 1210, 1170, 1065, 995, 780, 720. HRMS ESI-TOF: *m*/*z* 332.9390 [M + Na]^+^ (332.9378 calcd for C_13_H_9_^81^BrO_2_SNa^+^). Anal. calcd. for C_13_H_9_BrO_2_S: C, 50.50; H, 2.93. Found: C, 50.18; H, 2.70.

*(E)-3-(2-Hydroxy-5-nitrophenyl)-1-(2-thienyl)prop-2-en-1-one* (**1m**). 5-Nitrosalicyl aldehyde (668 mg, 4.0 mmol), 2-acetylthiophene (505 mg, 4.0 mmol), ethanol (7.5 mL), NaOH (400 mg, 10.0 mmol), water (0.6 mL), 50 °C, 12 h. Yield 717 mg (65%); pale orange solid; mp = 205 °C (dec.). ^1^H NMR (DMSO-*d*_6_, 500 MHz) *δ* = 7.10 (d, ^3^*J =* 9.0 Hz, 1H, Ar), 7.32 (dd, ^3^*J =* 3.8 Hz, ^3^*J =* 4.9 Hz, 1H, Th), 7.97 (d, ^3^*J =* 15.7 Hz, 1H, HC=), 8.03 (d, ^3^*J =* 15.7 Hz, 1H, HC=), 8.07 (d, ^3^*J =* 4.9 Hz, 1H, Th), 8.16 (dd, ^3^*J =* 9.0 Hz, ^4^*J =* 2.8 Hz, 1H, Ar), 8.38 (d, ^3^*J =* 3.8 Hz, 1H, Th), 8.81 (d, ^4^*J =* 2.8 Hz, 1H, Ar), 11.89 (br.s, 1H, OH). ^13^C NMR (DMSO-*d*_6_, 126 MHz) *δ =* 116.6 (CH), 121.9 (C), 123.4 (CH), 124.4 (CH), 127.2 (CH), 128.9 (CH), 134.0 (CH), 135.7 (CH), 136.0 (CH), 140.0 (C), 145.4 (C), 162.7 (C), 181.6 (CO). IR (cm^−1^) 3220, 3110, 1640, 1615, 1565, 1520, 1490, 1410, 1355, 1340, 1305, 1290, 1100, 980, 845, 740. HRMS ESI-TOF: *m*/*z* 276.0326 [M + H]^+^ (276.0325 calcd for C_13_H_10_NO_4_S^+^). Anal. calcd. for C_13_H_9_NO_4_S: C, 56.72; H, 3.30; N, 5.09. Found: C, 56.51; H, 3.25; N, 4.94.

*(E)-3-(2-Hydroxy-3-methoxyphenyl)-1-phenylprop-2-en-1-one* (**1n**). 3-Methoxysalicyl aldehyde (1.520 g, 10.0 mmol), acetophenone (1.20 g, 10.0 mmol), ethanol (12.0 mL), NaOH (1.00 g, 25.0 mmol), water (1.5 mL), 50 °C, 12 h. Yield 1.67 g (66%); yellow solid; mp = 110–112 °C (lit. 109–111 °C [70]; 115 °C [71]). Spectral data are consistent with the reported ones [68,70,71].

*(E)-3-(3-Ethoxy-2-hydroxyphenyl)-1-phenylprop-2-en-1-one* (**1o**). 3-Ethoxysalicylaldehyde (1.66 g, 10.0 mmol), acetophenone (1.20 g, 10.0 mmol), ethanol (12.0 mL), NaOH (1.00 g, 25.0 mmol), water (1.5 mL), rt, 48 h. Yield 1.72 g (64%); dark yellow solid; mp = 172–173 °C (dec.). ^1^H NMR (DMSO-*d*_6_, 500 MHz) *δ* = 1.36 (t, ^3^*J =* 7.0 Hz, 3H, CH_3_), 4.06 (q, ^3^*J =* 7.0 Hz, 2H, CH_2_), 6.81–6.84 (m, 1H, Ar), 7.00–7.02 (m, 1H, Ar), 7.47–7.49 (m, 1H, Ar), 7.55–7.58 (m, 2H, Ar), 7.63–7.66 (m, 1H, Ar), 7.85 (d, ^3^*J =* 15.7 Hz, 1H, HC=), 8.10–8.12 (m, 2H, Ar), 8.13 (d, ^3^*J =* 15.7 Hz, 1H, HC=), 9.26 (br.s, 1H, OH). ^13^C NMR (DMSO-*d*_6_, 126 MHz) *δ* = 14.6 (CH_3_), 64.3 (CH_2_), 114.8 (CH), 119.1 (CH), 119.7 (CH), 121.8 (CH), 121.6 (C), 128.3 (2 × CH), 128.7 (2 × CH), 132.8 (CH), 137.8 (C), 139.3 (CH), 146.8 (C), 147.0 (C), 189.5 (CO). IR (cm^−1^) 3365, 2980, 1655, 1585, 1470, 1310, 1265, 1235, 1210, 1170, 1065, 1000, 850, 765. HRMS ESI-TOF: *m*/*z* 291.0991 [M + Na]^+^ (291.0992 calcd for C_17_H_16_O_3_Na^+^).

*(E)-3-(3-Ethoxy-2-hydroxyphenyl)-1-(2-thienyl)prop-2-en-1-one* (**1p**). 3-Ethoxysalicylaldehyde (1.66 g, 10.0 mmol), 2-acetylthiophene (1.26 g, 10.0 mmol), ethanol (12.0 mL), NaOH (1.00 g, 25.0 mmol), water (1.5 mL), rt, 48 h. Yield 1.86 mg (68%); brownish yellow solid; mp = 168–169 °C (dec.). ^1^H NMR (DMSO-*d*_6_, 500 MHz) *δ* = 1.36 (t, ^3^*J =* 7.0 Hz, 3H, CH_3_), 4.06 (q, ^3^*J =* 7.0 Hz, 2H, CH_2_), 6.80–6.84 (m, 1H, Ar), 7.00–7.01 (m, 1H, Ar), 7.29–7.31 (m, 1H, Ar), 7.48–7.50 (m, 1H, Ar), 7.78 (d, ^3^*J =* 15.7 Hz, 1H, HC=), 8.02–8.03 (m, 1H, Ar), 8.13 (d, ^3^*J =* 15.7 Hz, 1H, HC=), 8.23–8.24 (m, 1H, Ar). ^13^C NMR (DMSO-*d*_6_, 126 MHz) *δ* = 14.6 (CH_3_), 64.3 (CH_2_), 114.8 (CH), 119.1 (CH), 119.7 (CH), 120.8 (CH), 121.6 (C), 128.9 (CH), 133.1 (CH), 135.1 (CH), 138.4 (C), 145.7 (C), 147.2 (C), 181.8 (CO). IR (cm^−1^) 3310, 2980, 1640, 1580, 1470, 1410, 1355, 1310, 1265, 1240, 1210, 1170, 1065, 995, 780, 720. HRMS ESI-TOF: *m*/*z* 275.0738 [M + H]^+^ (275.0736 calcd for C_15_H_15_O_3_S^+^). Anal. calcd. for C_15_H_14_O_3_S: C, 65.67; H, 5.14. Found: C, 65.54; H, 4.97.

### 3.3. General Procedure for the Synthesis of Donor–Acceptor Cyclopropanes ***2***

Trimethylsulfoxonium iodide (242 mg, 1.1 mmol) was dissolved in ice-cooled mixture of THF/DMSO (1:1, 10 mL) and 60% suspension of sodium hydride in mineral oil (120 mg, 3 mmol) was added. The mixture was stirred under argon at the same temperature until the evolution of gas stopped (30–40 min). Then 1 mmol of the corresponding unsaturated ketone was added in 2–3 portions. The reaction mixture was stirred at 0 °C for 1–2 h, quenched with cold aqueous NH_4_Cl solution and extracted with EtOAc (3 × 10 mL). The combined organic layer was washed with water (5 × 10 mL), brine (1 × 10 mL) and dried over Na_2_SO_4_. After concentration in vacuo, the residue was purified by column chromatography on a silica gel to afford the desired product.

*[(1RS,2RS)-2-(2-Hydroxyphenyl)cyclopropyl](phenyl)methanone* (**2a**). 2-Hydroxychalcone **1a** (224 mg, 1.00 mmol). Yield 167 mg (70%); beige solid; mp = 94–95 °C (lit. 86–87 °C (benzene) [56]). *R*_f_ 0.50 (petroleum ether:ethyl acetate 3:1). ^1^H NMR (CDCl_3_, 500 MHz) *δ* = 1.61–1.64 (m, 1H, CH_2_), 1.89–1.93 (m, 1H, CH_2_), 2.87–2.94 (m, 2H, CH+CH), 6.34 (s, 1H, OH), 6.90–6.94 (m, 2H, Ar), 7.10–7.11 (m, 1H, Ar), 7.16–7.19 (m, 1H, Ar), 7.44–7.47 (m, 2H, Ar), 7.55–7.58 (m, 1H, Ar), 8.03–8.05 (m, 2H, Ar). ^1^H NMR (DMSO-*d*_6_, 500 MHz) *δ* = 1.56 (ddd, ^3^*J =* 7.9 Hz, ^3^*J =* 6.9 Hz, ^2^*J =* 3.6 Hz, 1H, CH_2_), 1.67 (ddd, ^3^*J =* 8.9 Hz, ^3^*J =* 5.1 Hz, ^2^*J =* 3.6 Hz, 1H, CH_2_), 2.68 (ddd, ^3^*J =* 8.9 Hz, ^3^*J =* 6.9 Hz, ^3^*J =* 4.2 Hz, 1H, CH), 3.02 (ddd, ^3^*J =* 7.9 Hz, ^3^*J =* 5.1 Hz, ^3^*J =* 4.2 Hz, 1H, CH), 6.75 (dt, ^3^*J =* 7.5 Hz, ^4^*J =* 1.2 Hz, 1H, Ar), 6.80 (dd, ^3^*J =* 8.4 Hz, ^4^*J =* 1.2 Hz, 1H, Ar), 7.00–7.08 (m, 2H, Ar), 7.50–7.57 (m, 2H, Ar), 7.61–7.67 (m, 1H, Ar), 8.00–8.09 (m, 2H, Ar), 9.48 (br.s, 1H, OH). ^13^C NMR (CDCl_3_, 126 MHz) *δ* = 17.9 (CH_2_), 25.1 (CH), 27.3 (CH), 115.6 (CH), 120.5 (CH), 126.1 (C), 127.5 (CH), 128.2 (CH), 128.3 (2 × CH), 128.7 (2 × CH), 133.1 (CH), 137.7 (C), 155.4 (C), 200.2 (CO). IR (cm^−1^) 3365, 3040, 1645, 1595, 1455, 1365, 1265, 1230, 1040, 990, 835, 750, 720. HRMS ESI-TOF: *m*/*z* 239.1066 [M + H]^+^ (239.1067 calcd for C_16_H_15_O_2_^+^). Anal. calcd for C_16_H_14_O_2_: C, 80.65; H, 5.92. Found: C, 80.68; H, 5.90.

*[(1RS,2RS)-2-(2-Hydroxyphenyl)cyclopropyl](4-methoxyphenyl)methanone* (**2b**). 2-Hydroxy-4′-methoxychalcone **1b** (254 mg, 1.00 mmol). Yield 204 mg (76%); yellow solid; mp = 129–130 °C. *R*_f_ 0.47 (petroleum ether:ethyl acetate 3:1). ^1^H NMR (DMSO-*d*_6_, 500 MHz) *δ* = 1.51 (ddd, ^3^*J =* 7.7 Hz, ^3^*J =* 6.8 Hz, ^2^*J =* 3.7 Hz, 1H, CH_2_), 1.65 (ddd, ^3^*J =* 8.8 Hz, ^3^*J =* 5.1 Hz, ^2^*J =* 3.7 Hz, 1H, CH_2_), 2.70 (ddd, ^3^*J =* 8.8 Hz, ^3^*J =* 6.8 Hz, ^3^*J =* 4.1 Hz, 1H, CH), 2.94–2.99 (m, 1H, CH), 3.82 (s, 3H, CH_3_O), 6.73–6.78 (m, 1H, Ar), 6.83 (dd, ^3^*J =* 7.9 Hz, ^4^*J =* 1.2 Hz, 1H, Ar), 6.98–7.06 (m, 4H, Ar), 8.00–8.04 (m, 2H, Ar), 9.48 (br.s, 1H, OH). ^13^C NMR (DMSO-*d*_6_, 126 MHz) *δ* = 17.3, 24.1, 26.8, 55.5, 113.9 (2C), 114.8, 119.0, 125.8, 126.5, 127.2, 130.2, 130.3 (2C), 156.0, 163.1, 196.6. IR (cm^−1^) 3305, 3290, 3080, 1625, 1605, 1465, 1425, 1360, 1265, 1210, 1115, 1035, 1010, 970, 865, 775, 735, 725. HRMS ESI-TOF: *m*/*z* 291.0996 [M + Na]^+^ (291.0992 calcd for C_17_H_16_NaO_3_^+^). Anal. calcd for C_17_H_16_O_3_: C, 76.10; H, 6.01. Found: C, 75.98; H, 6.12.

*[(1RS,2RS)-2-(2-Hydroxyphenyl)cyclopropyl](thiophen-2-yl)methanone* (**2c**). 3-(2-Hydroxyphenyl)-1- (thiophen-2-yl)prop-2-en-1-one **1c** (230 mg, 1.00 mmol). Yield 161 mg (66%); yellow solid; mp = 103–104 °C. *R*_f_ 0.47 (petroleum ether:ethyl acetate 3:1). ^1^H NMR (CDCl_3_, 500 MHz) *δ* = 1.59 (ddd, ^3^*J =* 8.0 Hz, ^3^*J =* 6.9 Hz, ^2^*J =* 3.9 Hz, 1H, CH_2_), 1.88 (ddd, ^3^*J =* 9.0 Hz, ^3^*J =* 5.1 Hz, ^2^*J =* 3.9 Hz, 1H, CH_2_), 2.77 (ddd, ^3^*J =* 8.0 Hz, ^3^*J =* 5.1 Hz, ^3^*J =* 4.1 Hz, 1H, CH), 2.87 (ddd, ^3^*J =* 9.0 Hz, ^3^*J =* 6.9 Hz, ^3^*J =* 4.1 Hz, 1H, CH), 6.37 (s, 1H, OH), 6.85–6.93 (m, 2H, Ar), 7.07 (dd, ^3^*J =* 7.8 Hz, ^4^*J =* 1.7 Hz, 1H, Ar), 7.10 (dd, ^3^*J =* 4.9 Hz, ^3^*J =* 3.8 Hz, 1H, Th), 7.12–7.18 (m, 1H, Ar), 7.61 (dd, ^3^*J =* 4.9 Hz, ^4^*J =* 1.1 Hz, 1H, Th), 7.83 (dd, ^3^*J =* 3.8 Hz, ^4^*J =* 1.1 Hz, 1H, Th). ^13^C NMR (CDCl_3_, 126 MHz) *δ* = 17.6, 24.6, 28.0, 115.7, 120.5, 125.9, 127.6, 128.2, 128.4, 132.4, 134.0, 144.6, 155.4, 192.2. IR (cm^−1^) 3305, 3290, 3080, 1625, 1605, 1465, 1425, 1360, 1265, 1210, 1115, 1035, 1010, 970, 865, 775, 735, 725. HRMS ESI-TOF: *m*/*z* 267.0450 [M + Na]^+^ (267.0450 calcd for C_14_H_12_NaO_2_S^+^). Anal. calcd for C_14_H_12_O_2_S: C, 68.83; H, 4.95. Found: C, 68.79; H, 4.87.

*[(1RS,2RS)-2-(5-Fluoro-2-hydroxyphenyl)cyclopropyl](4-methoxyphenyl)methanone* (**2d**). 5-Fluoro-2-hydroxy-4′-methoxychalcone **1d** (272 mg, 1.00 mmol). Yield 223 mg (78%); yellow solid; mp = 131–132 °C. *R*_f_ 0.47 (petroleum ether:ethyl acetate 3:1). ^1^H NMR (DMSO-*d*_6_, 500 MHz) *δ* = 1.51 (ddd, ^3^*J =* 8.0 Hz, ^3^*J =* 6.9 Hz, ^2^*J =* 3.7 Hz, 1H, CH_2_), 1.63 (ddd, ^3^*J =* 8.9 Hz, ^3^*J =* 5.0 Hz, ^2^*J =* 3.7 Hz, 1H, CH_2_), 2.66–2.75 (m, 1H, CH), 3.02–3.09 (m, 1H, CH), 3.83 (s, 3H, OCH_3_), 6.76–6.90 (m, 3H, Ar), 7.03 (d, ^3^*J =* 8.8 Hz, 2H, Ar), 8.04 (d, ^3^*J =* 8.8 Hz, 2H, Ar), 9.46 (s, 1H, OH); ^13^C NMR (DMSO-*d_6_*, 126 MHz) *δ* = 17.7, 23.5, 26.8, 55.5, 112.1 (d, ^2^*J_C–F_ =* 23 Hz), 113.1 (d, ^2^*J_C–F_ =* 23 Hz), 113.9 (2C), 115.5 (d, ^3^*J_C–F_ =* 8 Hz), 128.4 (d, ^3^*J_C–F_ =* 8 Hz), 130.1, 130.3 (2C), 152.1, 155.8 (d, ^1^*J_C–F_ =* 233 Hz), 163.1, 196.3. ^19^F NMR (DMSO-*d*_6_, 471 MHz) *δ* = −125.2. IR (cm^−1^) 3325, 3015, 1635, 1600, 1570, 1515, 1440, 1265, 1240, 1175, 1025, 845, 820, 755. HRMS ESI-TOF: *m*/*z* 287.1077 [M + H]^+^ (287.1078 calcd for C_17_H_16_FO_3_^+^). Anal. calcd for C_17_H_15_FO_3_: C, 71.32; H, 5.28. Found: C, 71.32; H, 5.19.

*[(1RS,2RS)-2-(2-5-Bromo-2-hydroxyphenyl)cyclopropyl](4-methoxyphenyl)methanone* (**2e**). 5-Bromo-2-hydroxy-4′-methoxychalcone **1e** (333 mg, 1.00 mmol). Yield 222 mg (64%); colorless solid; mp 143–144 °C. *R*_f_ 0.40 (petroleum ether:ethyl acetate 3:1). ^1^H NMR (CDCl_3_, 500 MHz): *δ* 1.50 (ddd, ^3^*J =* 8.2 Hz, ^3^*J =* 6.7 Hz, ^2^*J =* 3.9 Hz, 1H, CH_2_), 1.80 (ddd, ^3^*J =* 9.0 Hz, ^3^*J =* 5.2 Hz, ^2^*J =* 3.9 Hz, 1H, CH_2_), 2.71–2.81 (m, 2H, 2 × CH), 3.86 (s, 3H, OCH_3_), 6.18 (s, 1H, OH), 6.75 (d, ^3^*J =* 8.5 Hz, 1H, Ar), 6.90 (d, ^3^*J =* 8.9 Hz, 2H, Ar), 7.15 (d, ^4^*J =* 2.3 Hz, 1H, Ar), 7.23 (dd, ^3^*J =* 8.5 Hz, ^4^*J =* 2.3 Hz, 1H, Ar), 7.97 (d, ^3^*J =* 8.9 Hz, 2H, Ar). ^13^C NMR (CDCl_3_, 126 MHz): *δ* 17.3, 23.4, 26.7, 55.6, 112.6, 114.0 (2C), 117.5, 128.5, 130.3, 130.4, 130.7 (2C), 131.0, 154.6, 163.8, 197.7. IR (cm^−1^) 3220, 2930, 1620, 1600, 1565, 1425, 1410, 1265, 1235, 1165, 1030, 995, 850, 815, 750. HRMS ESI-TOF: *m*/*z* 347.0280/349.0258 [M + H]^+^ (347.0277/349.0258 calcd for C_17_H_16_^79^BrO_3_^+^/C_17_H_16_^81^BrO_3_^+^). Anal. calcd for C_17_H_15_BrO_3_: C, 58.81; H, 4.35. Found: C, 58.80; H, 4.29.

*[(1RS,2RS)-2-(2-Hydroxy-5-nitrophenyl)cyclopropyl](4-methoxyphenyl)methanone* (**2f**). 2-Hydroxy-5-nitro-4′-methoxychalcone **1f** (554 mg, 1.85 mmol). Yield 470 mg (81%); yellow solid; mp 183–184 °C. *R*_f_ 0.69 (petroleum ether:ethyl acetate 3:1). ^1^H NMR (DMSO-*d*_6_, 500 MHz) *δ* = 1.58–1.67 (m, 2H, CH_2_), 2.69 (ddd, ^3^*J =* 8.9 Hz, ^3^*J =* 6.9 Hz, ^3^*J =* 4.2 Hz, 1H, CH), 3.14 (ddd, ^3^*J =* 8.0 Hz, ^3^*J =* 5.2 Hz, ^3^*J =* 4.2 Hz, 1H, CH), 3.84 (s, 3H, OCH_3_), 6.97 (d, ^3^*J =* 8.9 Hz, 1H, Ar), 7.02–7.08 (m, 2H, Ar), 7.91 (d, ^4^*J =* 2.8 Hz, 1H, Ar), 8.01 (dd, ^3^*J =* 8.9 Hz, ^4^*J =* 2.8 Hz, 1H, Ar), 8.04–8.10 (m, 2H, Ar), 11.21 (s, 1H, OH). ^13^C NMR (DMSO-*d_6_*, 125 MHz): *δ* 17.2, 23.0, 26.5, 55.5, 113.9 (2C), 114.9, 122.1, 124.0, 128.0, 130.0, 130.4 (2C), 139.7, 162.6, 163.2, 196.2. IR (cm^−1^) 2925, 1635, 1595, 1565, 1530, 1495, 1335, 1285, 1235, 1175, 1090, 1010, 925, 830, 745. HRMS ESI-TOF: *m*/*z* 314.1022 [M + H]^+^ (314.1023 calcd for C_17_H_16_NO_5_^+^).

*[(1RS,2RS)-2-(5-Fluoro-2-hydroxyphenyl)cyclopropyl](phenyl)methanone* (**2g**). 5-Fluoro-2-hydroxychalcone **1g** (242 mg, 1.00 mmol). Yield 147 mg (57% yield); yellow solid; mp 95–96 °C. R*_f_* 0.81 (petroleum ether:ethyl acetate 3:1). ^1^H NMR (CDCl_3_, 500 MHz) *δ* = 1.51–1.57 (m, 1H, CH_2_), 1.87 (ddd, ^3^*J =* 8.2 Hz, ^3^*J =* 5.9 Hz, ^2^*J =* 4.0 Hz, 1H, CH_2_), 2.84–2.91 (m, 2H, CH + CH), 6.66–6.77 (m, 2H, Ar + OH), 6.77–6.84 (m, 2H, Ar), 7.36–7.45 (m, 2H, Ar), 7.48–7.57 (m, 1H, Ar), 7.93–8.04 (m, 2H, Ar). ^13^C NMR (CDCl_3_, 126 MHz) *δ* = 18.1, 25.0, 27.6, 113.4 (d, ^2^*J_C–F_ =* 23 Hz), 114.2 (d, ^2^*J_C–F_ =* 23 Hz), 116.5 (d, ^3^*J_C–F_ =* 9 Hz), 127.3 (d, ^3^*J_C–F_ =* 7 Hz), 128.3 (2C), 128.7 (2C), 133.3, 137.4, 151.4, 155.9 (d, ^1^*J_C–F_ =* 234 Hz), 200.4. ^19^F NMR (DMSO-*d*_6_, 471 MHz) *δ* = −125.3. IR (cm^−1^) 3355, 3085, 1640, 1510, 1440, 1365, 1270, 1230, 1180, 1015, 810, 770, 715. HRMS ESI-TOF: *m*/*z* 257.0979 [M + H]^+^ (257.0972 calcd for C_16_H_14_FO_2_^+^). Anal. calcd for C_16_H_13_FO_2_: C, 74.99; H, 5.11. Found: C, 74.98; H, 4.97.

*[(1RS,2RS)-2-(5-Bromo-2-hydroxyphenyl)cyclopropyl](phenyl)methanone* (**2h**). 5-Bromo-2-hydroxychalcone **1h** (272 mg, 0.90 mmol). Yield 182 mg (64% yield); colorless solid; mp 117–118 °C. R*_f_* 0.40 (petroleum ether:ethyl acetate 3:1). ^1^H NMR (CDCl_3_, 500 MHz) *δ* = 1.55 (ddd, ^3^*J =* 8.3 Hz, ^3^*J =* 6.8 Hz, ^2^*J =* 4.0 Hz, 1H, CH_2_), 1.80–1.86 (m, 1H, CH_2_), 2.79 (ddd, ^3^*J =* 9.0 Hz, ^3^*J =* 6.8 Hz, ^3^*J =* 4.3 Hz 1H, CH), 2.81–2.89 (m, 1H, CH), 5.96 (s, 1H, OH), 6.74 (d, ^3^*J =* 8.5 Hz, 1H, Ar), 7.14–7.16 (m, 1H, Ar), 7.19–7.25 (m, 1H, Ar), 7.40–7.48 (m, 2H, Ar), 7.52–7.60 (m, 1H, Ar), 7.93–8.02 (m, 2H, Ar). ^13^C NMR (CDCl_3_, 126 MHz) *δ* = 17.8, 24.0, 27.1, 112.6, 117.4, 128.3 (2C), 128.4, 128.8 (2C), 130.3, 131.0, 133.4, 137.5, 154.5, 199.4. IR (cm^−1^) 3210, 2980, 1625, 1590, 1445, 1405, 1390, 1240, 1165, 1030, 1000, 790, 730. HRMS ESI-TOF: *m*/*z* 317.0166/319.0147 [M + H]^+^ (317.0172/319.0152 calcd for C_16_H_14_^79^BrO_2_^+^/C_16_H_14_^81^BrO_2_^+^).

*[(1RS,2RS)-2-(2-Hydroxy-5-nitrophenyl)cyclopropyl](phenyl)methanone* (**2i**). 2-Hydroxy-5-nitrochalcone **1i** (135 mg, 0.50 mmol). Yield 99 mg (70%); pale yellow solid; ^1^H NMR (DMSO-*d*_6_, 400 MHz) *δ* = 1.63–1.74 (m, 2H, CH_2_), 2.68–2.76 (m, 1H, CH), 3.12–3.22 (m, 1H, CH), 6.91–7.03 (m, 1H, Ar), 7.47–7.58 (m, 2H, Ar), 7.61–7.70 (m, 1H, Ar), 7.88–7.96 (m, 1H, Ar), 7.99–8.14 (m, 3H, Ar), 11.23 (br. s, 1H, OH). ^13^C NMR (DMSO-*d*_6_, 100 MHz) *δ* = 17.5, 23.6, 27.0, 114.9, 122.2, 124.0, 127.8, 128.1 (2C), 128.7 (2C), 133.2, 137.0, 139.7, 162.6, 198.0. HRMS (ESI/TOF): *m*/*z* 284.0920 [M + H]^+^ (284.0917 calcd. for C_16_H_14_NO_4_^+^).

*[(1RS,2RS)-2-(5-Fluoro-2-hydroxyphenyl)cyclopropyl](thiophen-2-yl)methanone* (**2j**). 3-(5-Fluoro-2-hydroxyphenyl)-1-(thiophen-2-yl)prop-2-en-1-one **1j** (496 mg, 2.00 mmol). Yield 405 mg (77% yield); yellow solid; mp 107–108 °C. R*_f_* 0.53 (petroleum ether:ethyl acetate 3:1). ^1^H NMR (CDCl_3_, 500 MHz) *δ* = 1.51 (ddd, ^3^*J =* 8.2 Hz, ^3^*J =* 6.8 Hz, ^2^*J =* 4.1 Hz, 1H, CH_2_), 1.86 (ddd, ^3^*J =* 9.0 Hz, ^3^*J =* 5.1 Hz, ^2^*J =* 4.1 Hz, 1H, CH_2_), 2.73 (ddd, ^3^*J =* 8.2 Hz, ^3^*J =* 5.1 Hz, ^3^*J =* 4.2 Hz, 1H, CH), 2.81 (ddd, ^3^*J =* 9.0 Hz, ^3^*J =* 6.8 Hz, ^3^*J =* 4.2 Hz, 1H, CH), 5.72 (br. s, 1H, OH), 6.70–6.87 (m, 3H, Ar), 7.14 (dd, ^3^*J =* 4.9 Hz, ^3^*J =* 3.8 Hz, 1H, Th), 7.65 (dd, ^3^*J =* 4.9 Hz, ^4^*J =* 0.9 Hz, 1H, Th), 7.84 (dd, ^3^*J =* 3.8 Hz, ^4^*J =* 0.9 Hz, 1H, Th). ^13^C NMR (CDCl_3_, 125 MHz) *δ* = 17.6, 23.8, 28.0, 113.8 (d, ^2^*J_C–F_ =* 24 Hz), 114.4 (d, ^2^*J_C–F_ =* 24 Hz), 116.5 (d, ^3^*J_C–F_ =* 8 Hz), 127.4 (d, ^3^*J_C–F_ =* 7 Hz), 128.5, 132.4, 134.2, 144.5, 151.2, 157.0 (d, ^1^*J_C–F_ =* 239 Hz), 191.4. ^19^F NMR (DMSO-*d*_6_, 471 MHz) *δ* = −125.3. IR (cm^−1^) 3370, 3095, 1620, 1555, 1495, 1410, 1370, 1345, 1270, 1220, 1100, 1050, 905, 855, 755, 715. Anal. calcd for C_14_H_11_FO_2_S: C, 64.11; H, 4.23. Found: C, 64.22; H, 4.17.

*[(1RS,2RS)-2-(5-Chloro-2-hydroxyphenyl)cyclopropyl](thiophen-2-yl)methanone* (**2k**). 3-(5-Chloro-2-hydroxyphenyl)-1-(thiophen-2-yl)prop-2-en-1-one **1k** (175 mg, 0.66 mmol). Yield 135 mg (73% yield); pale yellow solid; mp 112–113 °C. R*_f_* 0.58 (petroleum ether:ethyl acetate 3:1). ^1^H NMR (CDCl_3_, 500 MHz) *δ* = 1.53 (ddd, ^3^*J =* 8.2 Hz, ^3^*J =* 6.8 Hz, ^2^*J =* 4.1 Hz, 1H, CH_2_), 1.84 (ddd, ^3^*J =* 9.0 Hz, ^3^*J =* 5.1 Hz, ^2^*J =* 4.1 Hz, 1H, CH_2_), 2.72 (ddd, ^3^*J =* 8.2 Hz, ^3^*J =* 5.1 Hz, ^3^*J =* 4.2 Hz, 1H, CH), 2.81 (ddd, ^3^*J =* 9.0 Hz, ^3^*J =* 6.8 Hz, ^3^*J =* 4.2 Hz, 1H, CH), 6.16 (s, 1H, OH), 6.79 (d, ^3^*J =* 8.5 Hz, 1H, Ar), 6.99 (d, ^4^*J =* 2.5 Hz, 1H, Ar), 7.08 (dd, ^3^*J =* 8.5 Hz, ^4^*J =* 2.5 Hz, 1H, Ar), 7.13 (dd, ^3^*J =* 4.9 Hz, ^3^*J =* 3.8 Hz, 1H, Th), 7.64 (dd, ^3^*J =* 4.9 Hz, ^4^*J =* 1.1 Hz, 1H, Th), 7.83 (d, ^3^*J =* 3.8 Hz; ^4^*J =* 1.1 Hz, 1H, Th). ^1^H NMR (DMSO-*d*_6_, 500 MHz) *δ* = 1.54–1.62 (m, 2H, CH_2_), 2.69 (ddd, ^3^*J =* 9.0 Hz, ^3^*J =* 7.0 Hz, ^3^*J =* 4.2 Hz, 1H, CH), 3.07 (ddd, ^3^*J =* 8.0 Hz, ^3^*J =* 5.1 Hz, ^3^*J =* 4.2 Hz, 1H, CH), 6.78–6.82 (m, 1H, Ar), 7.03–7.09 (m, 2H, Ar), 7.25 (dd, ^3^*J =* 4.9 Hz, ^3^*J =* 3.8 Hz, 1H, Th), 8.02 (dd, ^3^*J =* 4.9 Hz, ^4^*J =* 1.1 Hz, 1H, Th), 8.20 (dd, ^3^*J =* 3.8 Hz, ^4^*J =* 0.9 Hz, 1H, Th), 9.80 (s, 1H, OH). ^13^C NMR (CDCl_3_, 126 MHz) *δ* = 17.6, 23.8, 28.0, 117.0, 125.3, 127.3, 127.7, 128.0, 128.5, 132.6, 134.3, 144.4, 154.0, 191.7. IR (cm^−1^) 3480, 3405, 3105, 1620, 1515, 1500, 1415, 1340, 1270, 1245, 1110, 970, 820, 720. HRMS ESI-TOF: *m*/*z* 301.0063 [M + Na]^+^ (301.0060 calcd for C_14_H_11_ClNaO_2_S^+^). Anal. calcd for C_14_H_11_ClO_2_S: C, 60.32; H, 3.98. Found: C, 60.18; H, 3.87.

*[(1RS,2RS)-2-(5-Bromo-2-hydroxyphenyl)cyclopropyl](thiophen-2-yl)methanone* (**2l**). 3-(5-Bromo-2-hydroxyphenyl)-1-(thiophen-2-yl)prop-2-en-1-one **1l** (273 mg, 0.90 mmol). Yield 182 mg (64% yield); colorless solid; mp 117–118 °C. R*_f_* 0.40 (petroleum ether:ethyl acetate 3:1). ^1^H NMR (CDCl_3_, 500 MHz) *δ* = 1.51–1.58 (m, 1H, CH_2_), 1.80–1.86 (m, 1H, CH_2_), 2.74–2.81 (m, 1H, CH), 2.81–2.89 (m, 1H, CH), 5.96 (s, 1H, OH), 6.74 (d, ^3^*J =* 8.5 Hz, 1H, Ar), 7.14–7.16 (m, 1H, Ar), 7.19–7.25 (m, 1H, Ar), 7.40–7.48 (m, 2H, Ar), 7.52–7.60 (m, 1H, Ar), 7.93–8.02 (m, 2H, Ar). ^1^H NMR (DMSO-*d*_6_, 500 MHz) *δ* = 1.53–1.61 (m, 2H, CH_2_), 2.68 (ddd, ^3^*J =* 9.0 Hz, ^3^*J =* 7.0 Hz, ^3^*J =* 4.2 Hz, 1H, CH), 3.03–3.09 (m, 1H, CH), 6.75 (d, ^3^*J =* 8.4 Hz, 1H, Ar), 7.15–7.21 (m, 2H, Ar), 7.25 (dd, ^3^*J =* 5.0 Hz, ^3^*J =* 3.8 Hz, 1H, Th), 8.02 (dd, ^3^*J =* 5.0 Hz, ^4^*J =* 1.1 Hz, 1H, Th), 8.20 (dd, ^3^*J =* 3.8 Hz, ^4^*J =* 1.1 Hz, 1H, Th), 9.83 (s, 1H, OH). ^13^C NMR (CDCl_3_, 126 MHz) *δ* = 17.8, 24.0, 27.1, 112.6, 117.4, 128.3 (2C), 128.4, 128.8 (2C), 130.3, 131.0, 133.4, 137.5, 154.5, 199.4. IR (cm^−1^) 3210, 2980, 1625, 1590, 1445, 1405, 1390, 1240, 1165, 1030, 1000, 790, 730. HRMS ESI-TOF: *m*/*z* 317.0166/319.0147 [M + H]^+^ (317.0172/319.0152 calcd for C_16_H_14_^79^BrO_2_^+^/C_16_H_14_^81^BrO_2_^+^).

*[(1RS,2RS)-2-(2-Hydroxy-5-nitrophenyl)cyclopropyl](thiophen-2-yl)methanone* (**2m**). 3-(2-Hydroxy-5-nitrophenyl)-1-(thiophen-2-yl)prop-2-en-1-one **1m** (206 mg, 0.75 mmol). Yield 180 mg (83% yield); yellow solid; mp 196–197 °C. R*_f_* 0.74 (petroleum ether:ethyl acetate 3:1). ^1^H NMR (DMSO-*d*_6_, 500 MHz) *δ* = 1.57–1.61 (m, 1H, CH_2_), 1.86–1.89 (m, 1H, CH_2_), 2.73–2.75 (m, 1H, CH), 2.88–2.92 (m, 1H, CH), 6.91 (d, ^3^*J =* 8.8 Hz, 1H, Ar), 7.15 (dd, ^3^*J =* 4.9 Hz, ^3^*J =* 3.8 Hz, 1H, Th), 7.29 (s, 1H, OH), 7.65 (dd, ^3^*J =* 4.9 Hz, ^4^*J =* 1.0 Hz, 1H, Th), 7.83 (dd, ^3^*J =* 3.8 Hz, ^4^*J =* 1.0 Hz, 1H, Th), 7.88 (d, ^4^*J =* 2.6 Hz, 1H, Ar), 7.97 (dd, ^3^*J =* 8.9 Hz, ^4^*J =* 2.6 Hz, 1H, Ar). ^13^C NMR (DMSO-*d*_6_, 126 MHz) *δ* = 17.3, 23.0, 27.4, 114.9, 122.2, 124.0, 127.6, 128.9, 133.8, 135.1, 139.7, 144.2, 162.6, 190.8. IR (cm^−1^) 3230, 2925, 1610, 1590, 1525, 1500, 1410, 1340, 1285, 1240, 1090, 1060, 990, 835, 735. HRMS ESI-TOF: *m*/*z* 312.0300 [M + Na]^+^ (312.0301 calcd for C_14_H_11_NaNO_4_S^+^).

*[(1RS,2RS)-2-(2-Hydroxy-3-methoxyphenyl)cyclopropyl](phenyl)methanone* (**2n**). 3-(2-Hydroxy-3-methoxyphenyl)-1-(phenyl)prop-2-en-1-one **1n** (254 mg, 1.00 mmol). Yield 147 mg (55% yield); colorless oil. R*_f_* 0.54 (petroleum ether:ethyl acetate 3:1). ^1^H NMR (CDCl_3_, 500 MHz) *δ* = 1.59–1.66 (m, 1H, CH_2_), 1.92 (ddd, ^3^*J =* 9.0 Hz, ^3^*J =* 5.2 Hz, ^2^*J =* 3.8 Hz, 1H, CH_2_), 2.89 (ddd, ^3^*J =* 9.0 Hz, ^3^*J =* 6.8 Hz, ^3^*J =* 4.2 Hz, 1H, CH), 2.95 (ddd, ^3^*J =* 7.9 Hz, ^3^*J =* 5.2 Hz, ^3^*J =* 4.2 Hz, 1H, CH), 3.90 (s, 3H, OCH_3_), 5.88 (s, 1H, OH), 6.70–6.71 (m, 1H, Ar), 6.79–6.86 (m, 2H, Ar), 7.46–7.49 (m, 2H, Ar), 7.55–7.58 (m, 1H, Ar), 8.06–8.07 (m, 2H, Ar). ^13^C NMR (CDCl_3_, 126 MHz) *δ* = 17.6 (CH_2_), 25.4 (CH), 27.5 (CH), 56.1 (CH_3_), 109.1 (CH), 118.9 (CH), 119.5 (CH), 126.1 (C), 128.2 (2 × CH), 128.5 (2 × CH), 132.7 (CH), 138.1 (C), 144.8 (C), 146.5 (C), 199.4 (CO). IR (cm^−1^) 3220, 2985, 1640, 1570, 1530, 1465, 1405, 1310, 1270, 1225, 1070, 860, 755, 735. Anal. calcd for C_17_H_16_O_3_: C, 76.10; H, 6.01. Found: C, 76.28; H, 5.97.

*[(1RS,2RS)-2-(3-Ethoxy-2-hydroxyphenyl)cyclopropyl](phenyl)methanone* (**2o**). 3-(3-Ethoxy-2-hydroxyphenyl)-1-(phenyl)prop-2-en-1-one **1o** (537 mg, 2.00 mmol). Yield 368 mg (65% yield); colorless solid; mp 80–81 °C. R*_f_* 0.71 (petroleum ether:ethyl acetate 4:1). ^1^H NMR (CDCl_3_, 500 MHz) *δ* = 1.33 (t, ^3^*J =* 7.0 Hz, 3H, OCH_2_C*H*_3_), 1.55 (ddd, ^3^*J =* 7.9 Hz, ^3^*J =* 6.9 Hz, ^2^*J =* 3.6 Hz, 1H, CH_2_), 1.68 (ddd, ^3^*J =* 9.0 Hz, ^3^*J =* 5.1 Hz, ^2^*J =* 3.6 Hz, 1H, CH_2_), 2.72 (ddd, ^3^*J =* 9.0 Hz, ^3^*J =* 6.9 Hz, ^3^*J =* 4.1 Hz, 1H, CH), 3.01 (ddd, ^3^*J =* 7.9 Hz, ^3^*J =* 5.1 Hz, ^3^*J =* 4.1 Hz, 1H, CH), 4.02 (q, ^3^*J =* 7.0 Hz, 2H, OC*H*_2_CH_3_), 6.63–6.66 (m, 1H, Ar), 6.69–6.73 (m, 1H, Ar), 6.80–6.84 (m, 1H, Ar), 7.50–7.55 (m, 2H, Ar), 7.60–7.66 (m, 1H, Ar), 8.00–8.05 (m, 2H, Ar), 8.42 (s, 1H, OH). ^13^C NMR (DMSO-*d_6_*, 125 MHz) *δ* = 14.7, 17.6, 24.8, 27.2, 64.1, 111.2, 117.7, 118.8, 126.6, 127.9 (2C), 128.7 (2C), 133.0, 137.3, 145.3, 146.3, 198.3. IR (cm^−1^) 3390, 2995, 1670, 1615, 1590, 1495, 1470, 1400, 1350, 1275, 1225, 1175, 1070, 1005, 960, 775, 755, 720, 690. Anal. calcd for C_18_H_18_O_3_: C, 76.57; H, 6.43. Found: C, 76.58; H, 6.41.

*[(1RS,2RS)-2-(3-Ethoxy-2-hydroxyphenyl)cyclopropyl](thiophen-2-yl)methanone* (**2p**). 3-(3-Ethoxy-2-hydroxyphenyl)-1-(2-thienyl)prop-2-en-1-one **1p** (549 mg, 2.00 mmol). Yield 388 mg (67% yield); colorless solid; mp 77–78 °C. R*_f_* 0.52 (petroleum ether:ethyl acetate 4:1). ^1^H NMR (DMSO-*d*_6_, 500 MHz) *δ* = 1.34 (t, ^3^*J_CH_ =* 7.0 Hz, 3H, OCH_2_C*H*_3_), 1.54 (ddd, ^3^*J =* 8.0 Hz, ^3^*J =* 7.0 Hz, ^2^*J =* 3.7 Hz, 1H, CH_2_), 1.63 (ddd, ^3^*J =* 9.0 Hz, ^3^*J =* 5.1 Hz, ^2^*J =* 3.7 Hz, 1H, CH_2_), 2.75 (ddd, ^3^*J =* 9.0 Hz, ^3^*J =* 7.0 Hz, ^3^*J =* 4.1 Hz, 1H, CH), 2.95 (ddd, ^3^*J =* 8.0 Hz, ^3^*J =* 5.1 Hz, ^3^*J =* 4.1 Hz, 1H, CH), 4.03 (q, ^3^*J =* 7.0 Hz, 2H, OC*H*_2_CH_3_), 6.62–6.64 (m, 1H, Ar), 6.70–6.73 (m, 1H, Ar), 6.81–6.83 (m, 1H, Ar), 7.24 (dd, ^3^*J =* 3.8 Hz, ^3^*J =* 5.0 Hz, 1H, Th), 8.00 (dd, ^3^*J =* 5.0 Hz, ^4^*J =* 1.2 Hz, 1H, Th), 8.20 (dd, ^3^*J =* 3.8 Hz, ^4^*J =* 1.2 Hz, 1H, Th), 8.42 (s, 1H, OH). ^13^C NMR (DMSO-*d*_6_, 125 MHz): *δ* 14.7 (OCH_2_*C*H_3_), 17.4 (CH_2_), 24.1 (CH), 27.6 (CH), 64.1 (O*C*H_2_CH_3_), 111.3 (CH), 117.7 (CH), 118.8 (CH), 126.5 (C), 133.2 (CH), 134.7 (CH), 144.4 (C), 145.3 (C), 146.3 (C), 191.0 (CO). IR (cm^−1^) 3360, 3105, 2980, 1630, 1605, 1470, 1415, 1350, 1275, 1245, 1220, 1185, 1070, 860, 775, 730. Anal. calcd for C_16_H_16_O_3_S: C, 66.64; H, 5.59. Found: C, 66.67; H, 5.52.

## 4. Conclusions

To conclude, we developed a simple general approach to (het)aryl 2-(2-hydroxyaryl)cyclopropyl ketones **2** based on the Corey–Chaykovsky cyclopropanation of 2-hydroxychalcones, determined the scope and limitations of this reaction. The obtained D–A cyclopropanes are promising building blocks for the synthesis of diverse heterocyclic compounds and bioactive substances.

## Supplementary Materials

Copies of NMR spectra for novel compounds are available online.
