# Supplementary material for: Synthesis of (Het)aryl 2-(2-hydroxyaryl)cyclopropyl Ketones
Source: Molecules. 2020 Dec 5;25(23):5748. doi: 10.3390/molecules25235748 (PMC7729819; doi:10.3390/molecules25235748)

Synthesis of (Het)aryl 2-(2-Hydroxyaryl)Cyclopropyl Ketones

Alexander A. Fadeev [1,2], Alexey O. Chagarovskiy [3,4], Anton S. Makarov [2], Irina I. Levina [5], Olga A. Ivanova [3,6], Maxim G. Uchuskin [2,*] and Igor V. Trushkov [3,4,*]

1 Department of Organic Chemistry, Faculty of Science, Charles University, Hlavova 8, 12800 Prague 2, Czech Republic; fadeev@fadeeva.natur.cuni.cz
2 Department of Chemistry, Perm State University, Bukireva 15, Perm 614990, Russian Federation; antony.s.makarov@psu.ru
3 N. D. Zelinsky Institute of Organic Chemistry, Leninsky pr. 47, Moscow 119991, Russian Federation
4 Dmitry Rogachev National Medical Research Center of Pediatric Hematology, Oncology and Immunology, Samory Mashela 1, Moscow 117997, Russian Federation; alex.chagarovskiy@gmail.com
5 N.M. Emanuel Institute of Biochemical Physcis Russian Academy of Sciences, Kosygina 4, Moscow 117198, Russian Federation; iilevina@inbox.ru
6 Department of Chemistry, M. V. Lomonosov Moscow State University, Leninskie gory 1-3, Moscow 119991, Russian Federation; iv@kinet.chem.msu.ru
* Correspondence: mu@psu.ru (M.G.U.); itrushkov@mail.ru (I.V.T.); Tel.: +7-916-645-9951 (I.V.T.)

SUPPORTING INFORMATION

Copies of NMR spectra

(E)-3-(5-Fluoro-2-hydroxyphenyl)-1-(4-methoxyphenyl)prop-2-en-1-one (**1d**)

^{1}H NMR (500 MHz, DMSO-d_6)

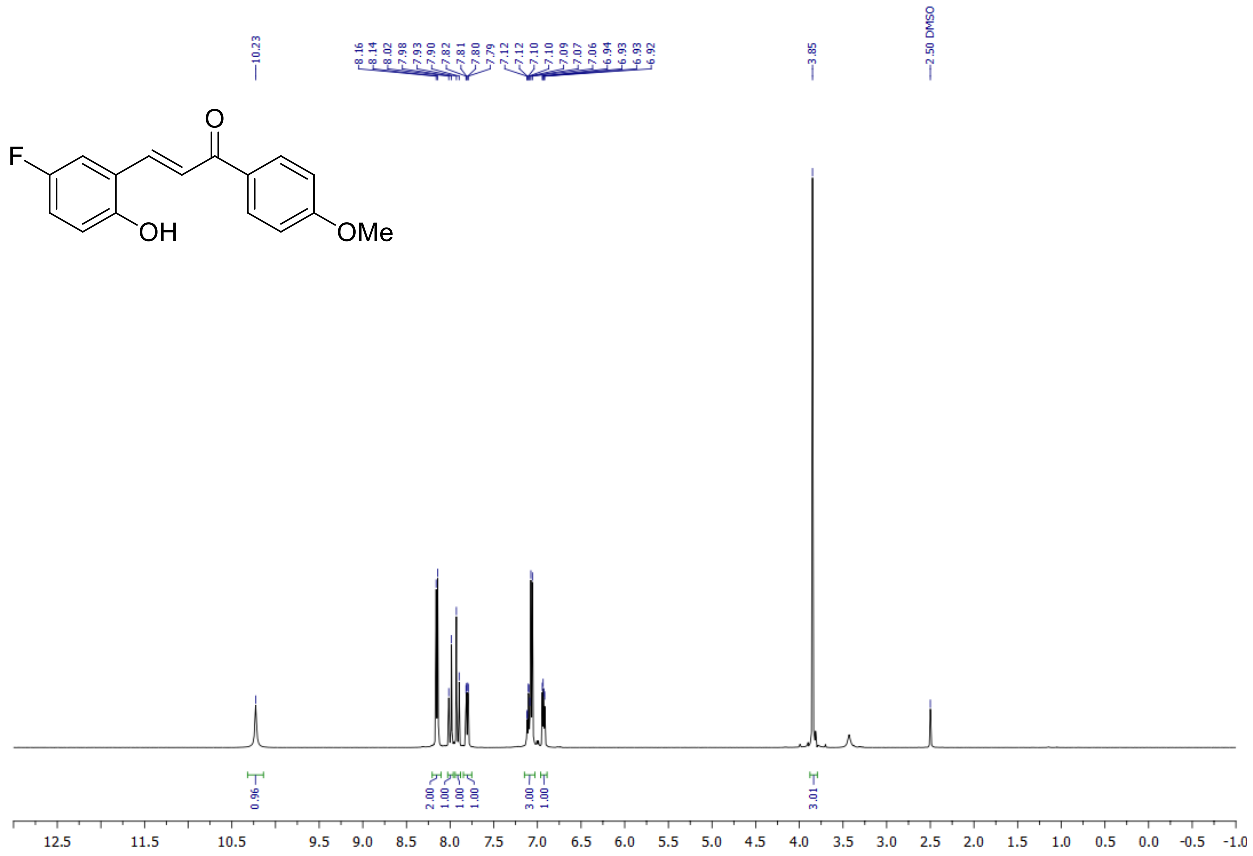

^{13}C NMR (126 MHz, DMSO-d_6)

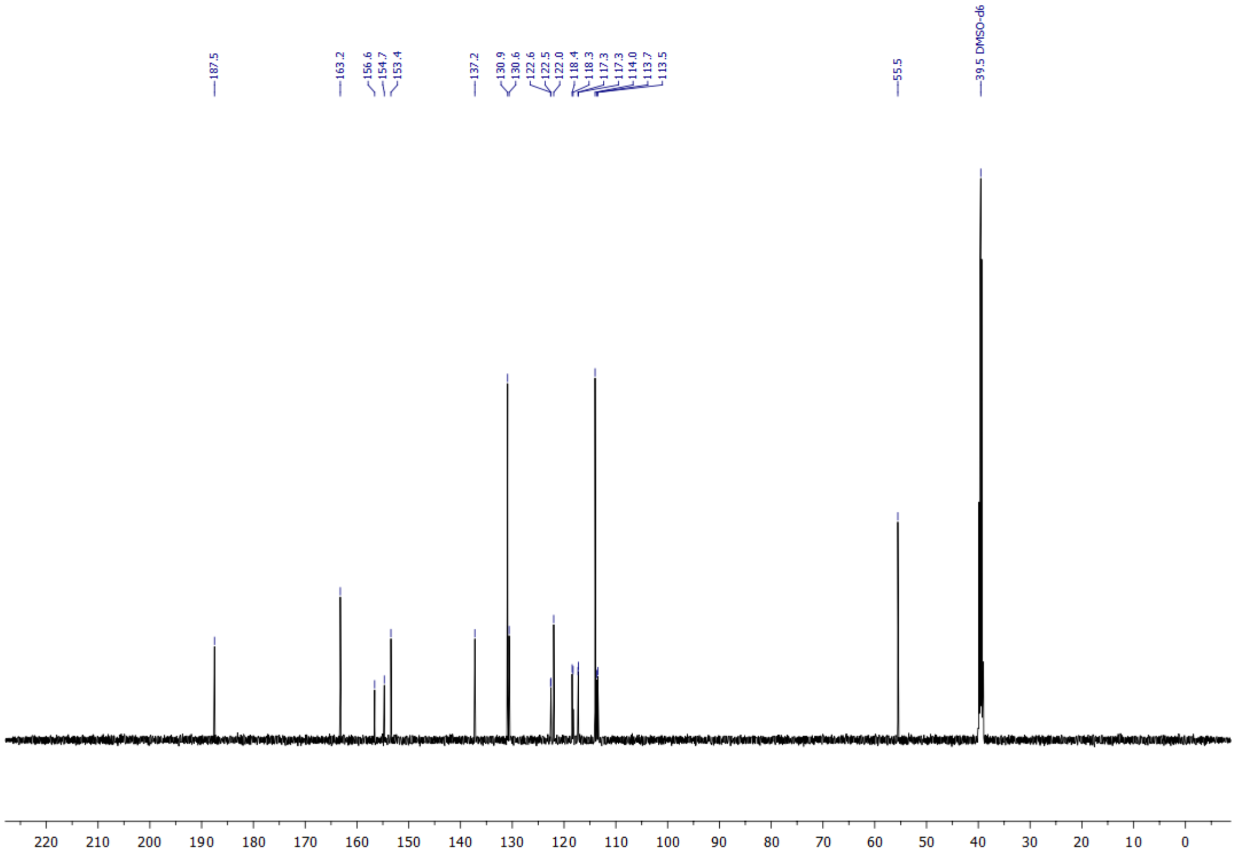

(E)-3-(5-Fluoro-2-hydroxyphenyl)-1-(4-methoxyphenyl)prop-2-en-1-one (**1d**)
^{19}F NMR (471 MHz, DMSO-d_6)

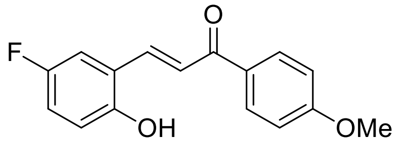

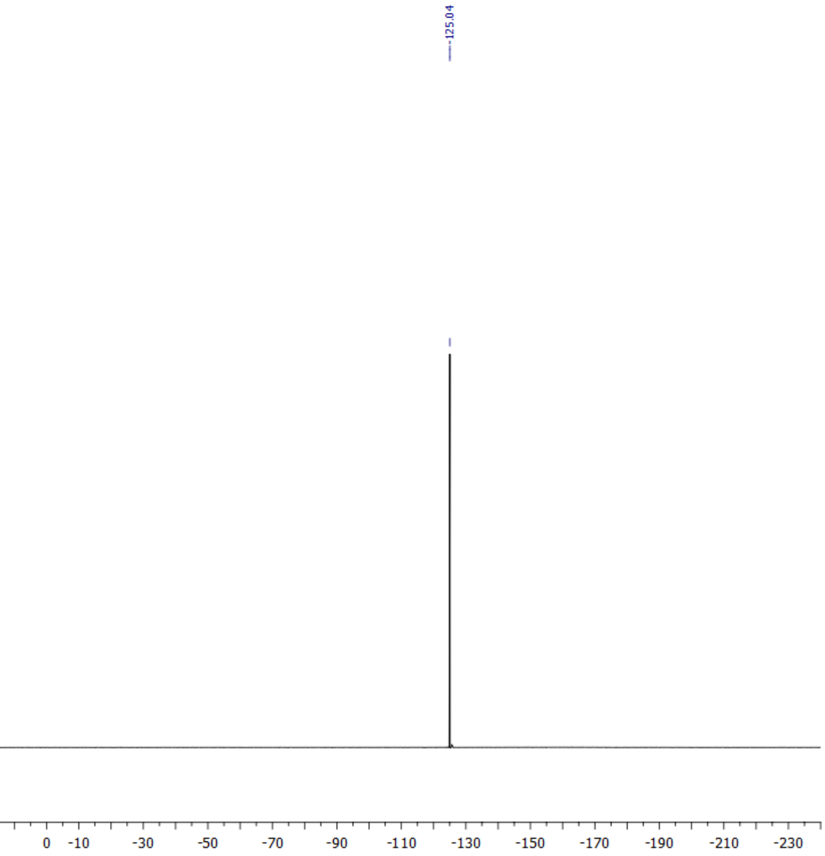

***(E)-3-(2-Hydroxy-5-nitrophenyl)-1-(4-methoxyphenyl)prop-2-en-1-one* (1f)**

^{1}H NMR (500 MHz, DMSO-d_6)

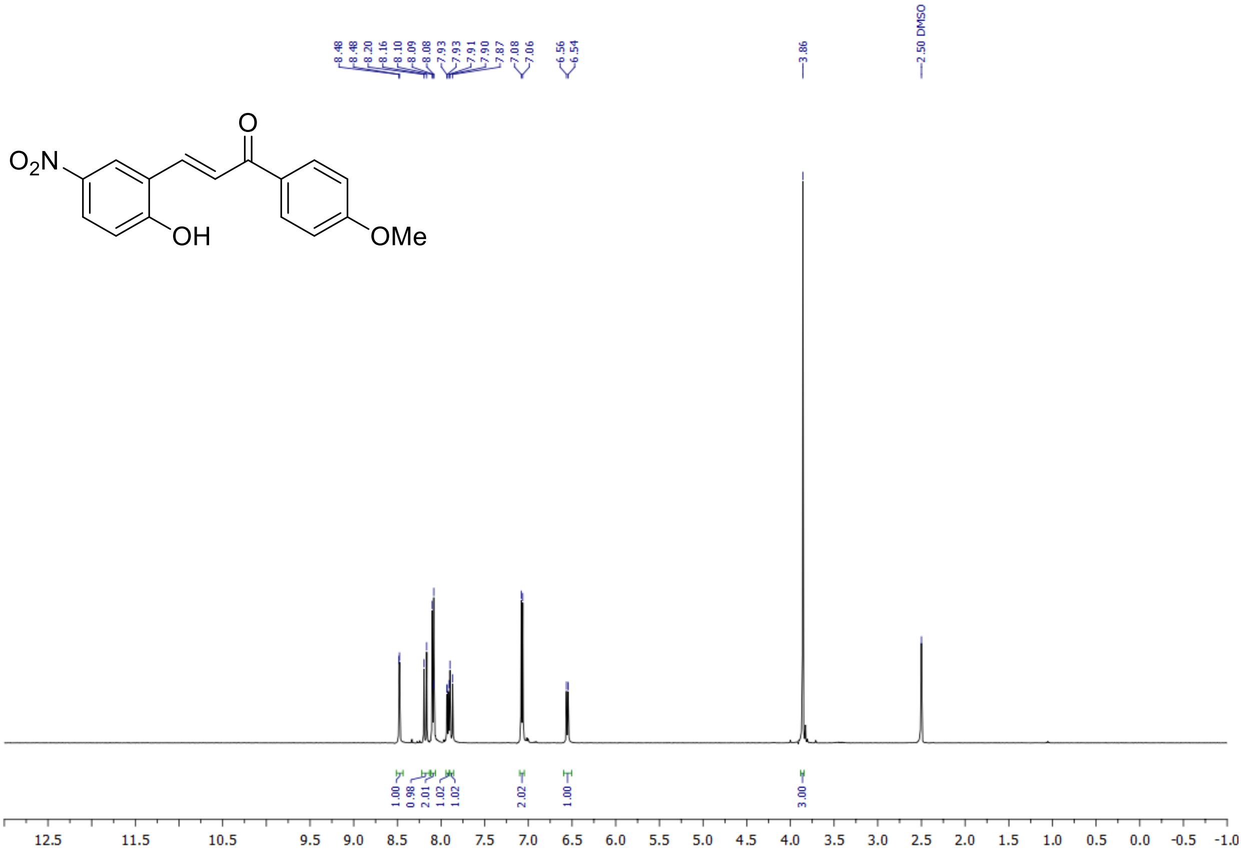

^{13}C NMR (126 MHz, DMSO-d_6)

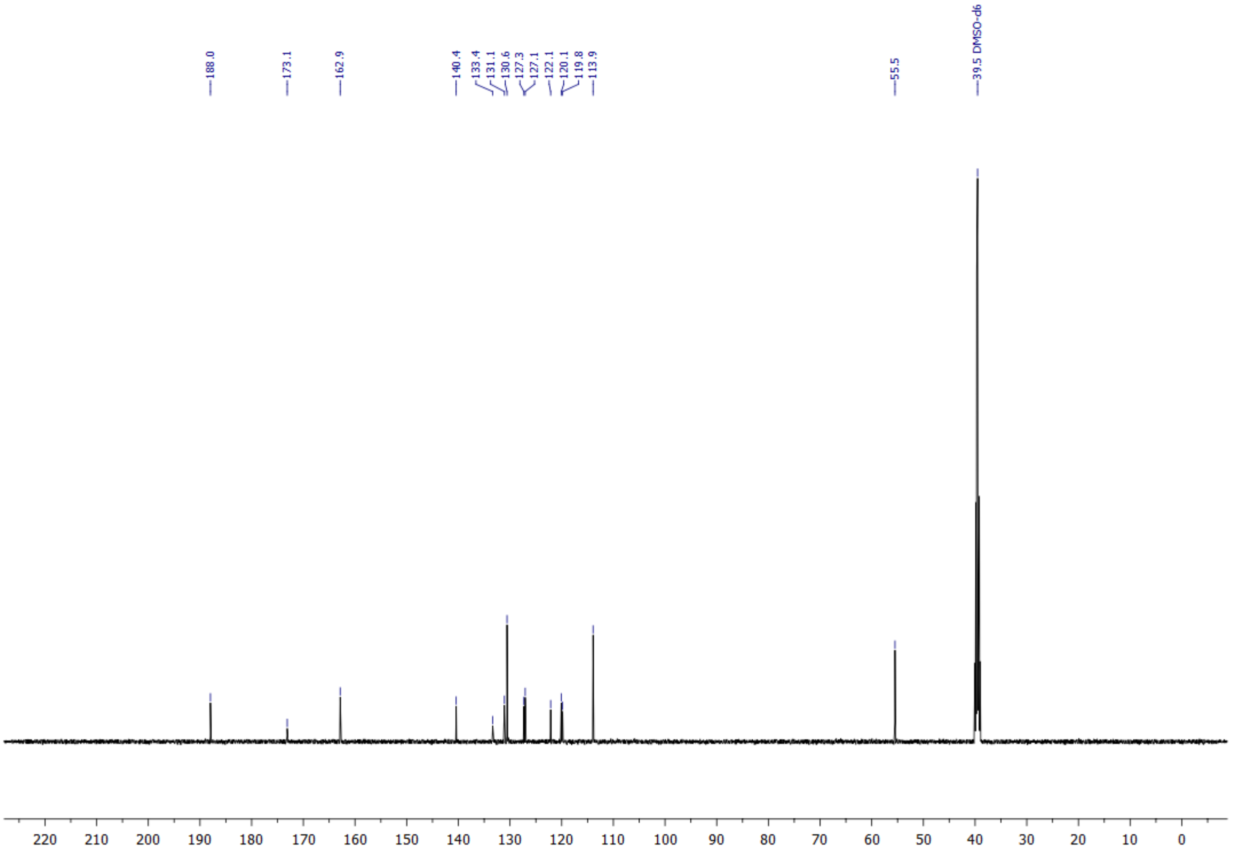

***(E)*-3-(5-Fluoro-2-hydroxyphenyl)-1-phenylprop-2-en-1-one** (**1g**)

^{1}H NMR (500 MHz, DMSO-d_6)

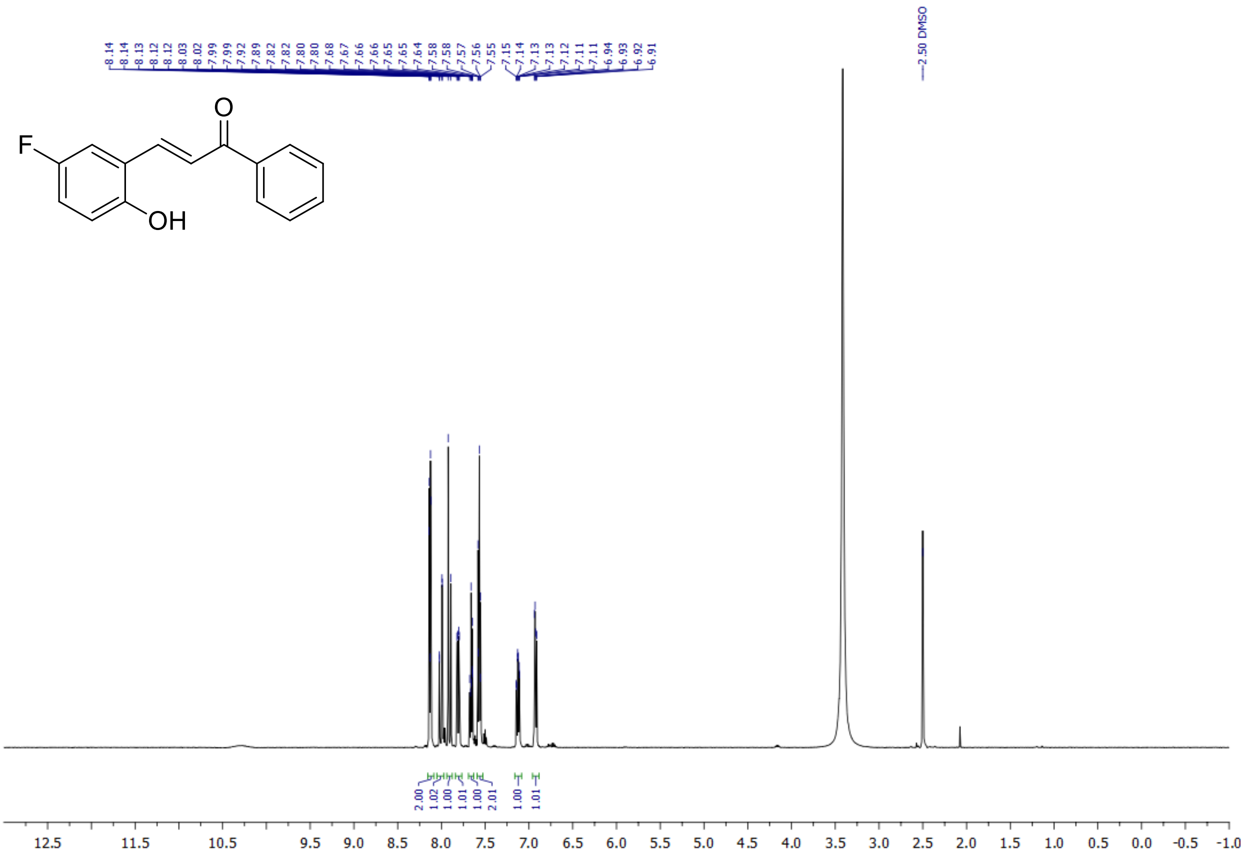

^{13}C NMR (126 MHz, DMSO-d_6)

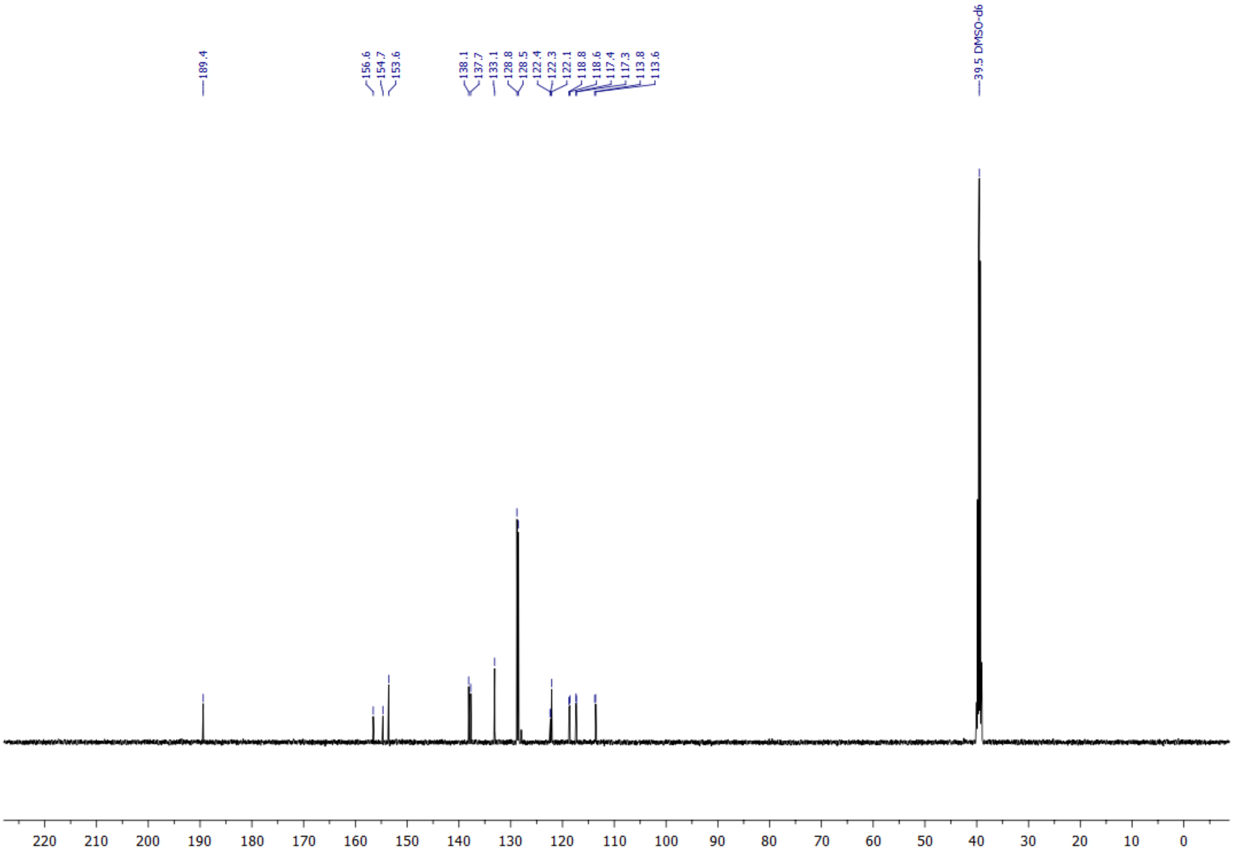

***(E)*-3-(5-Fluoro-2-hydroxyphenyl)-1-phenylprop-2-en-1-one** (**1g**)

^{19}F NMR (471 MHz, DMSO-d_6)

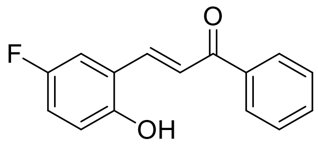

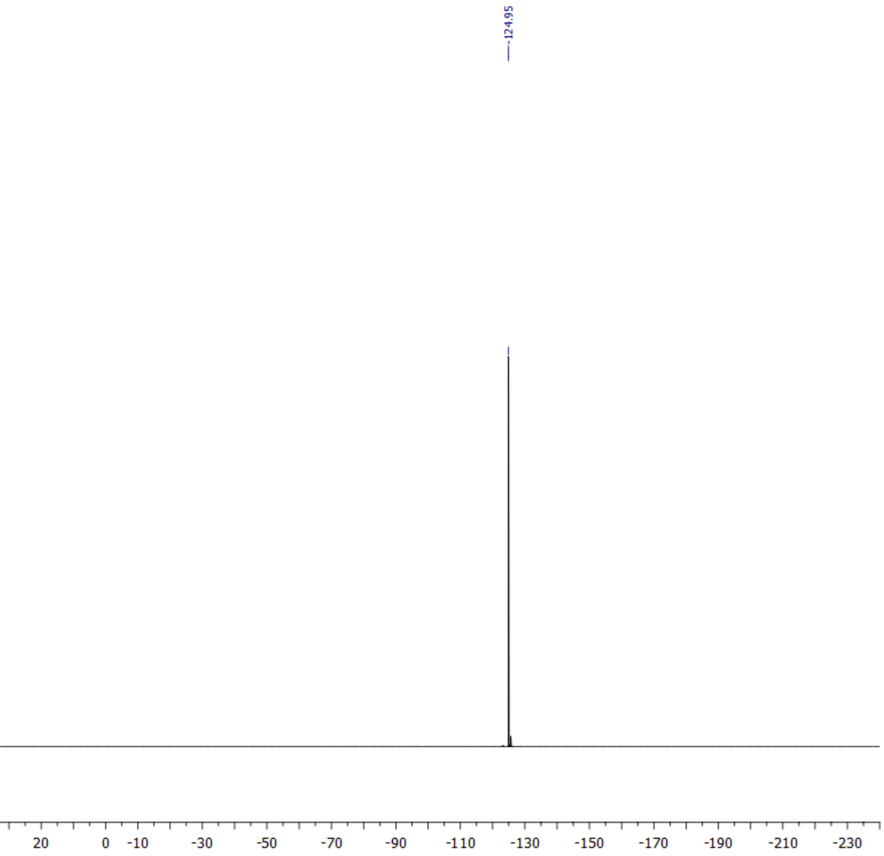

(E)-3-(5-Fluoro-2-hydroxyphenyl)-1-(2-thienyl)prop-2-en-1-one (**1j**)

^{1}H NMR (500 MHz, DMSO-d_6)

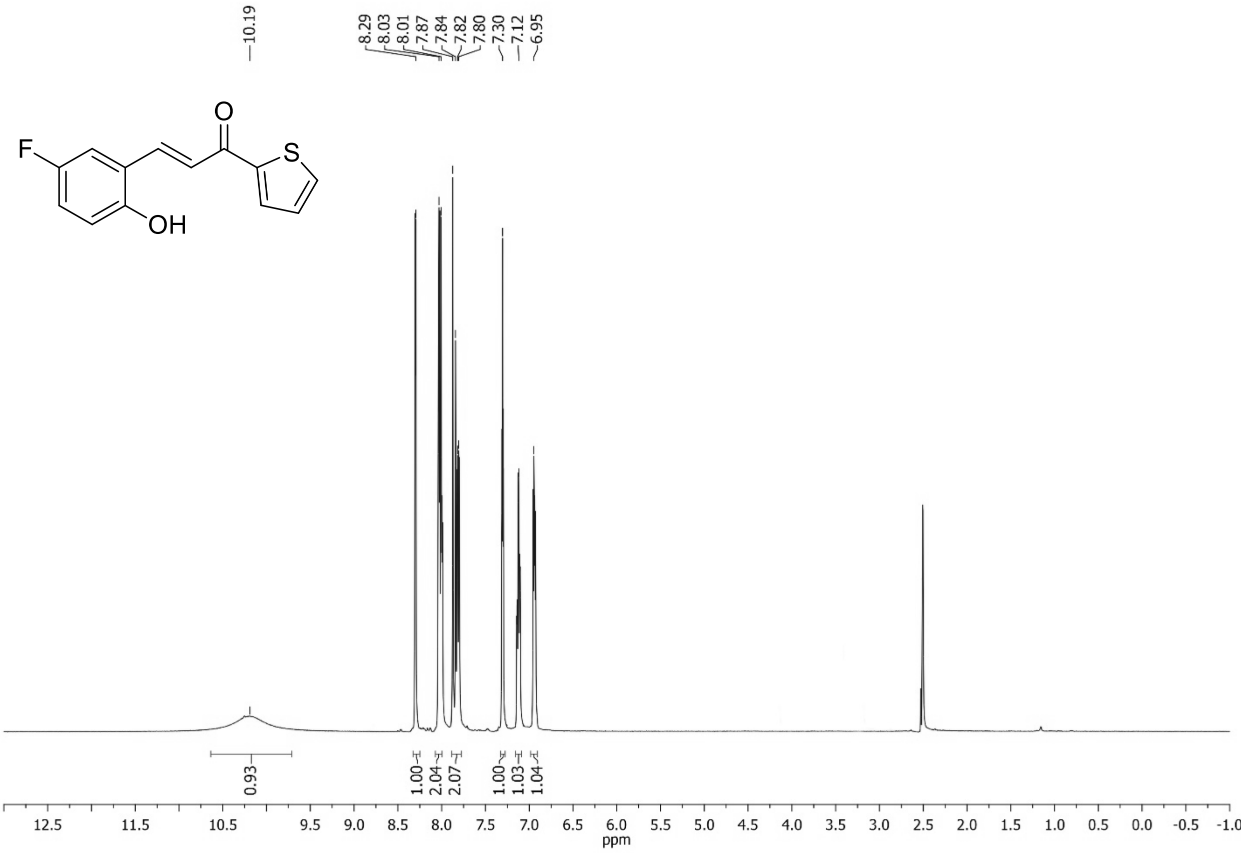

^{13}C NMR (126 MHz, DMSO-d_6)

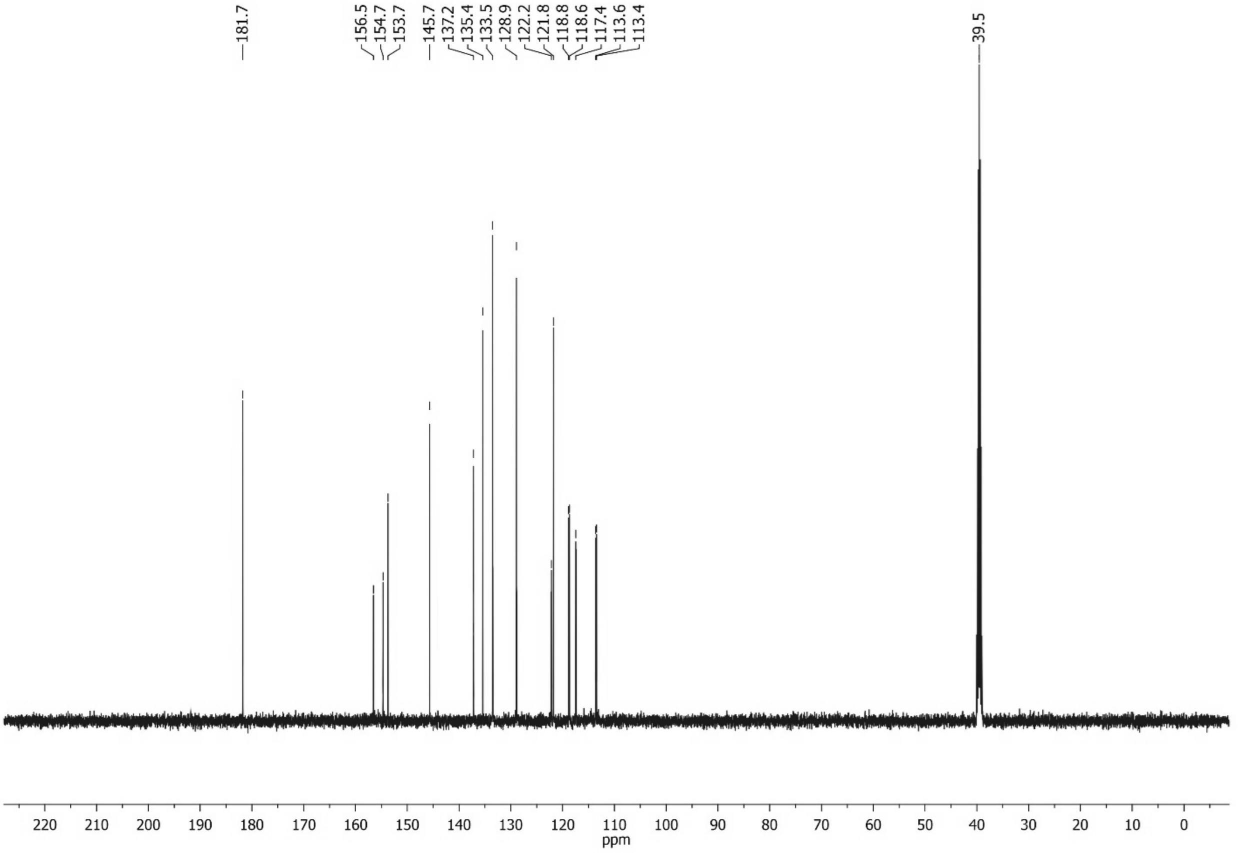

(E)-3-(5-Fluoro-2-hydroxyphenyl)-1-(2-thienyl)prop-2-en-1-one (**1j**)

^{19}F NMR (471 MHz, DMSO-*d$_6$*)

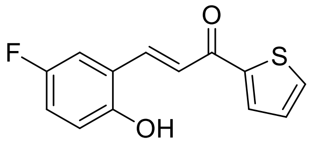

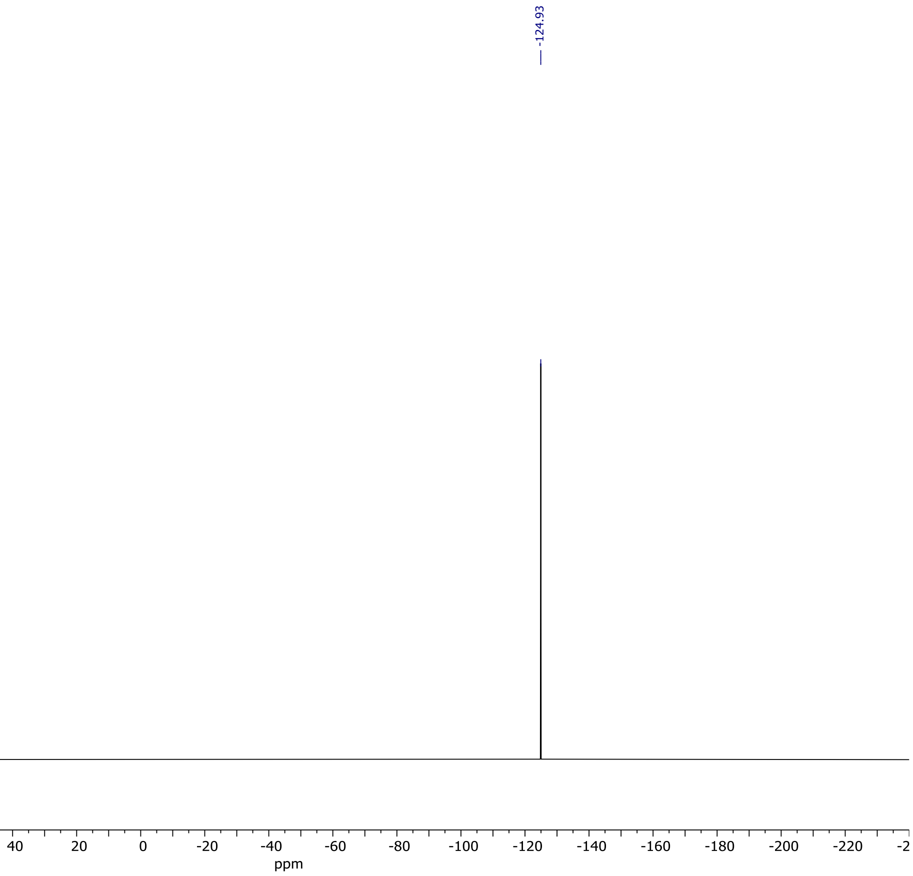

***(E)*-3-(5-Chloro-2-hydroxyphenyl)-1-(2-thienyl)prop-2-en-1-one** (**1k**)

^{1}H NMR (500 MHz, DMSO-d_6)

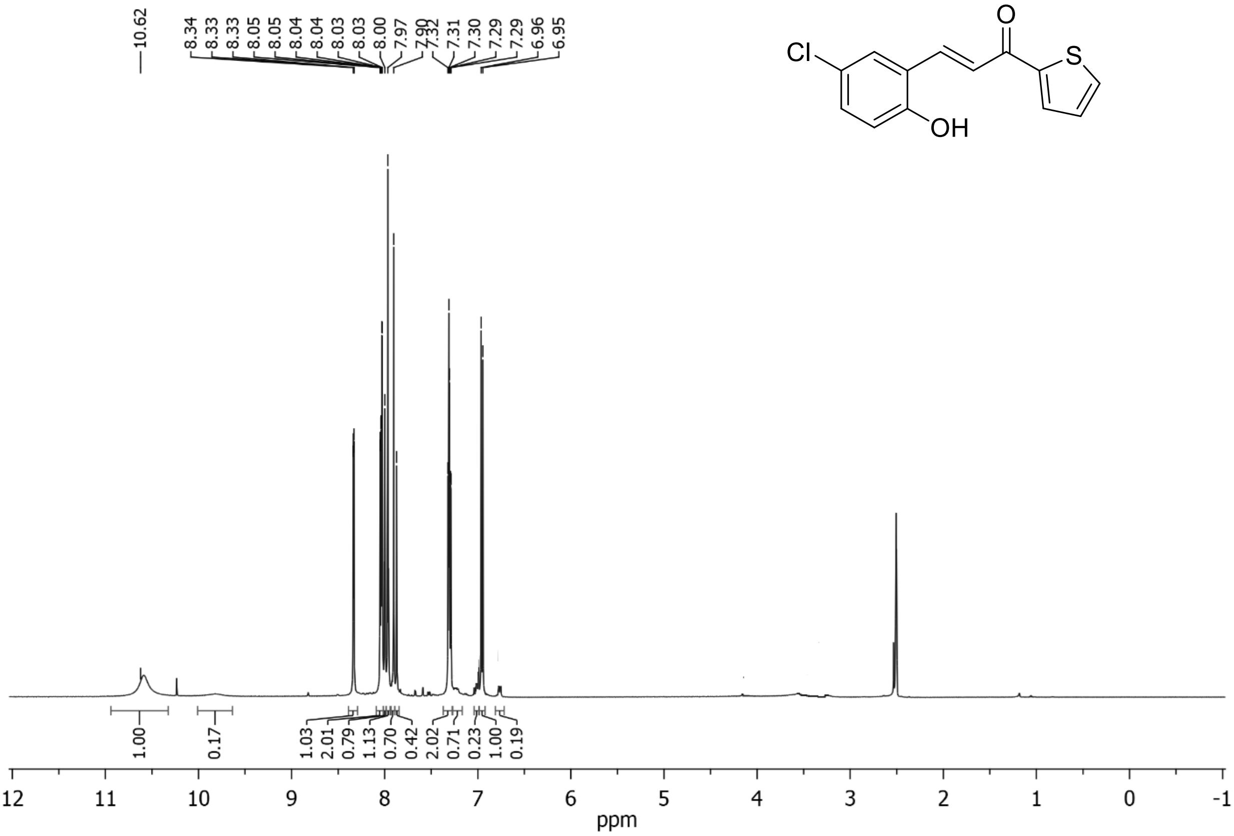

^{13}C NMR (126 MHz, DMSO-d_6)

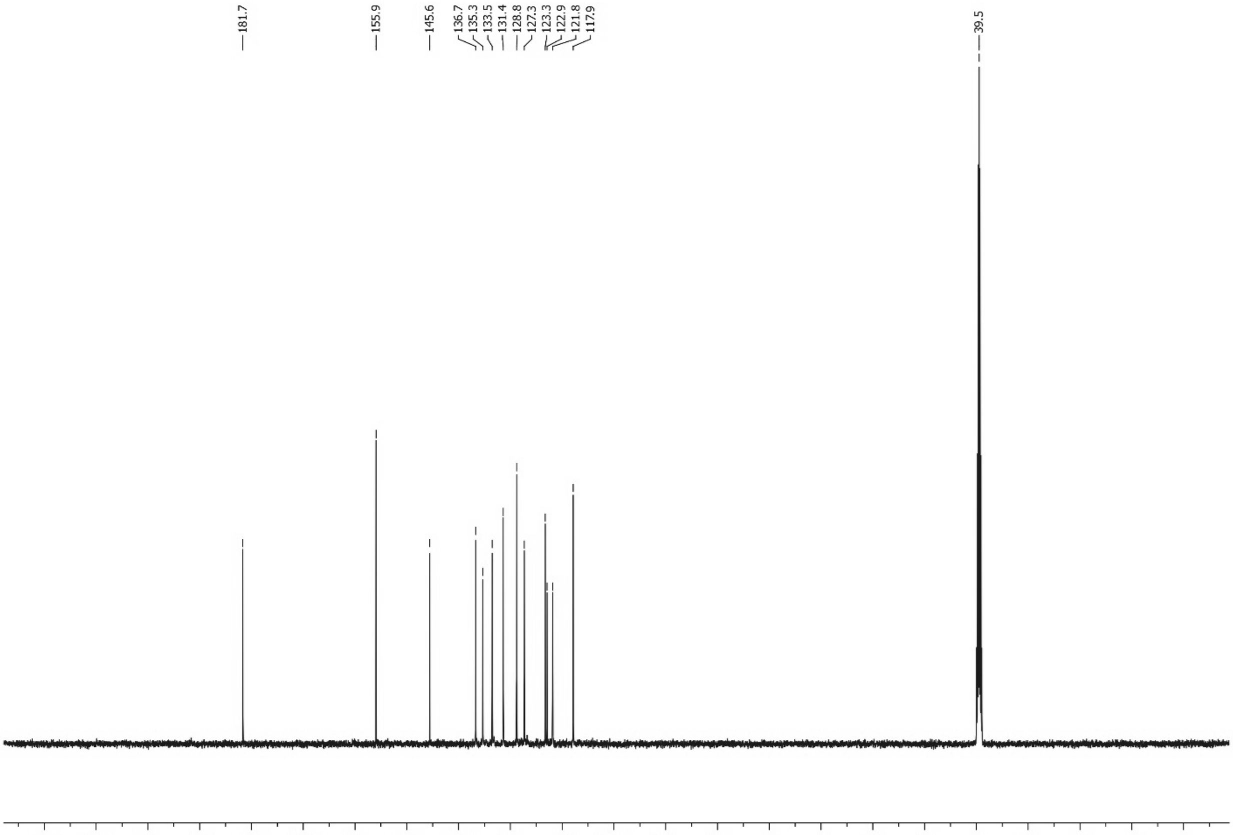

(E)-3-(5-Bromo-2-hydroxyphenyl)-1-(2-thienyl)prop-2-en-1-one (**1l**)

^{1}H NMR (500 MHz, DMSO-d_6)

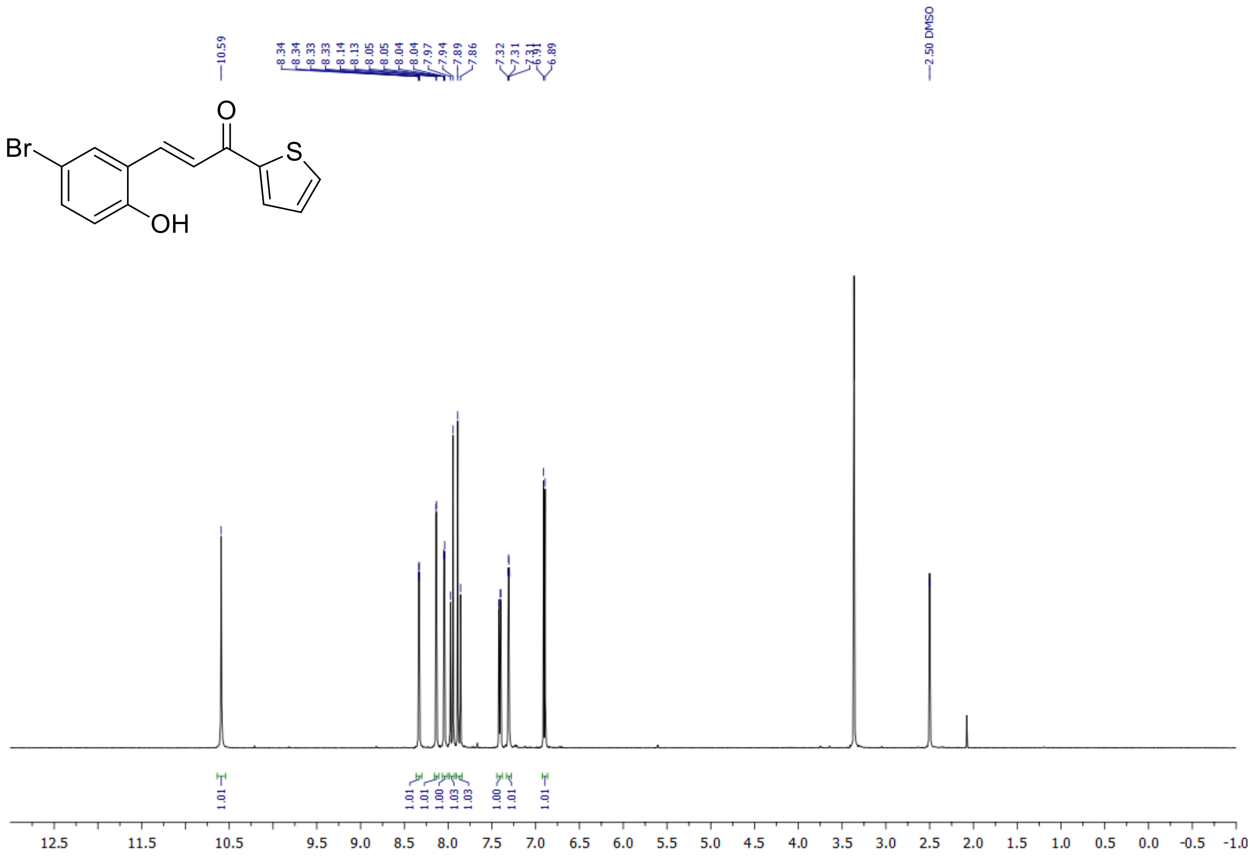

^{13}C NMR (126 MHz, DMSO-d_6)

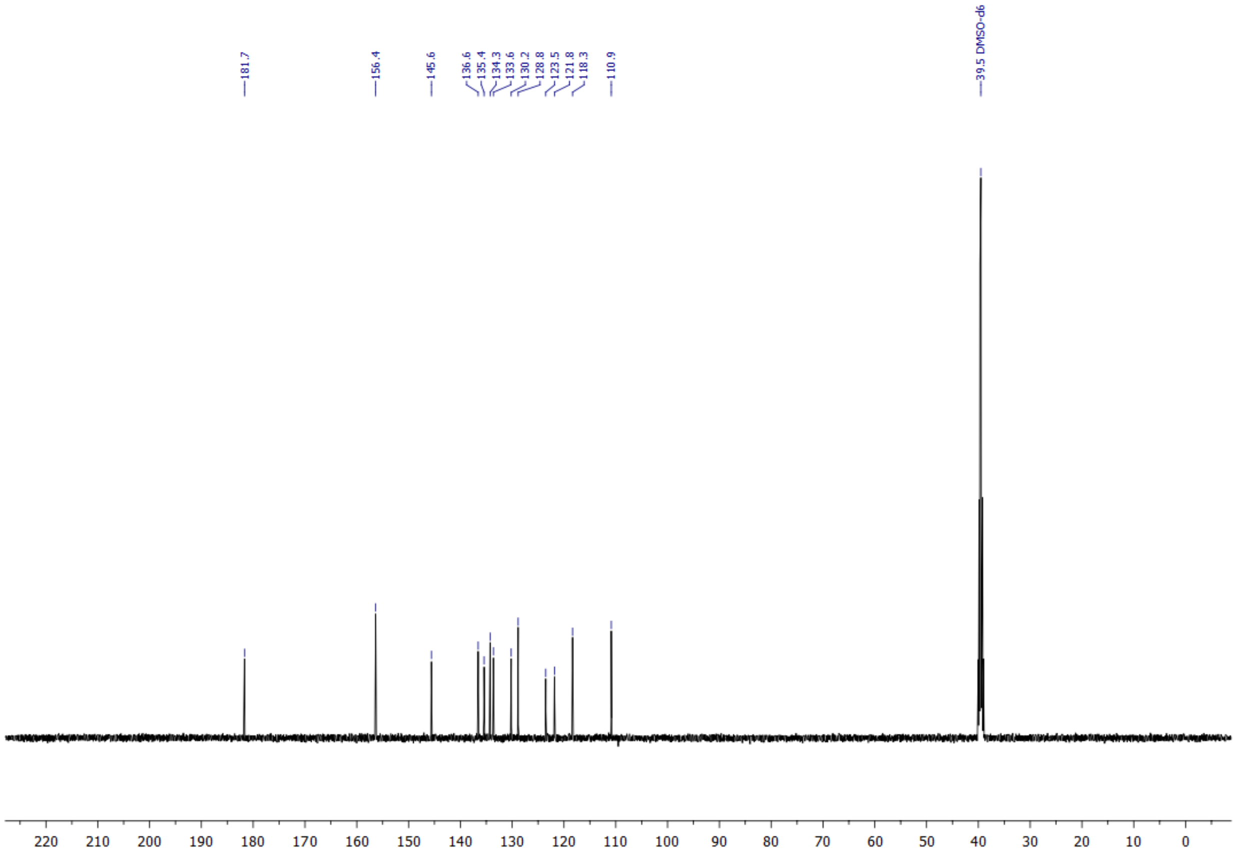

***(E)*-3-(2-Hydroxy-5-nitrophenyl)-1-(2-thienyl)prop-2-en-1-one** (**1m**)

^{1}H NMR (500 MHz, DMSO-d_6)

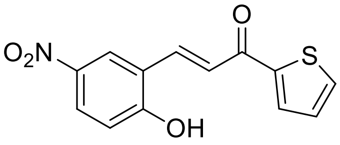

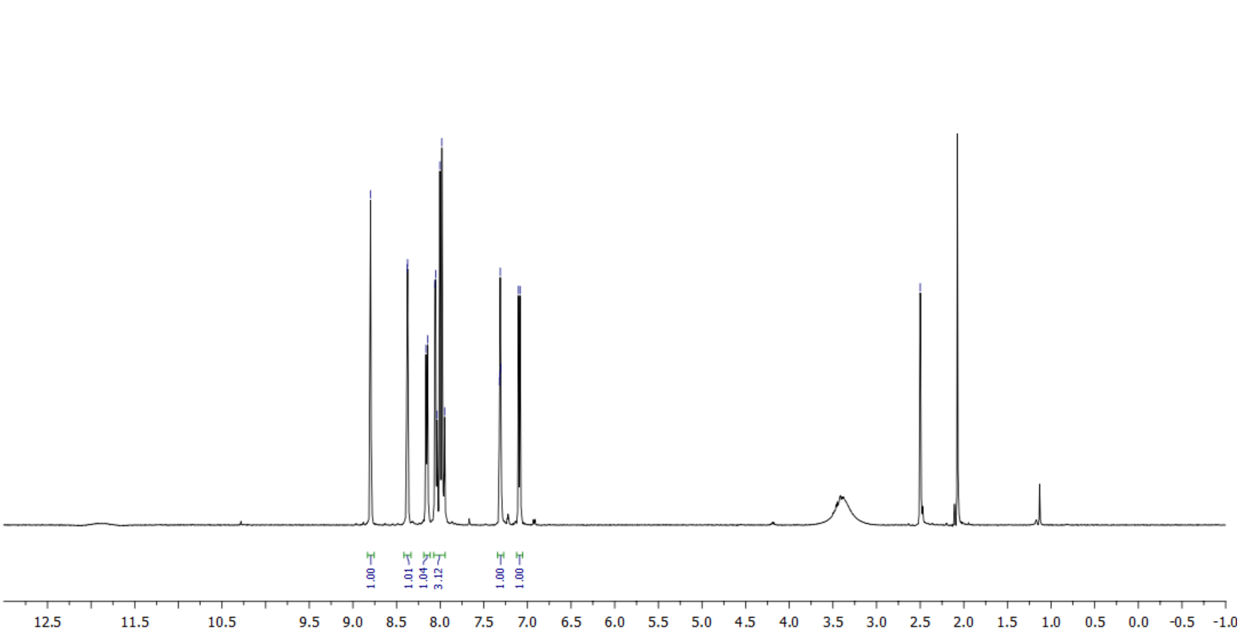

^{13}C NMR (126 MHz, DMSO-d_6)

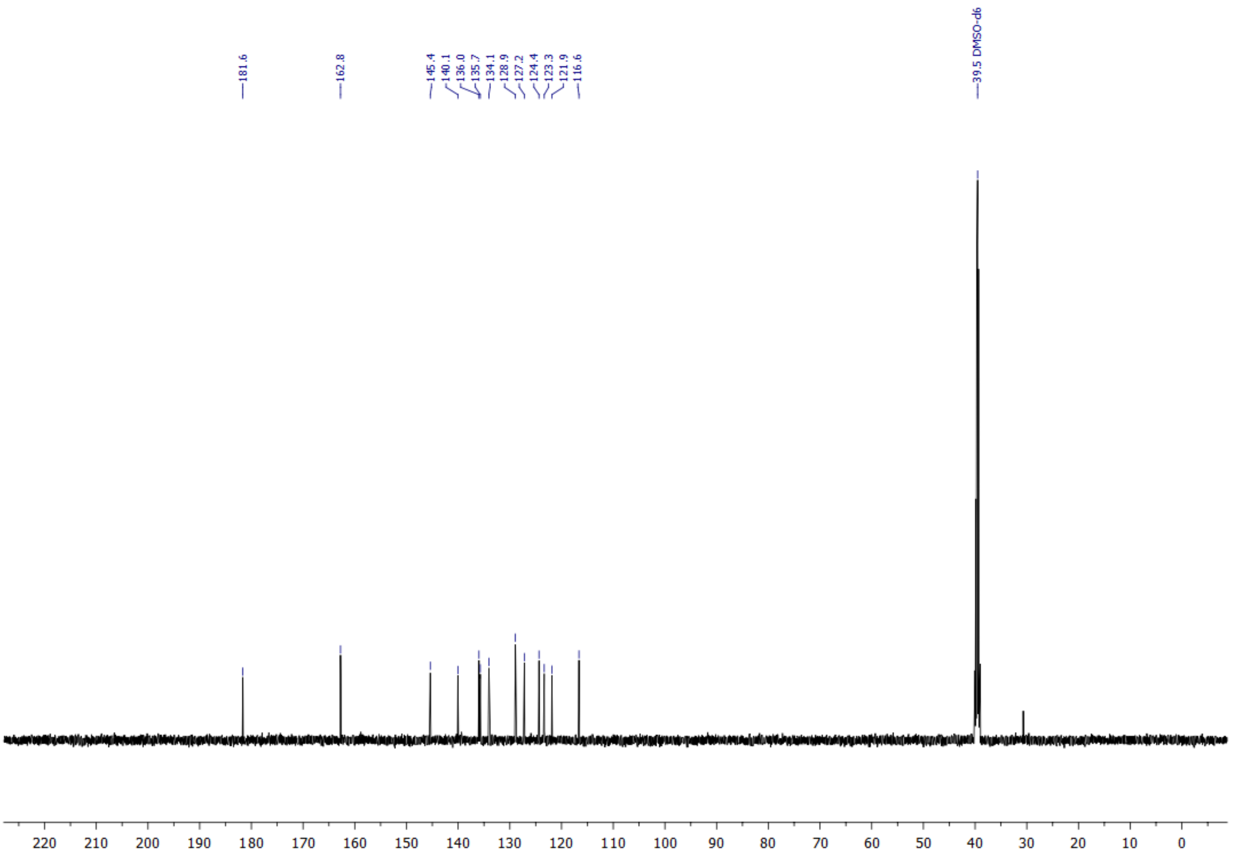

(E)-3-(3-Ethoxy-2-hydroxyphenyl)-1-phenylprop-2-en-1-one (**1o**)

^{1}H NMR (500 MHz, DMSO-d_6)

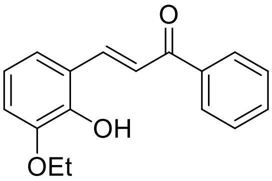

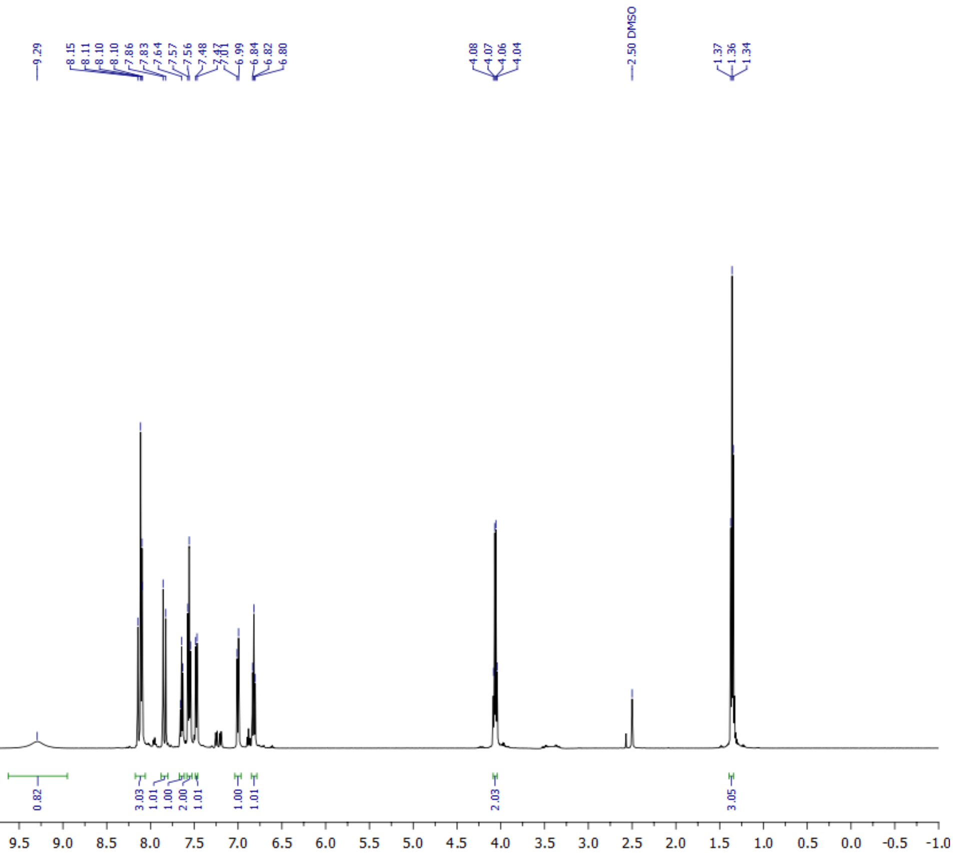

^{13}C NMR (126 MHz, DMSO-d_6)

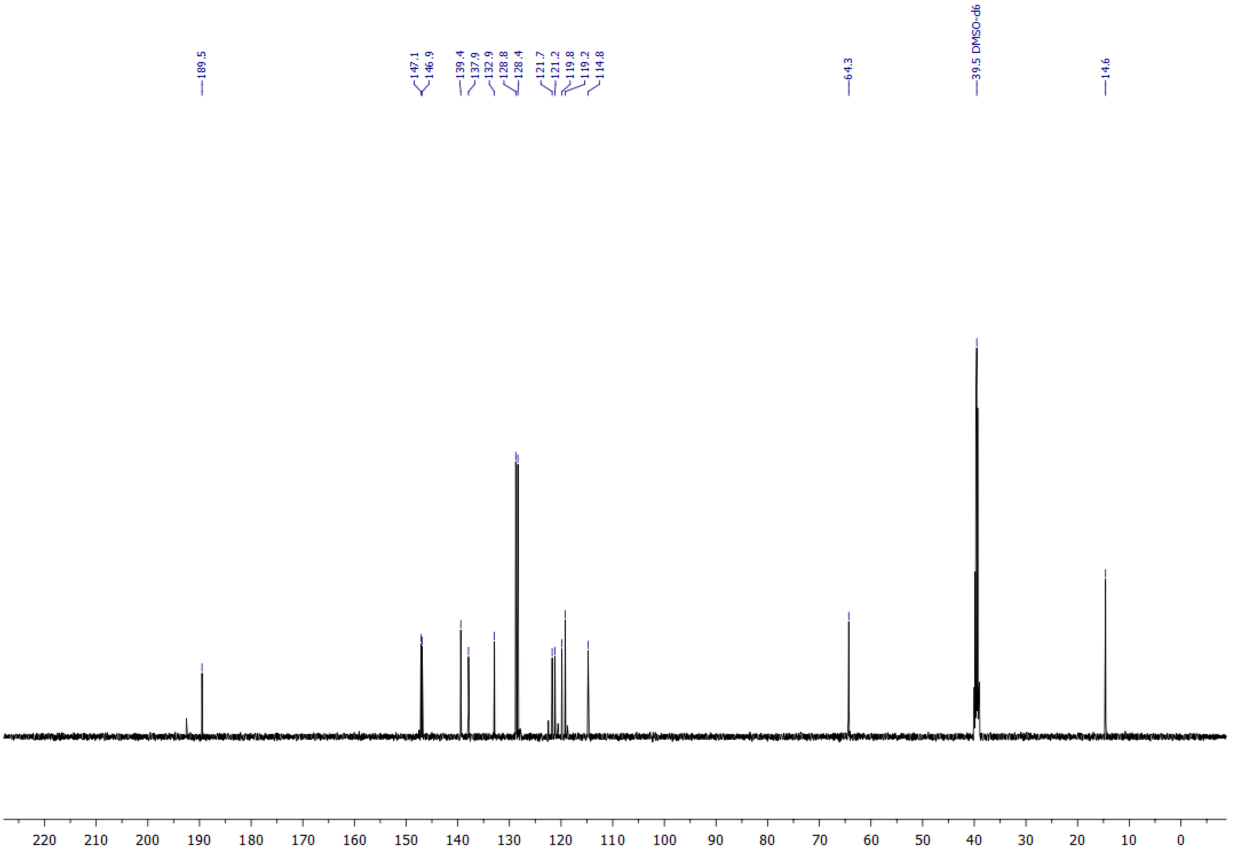

(E)-3-(3-Ethoxy-2-hydroxyphenyl)-1-(2-thienyl)prop-2-en-1-one (**1p**)

^{1}H NMR (500 MHz, DMSO-d_6)

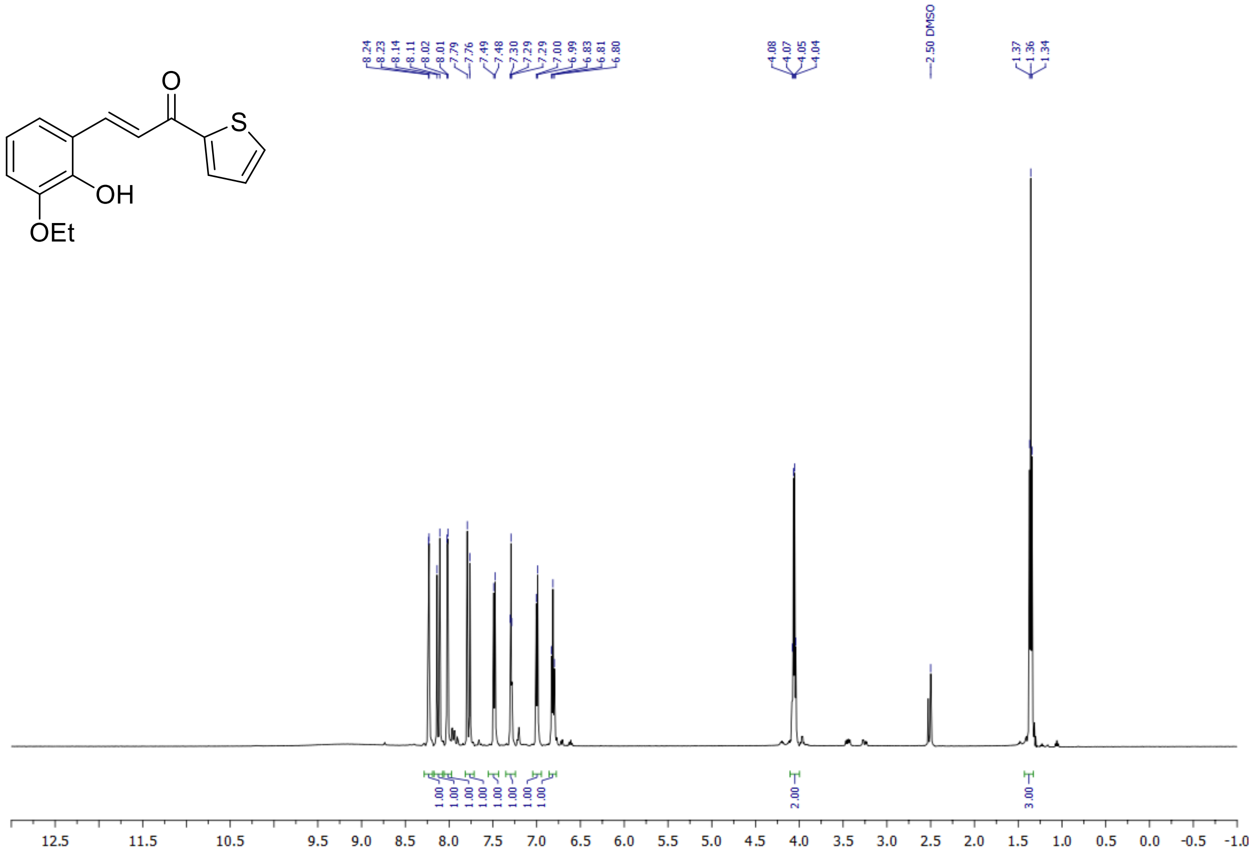

^{13}C NMR (126 MHz, DMSO-d_6)

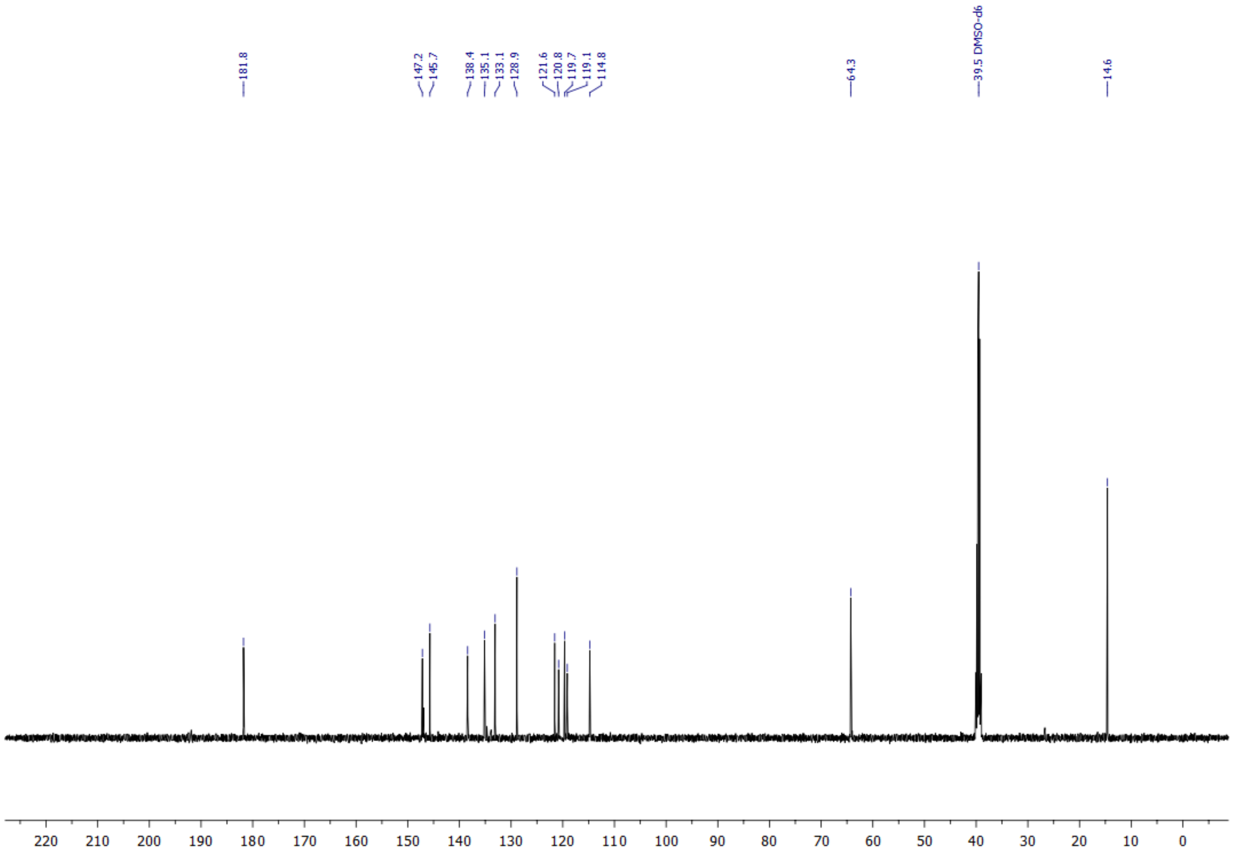

[(1RS,2RS)-2-(2-Hydroxyphenyl)cyclopropyl](phenyl)methanone (**2a**)

^{1}H NMR (500 MHz, $CDCl_3$)

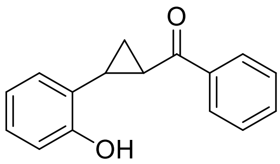

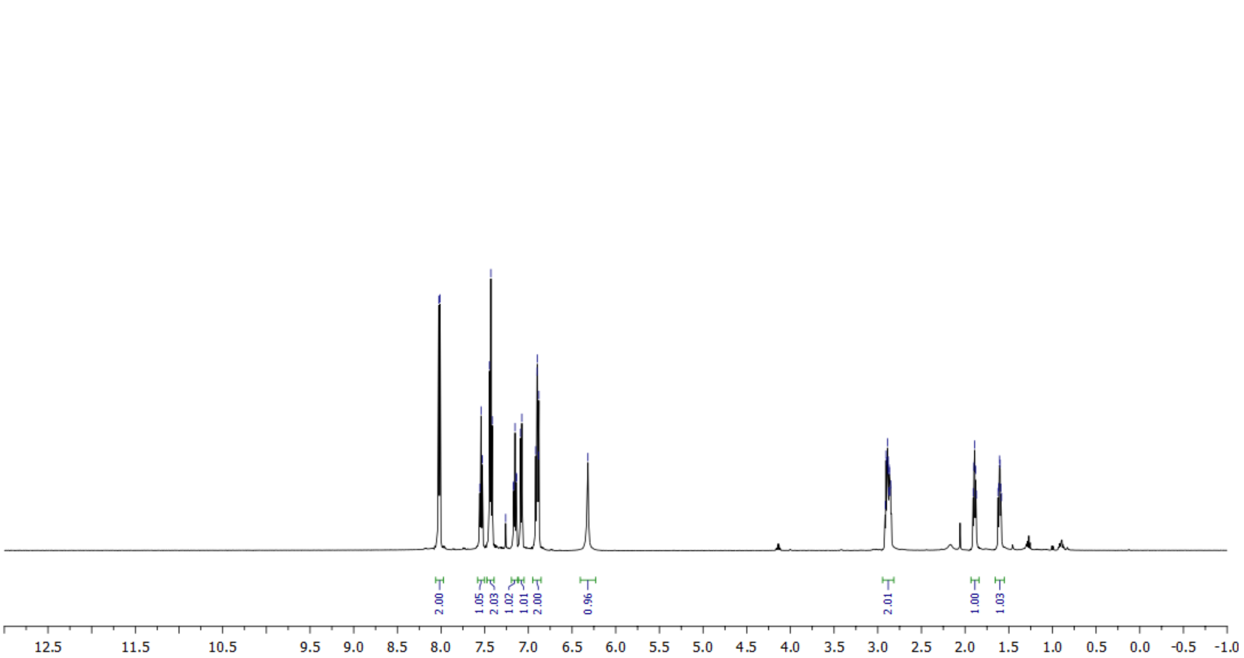

^{1}H NMR (500 MHz, DMSO-d_6)

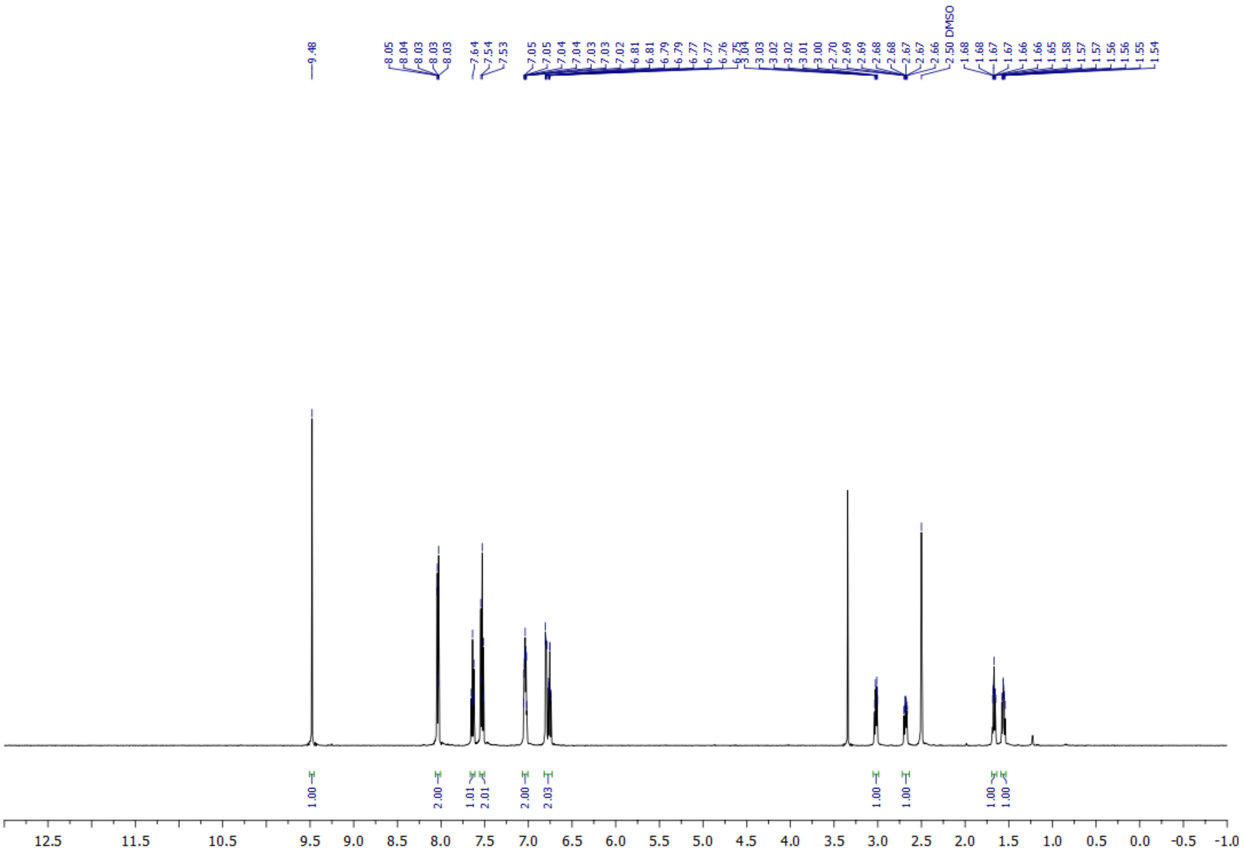

^{13}C NMR (126 MHz, $CDCl_3$)

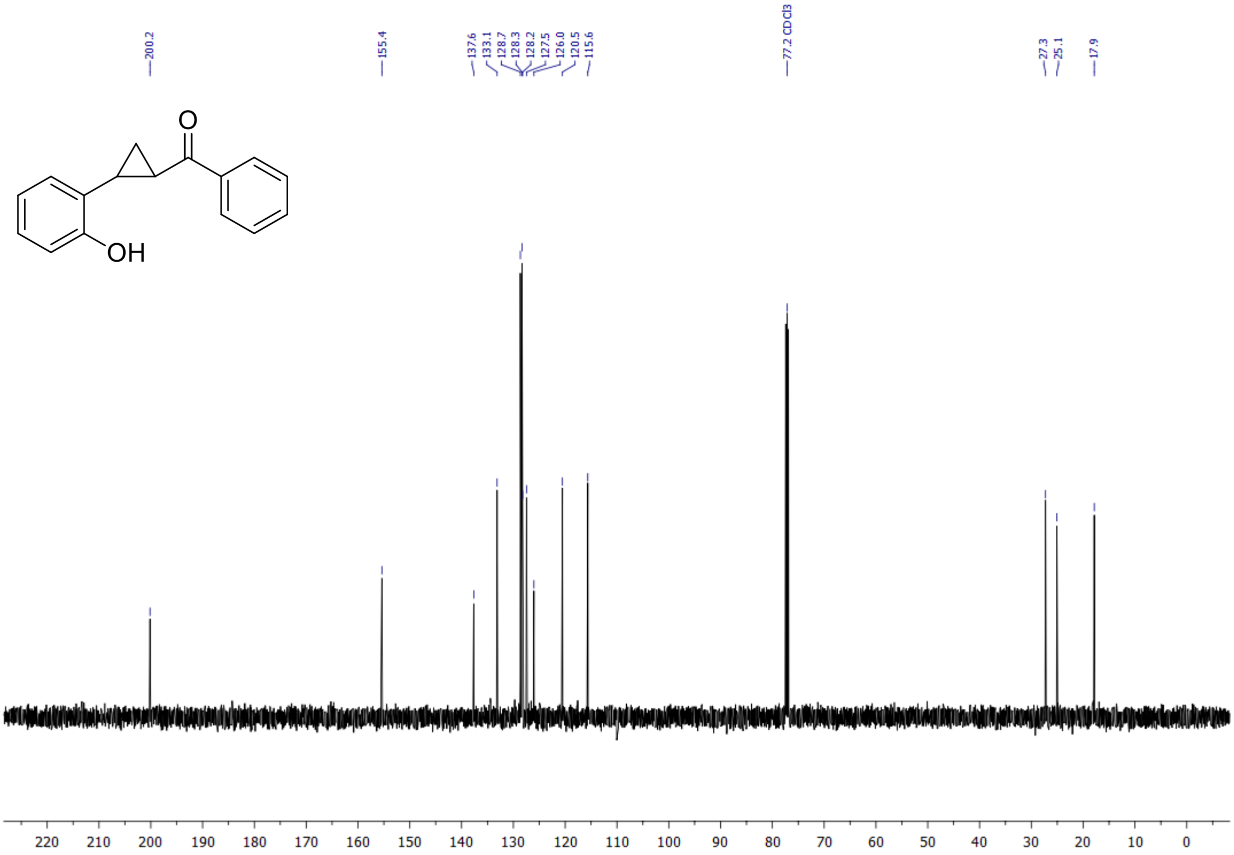

[(1RS,2RS)-2-(2-Hydroxyphenyl)cyclopropyl](4-methoxyphenyl)methanone (**2b**)

^{1}H NMR (500 MHz, DMSO-*d*$_6$)

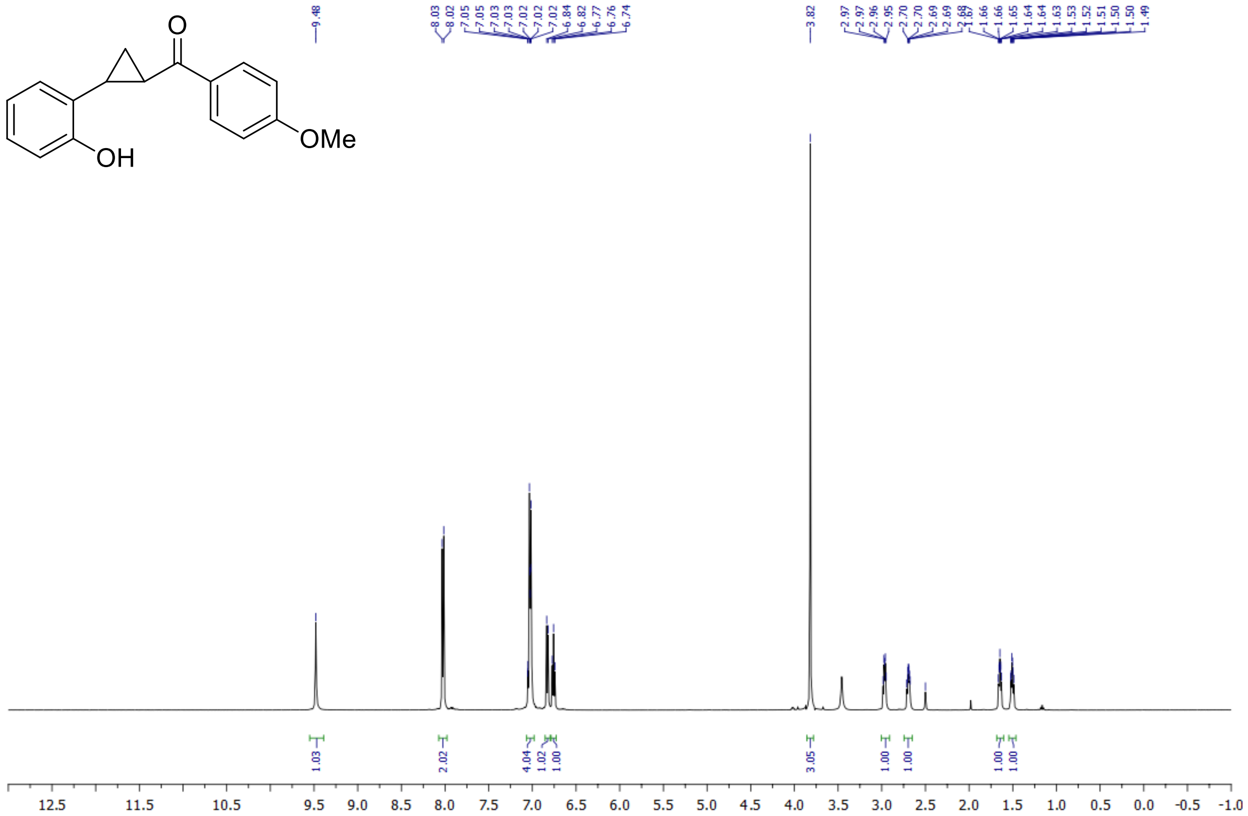

^{13}C NMR (126 MHz, DMSO-*d*$_6$)

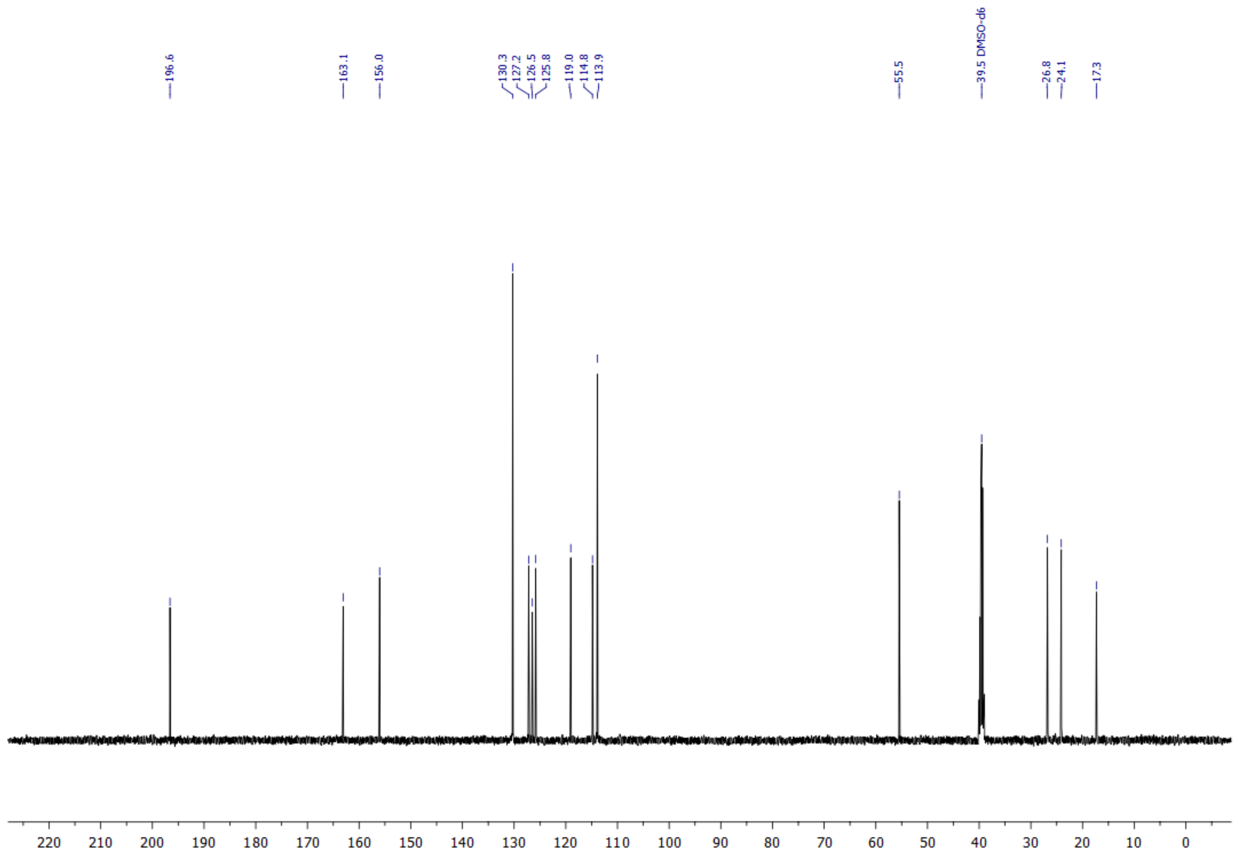

[(1RS,2RS)-2-(2-Hydroxyphenyl)cyclopropyl](thiophen-2-yl)methanone (**2c**)

^{1}H NMR (500 MHz, $CDCl_3$)

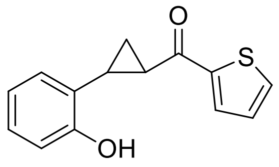

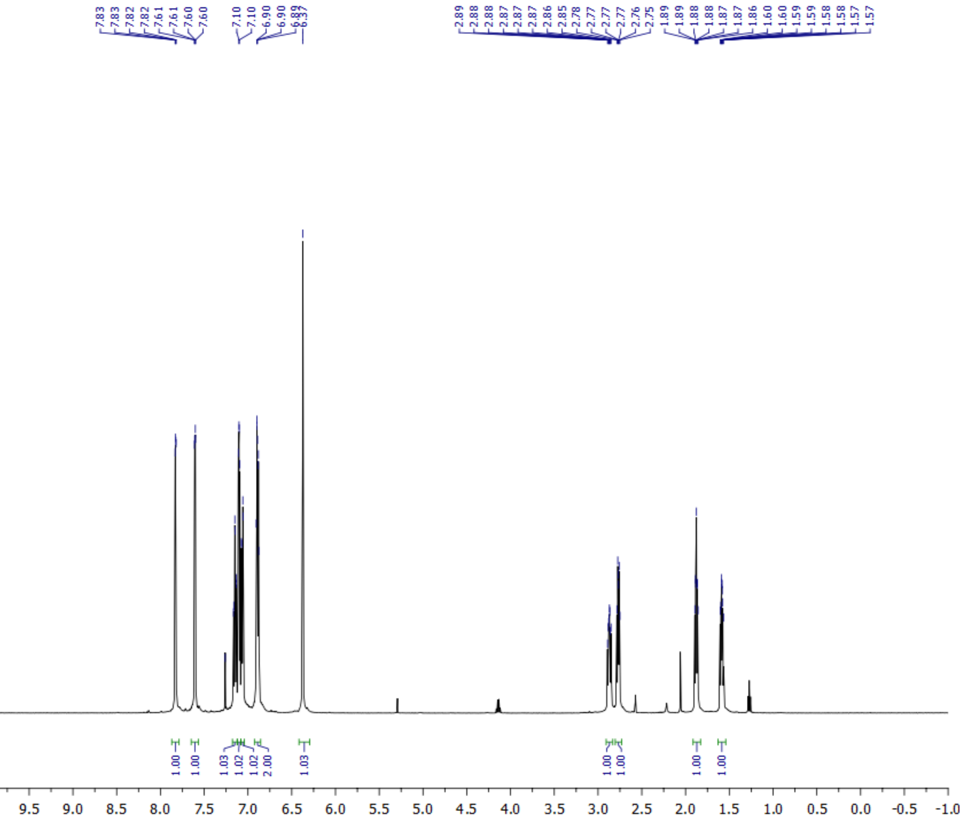

^{13}C NMR (126 MHz, $CDCl_3$)

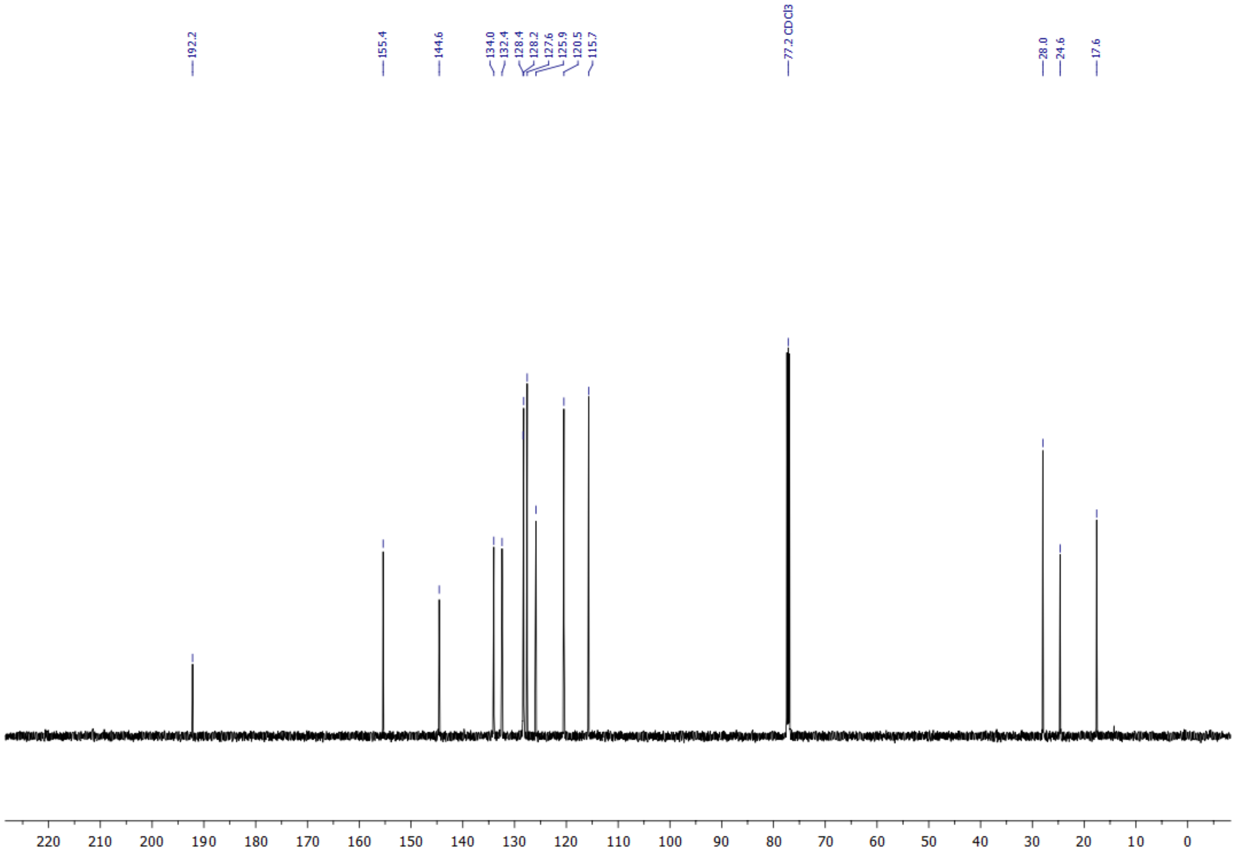

[(1RS,2RS)-2-(5-Fluoro-2-hydroxyphenyl)cyclopropyl](4-methoxyphenyl)methanone (**2d**)

^{1}H NMR (500 MHz, DMSO-d_6)

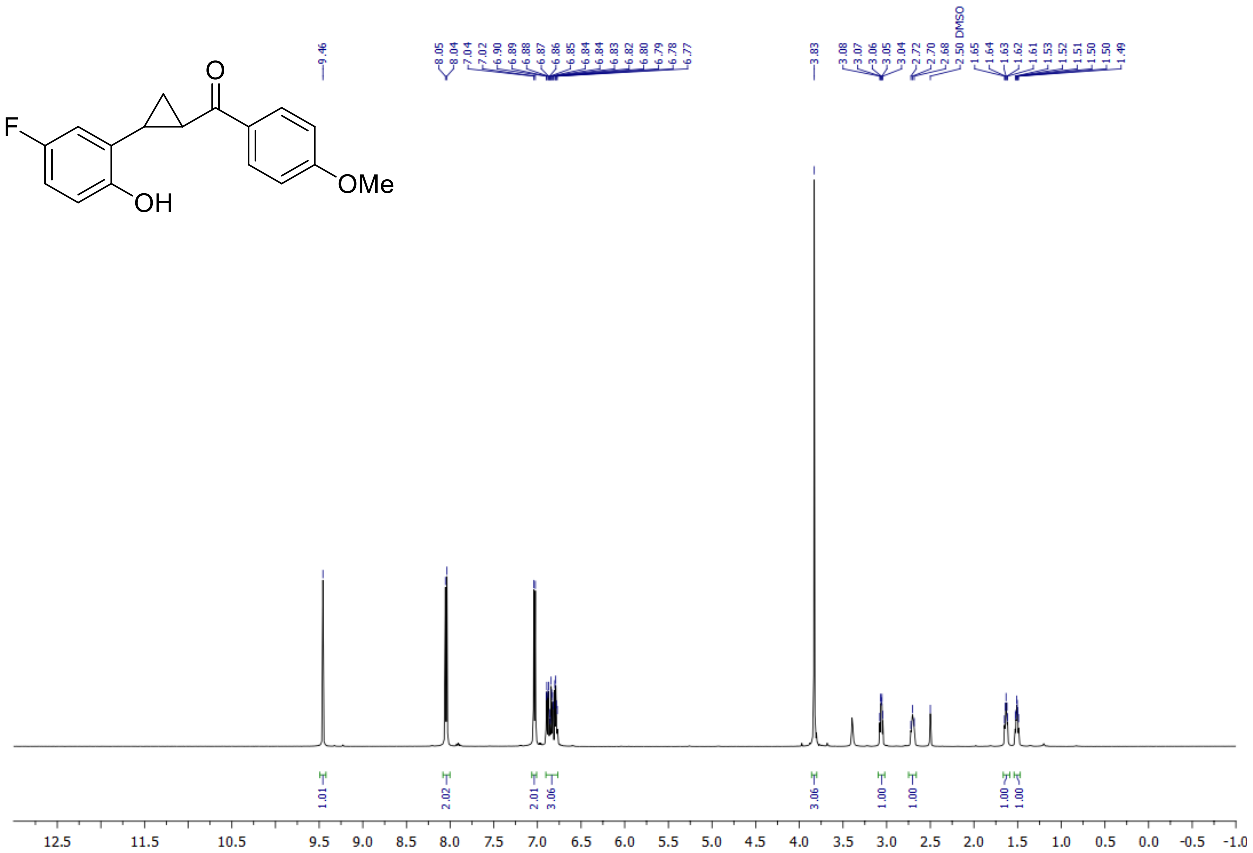

^{13}C NMR (126 MHz, DMSO-d_6)

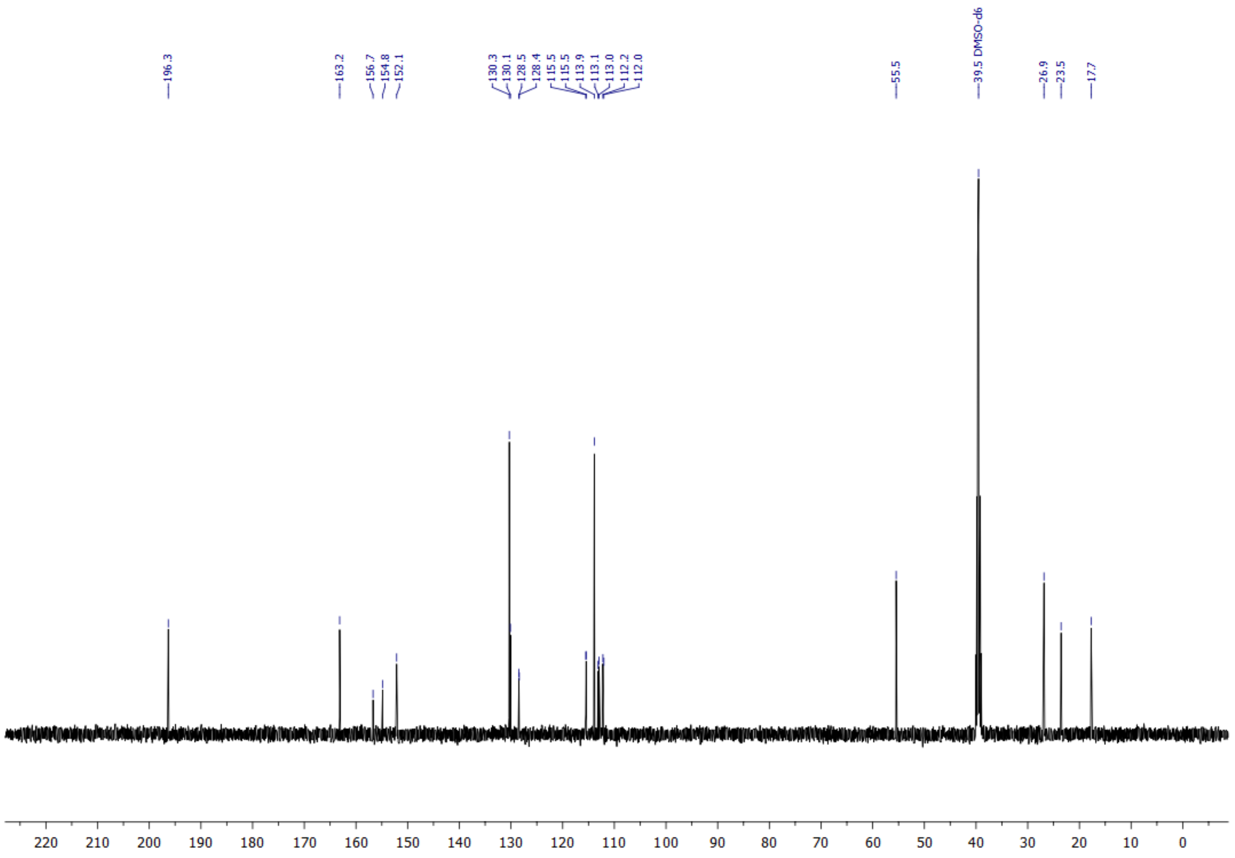

[(1RS,2RS)-2-(5-Fluoro-2-hydroxyphenyl)cyclopropyl](4-methoxyphenyl)methanone (**2d**)
^{19}F NMR (471 MHz, DMSO-d_6)

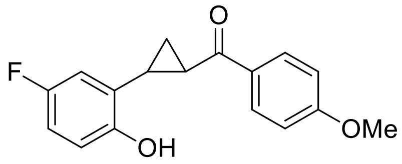

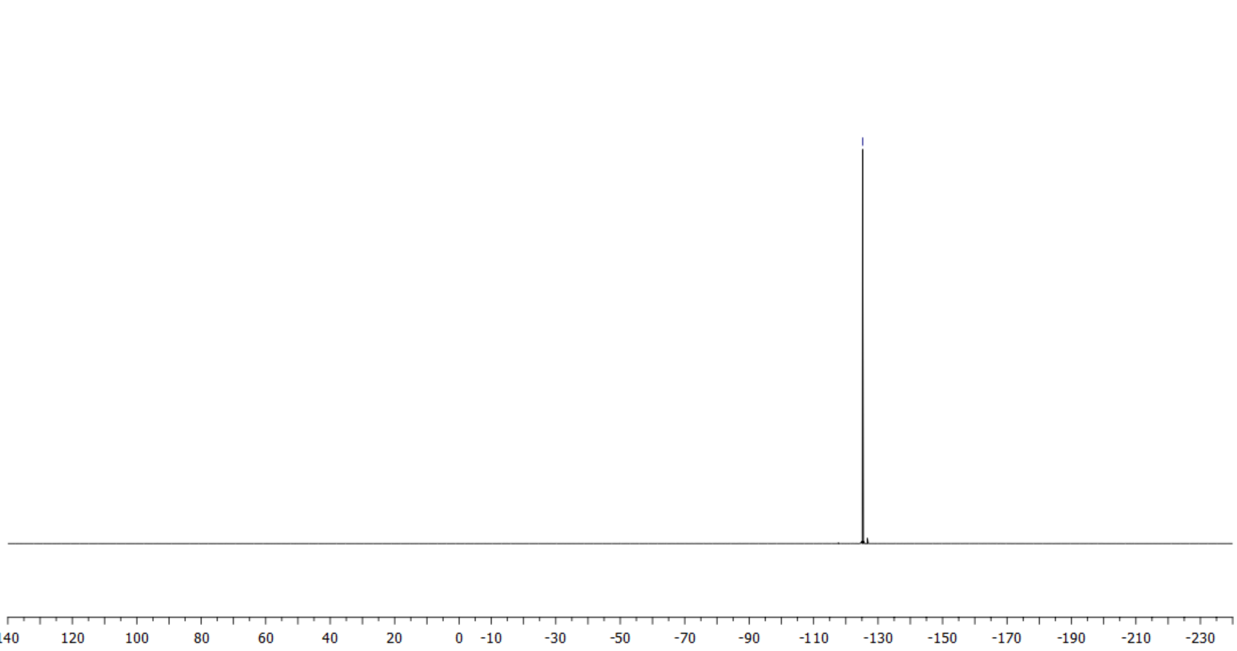

[(1RS,2RS)-2-(5-Bromo-2-hydroxyphenyl)cyclopropyl](4-methoxyphenyl)methanone (**2e**)

^{1}H NMR (500 MHz, CDCl$_3$)

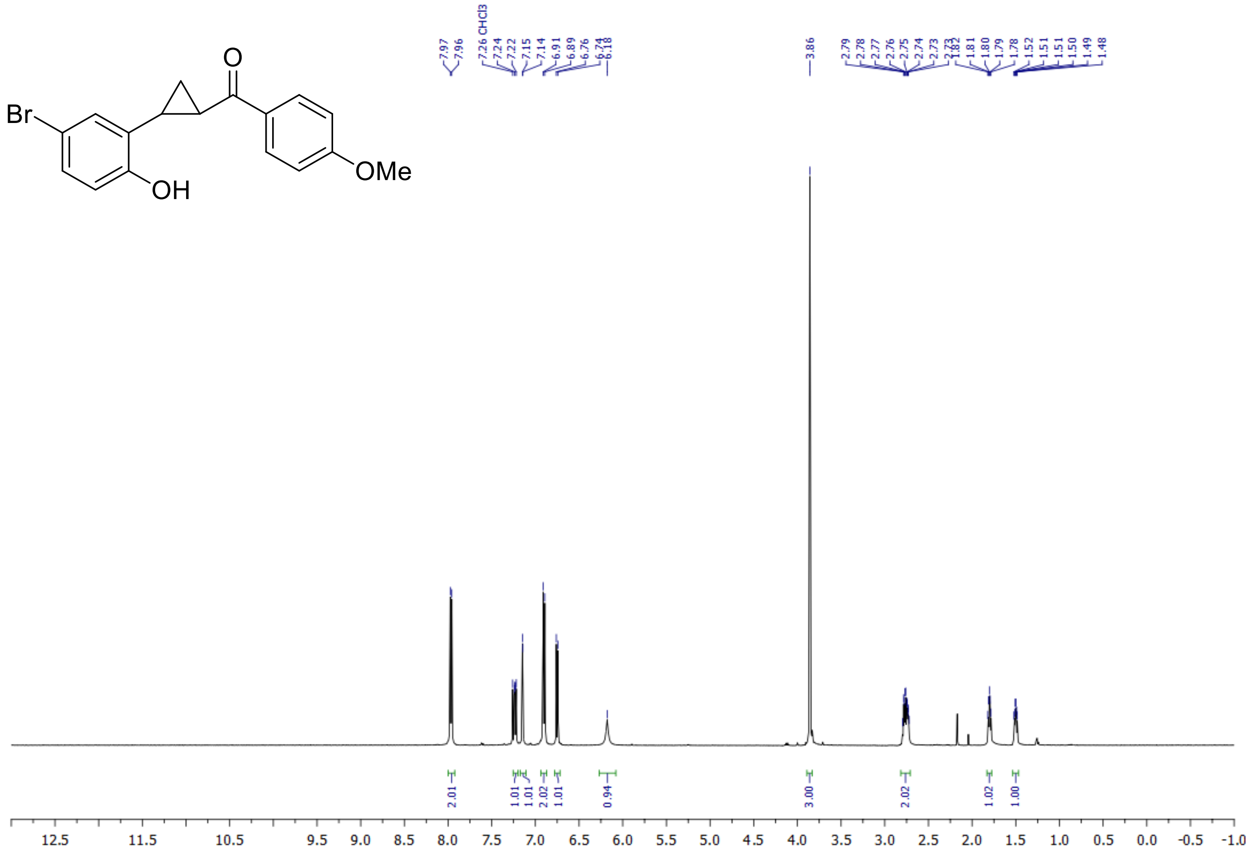

^{13}C NMR (126 MHz, CDCl$_3$)

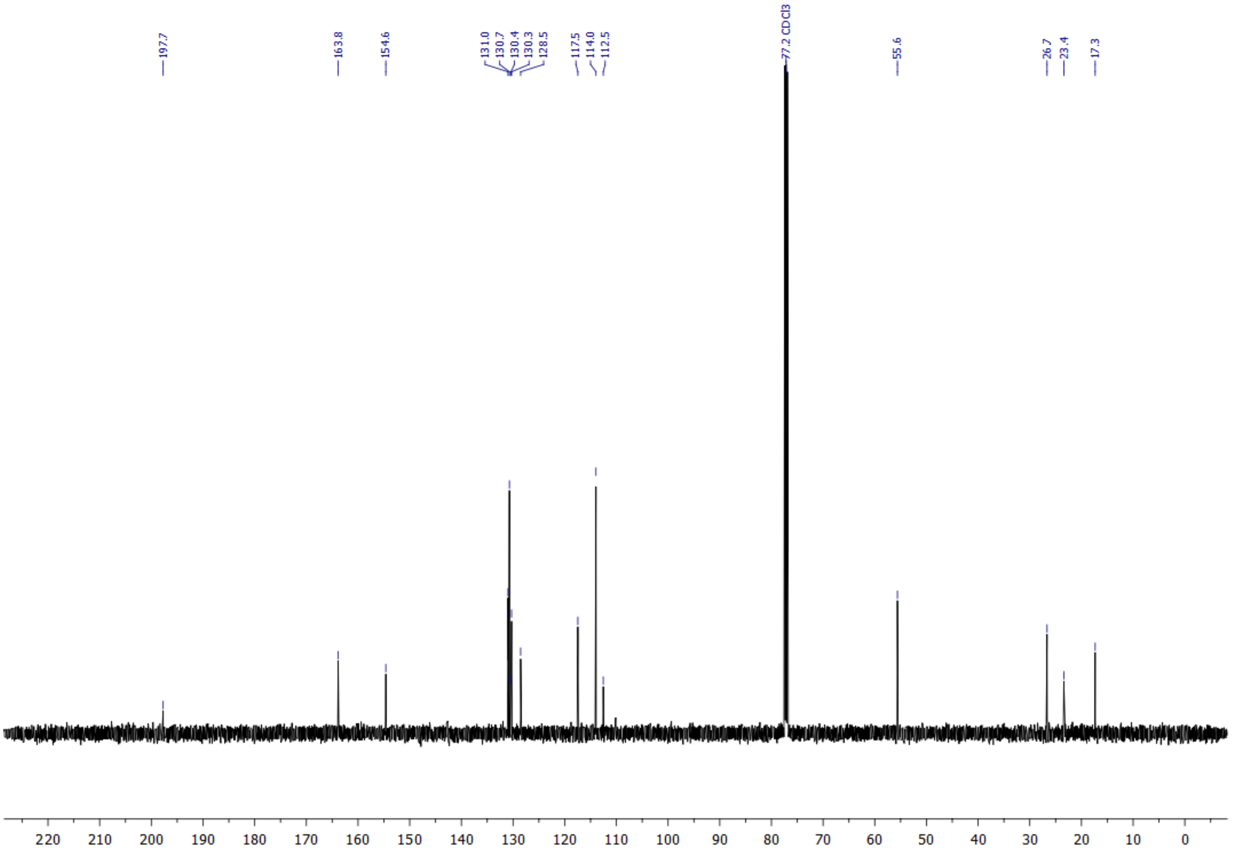

[(1RS,2RS)-2-(2-Hydroxy-5-nitrophenyl)cyclopropyl](4-methoxyphenyl)methanone (**2f**)

^{1}H NMR (500 MHz, DMSO-*d*$_6$)

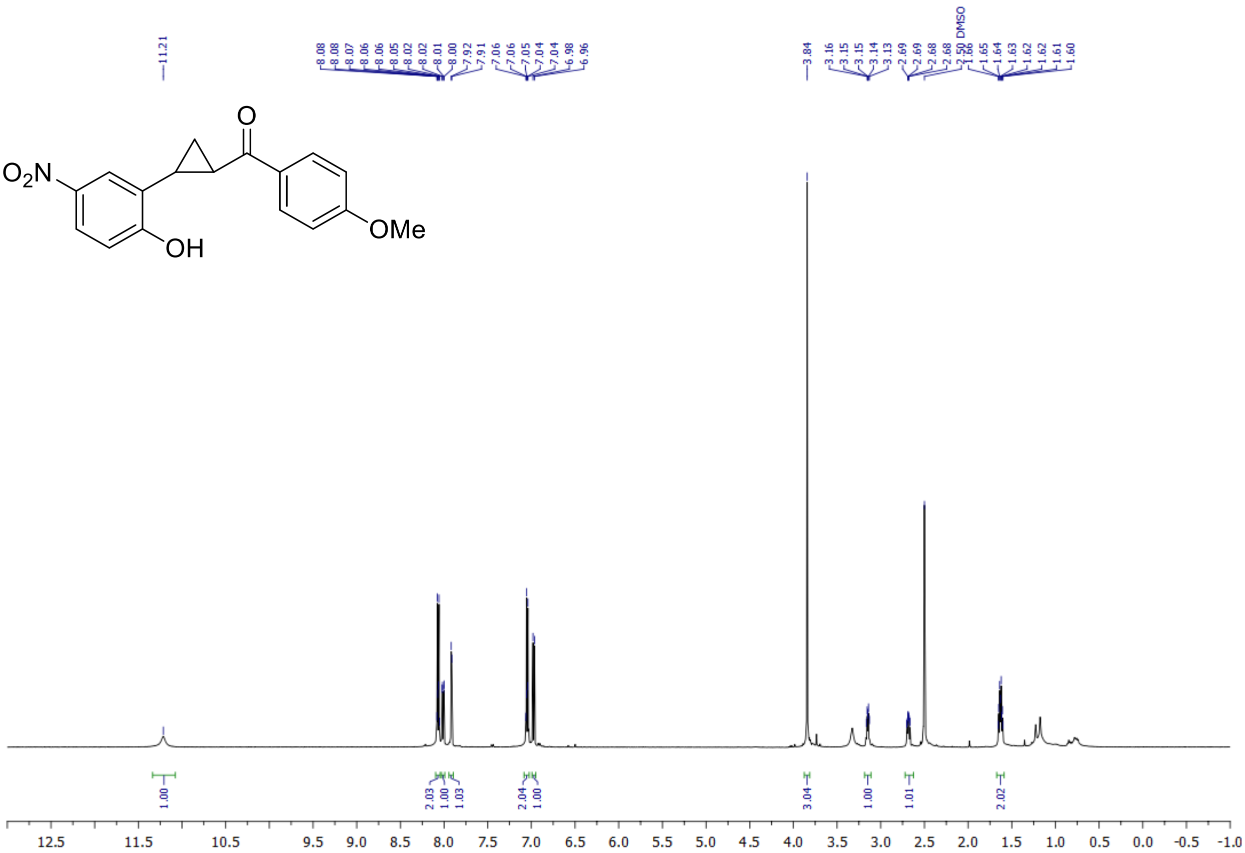

^{13}C NMR (126 MHz, DMSO-*d*$_6$)

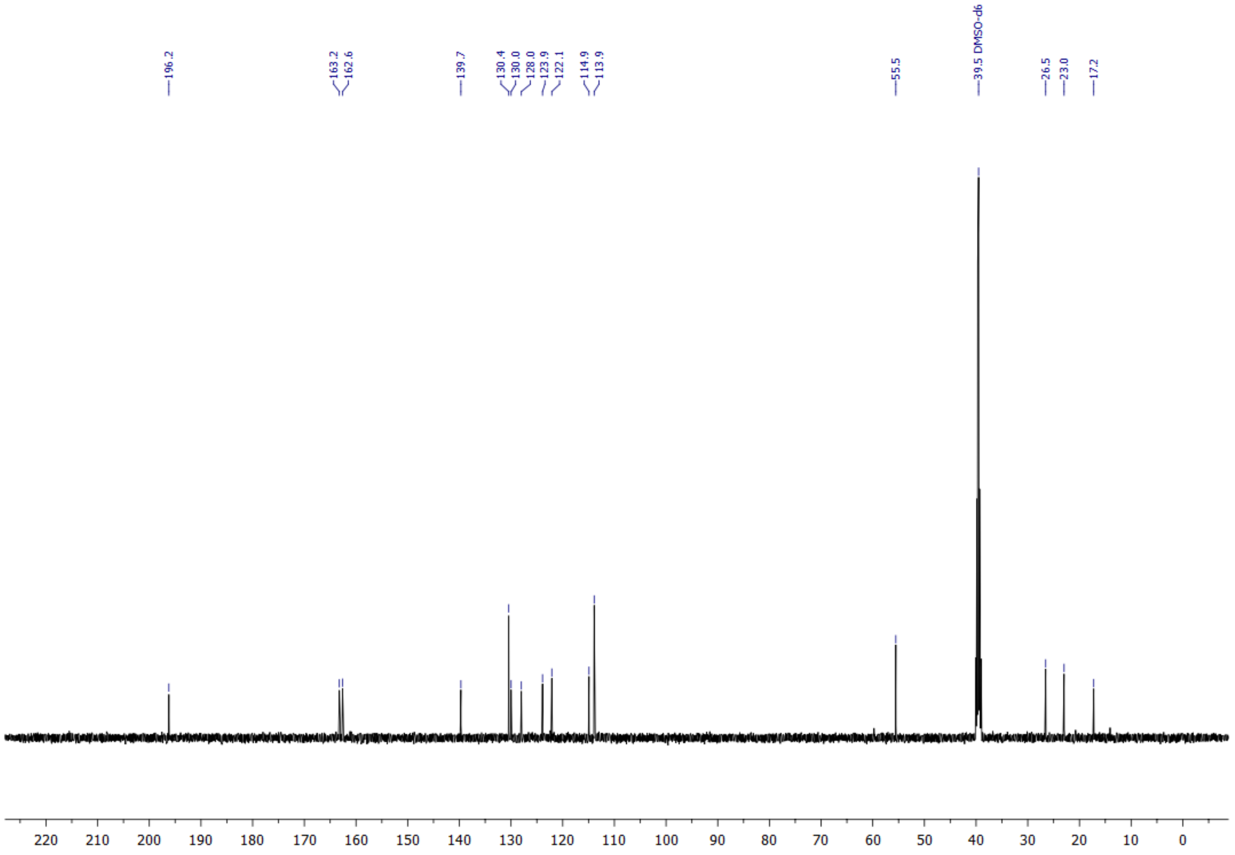

[(1RS,2RS)-2-(5-Fluoro-2-hydroxyphenyl)cyclopropyl](phenyl)methanone (**2g**)

^{1}H NMR (500 MHz, $CDCl_3$)

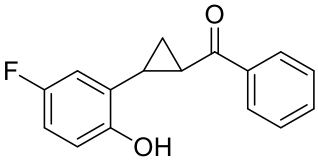

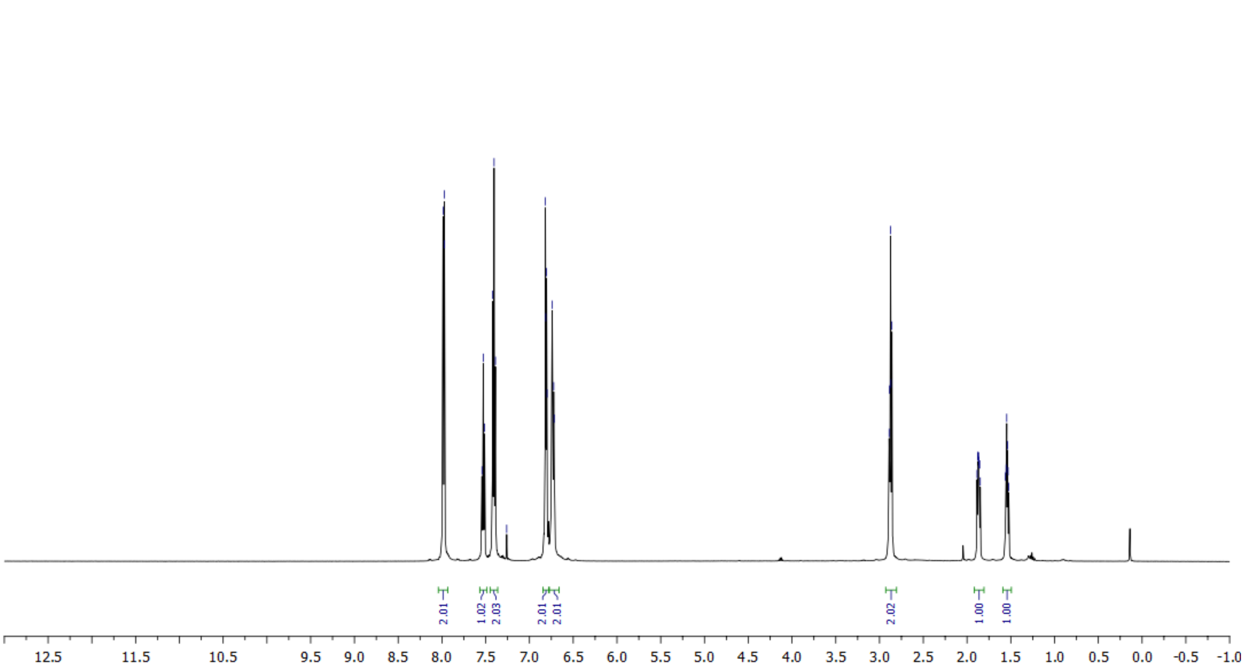

^{13}C NMR (126 MHz, $CDCl_3$)

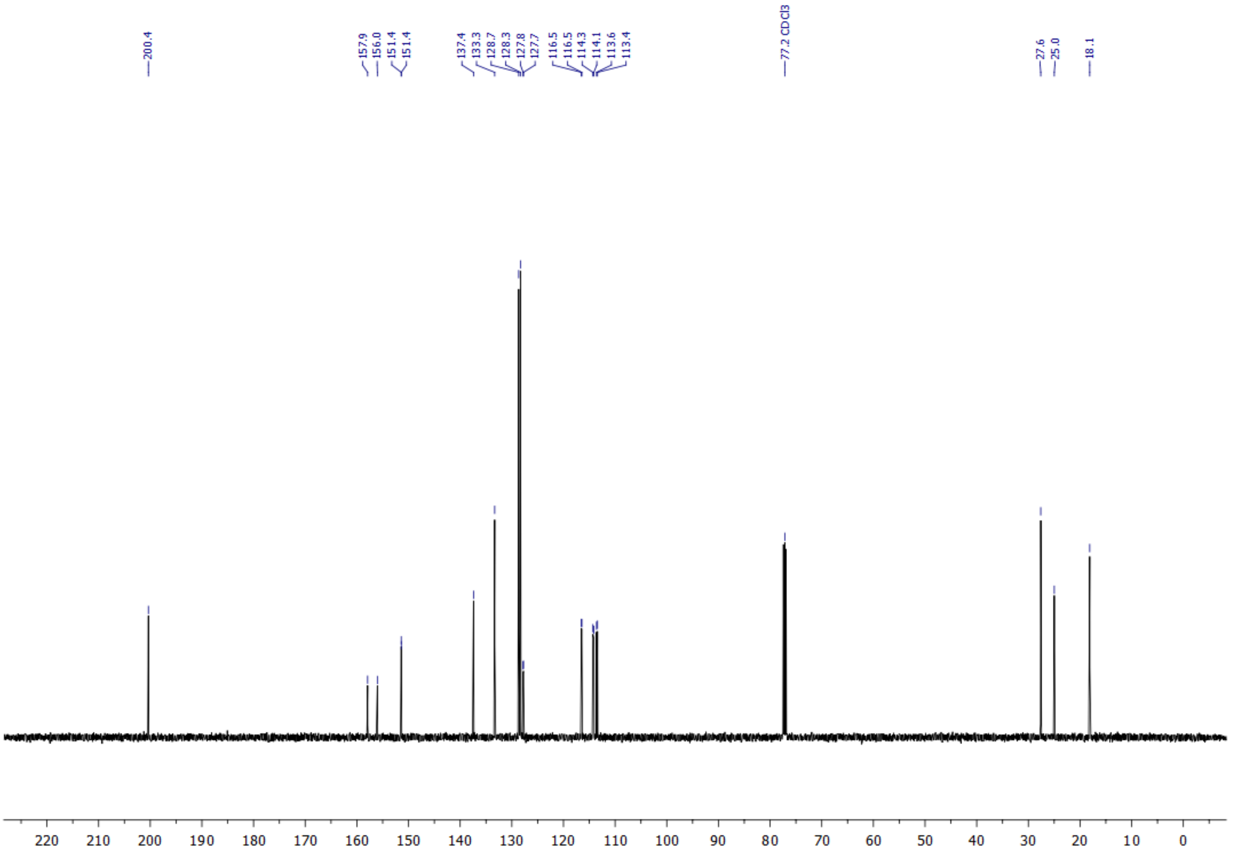

[(1RS,2RS)-2-(5-Fluoro-2-hydroxyphenyl)cyclopropyl](phenyl)methanone (**2g**)

^{19}F NMR (471 MHz, $CDCl_3$)

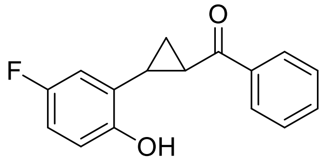

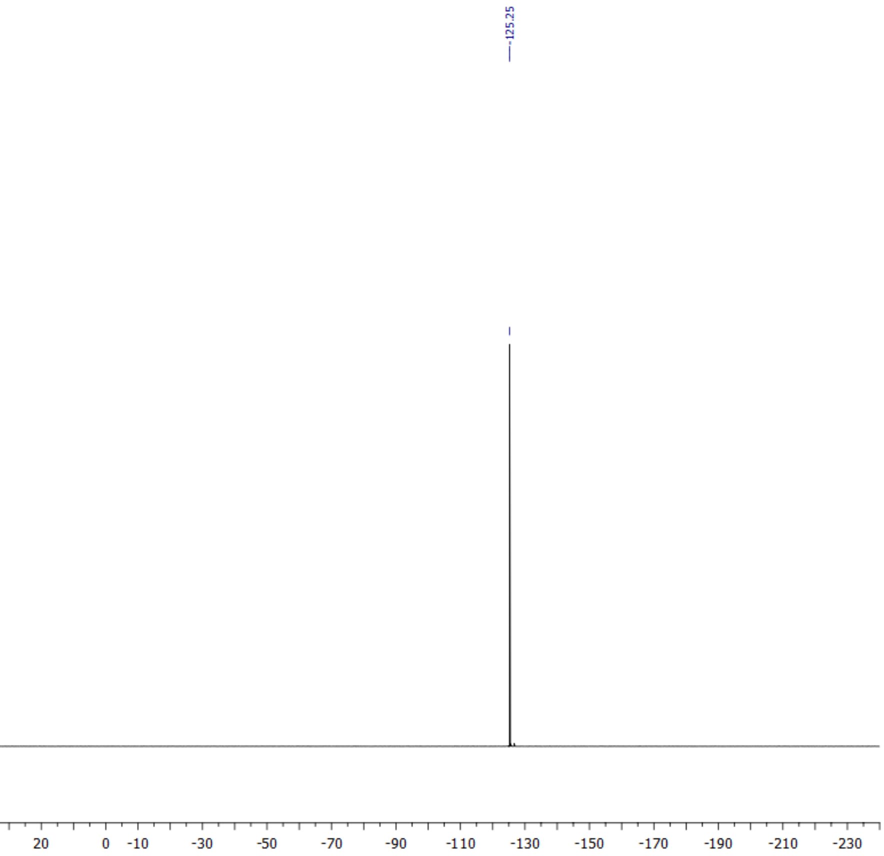

[(1RS,2RS)-2-(5-Bromo-2-hydroxyphenyl)cyclopropyl](phenyl)methanone (**2h**)

H NMR (500 MHz, $CDCl_3$)

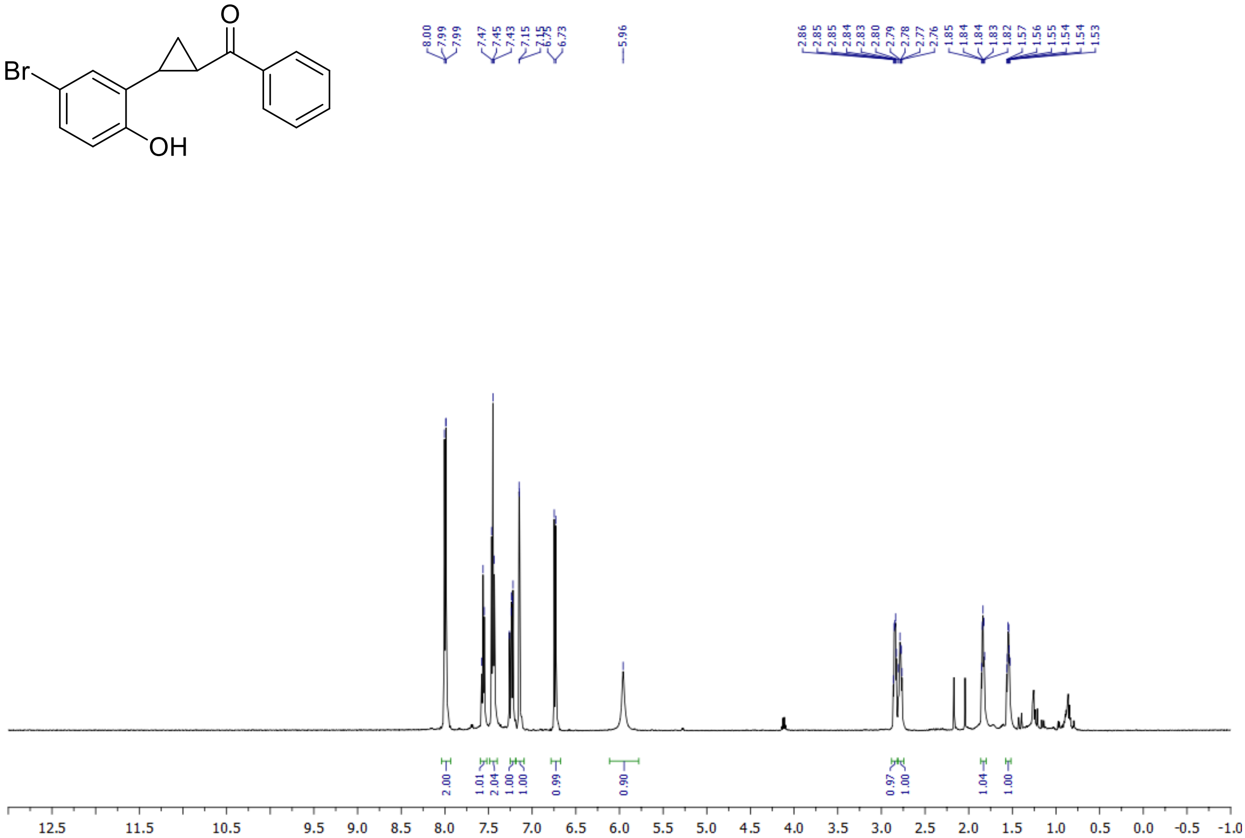

^{13}C NMR (126 MHz, $CDCl_3$)

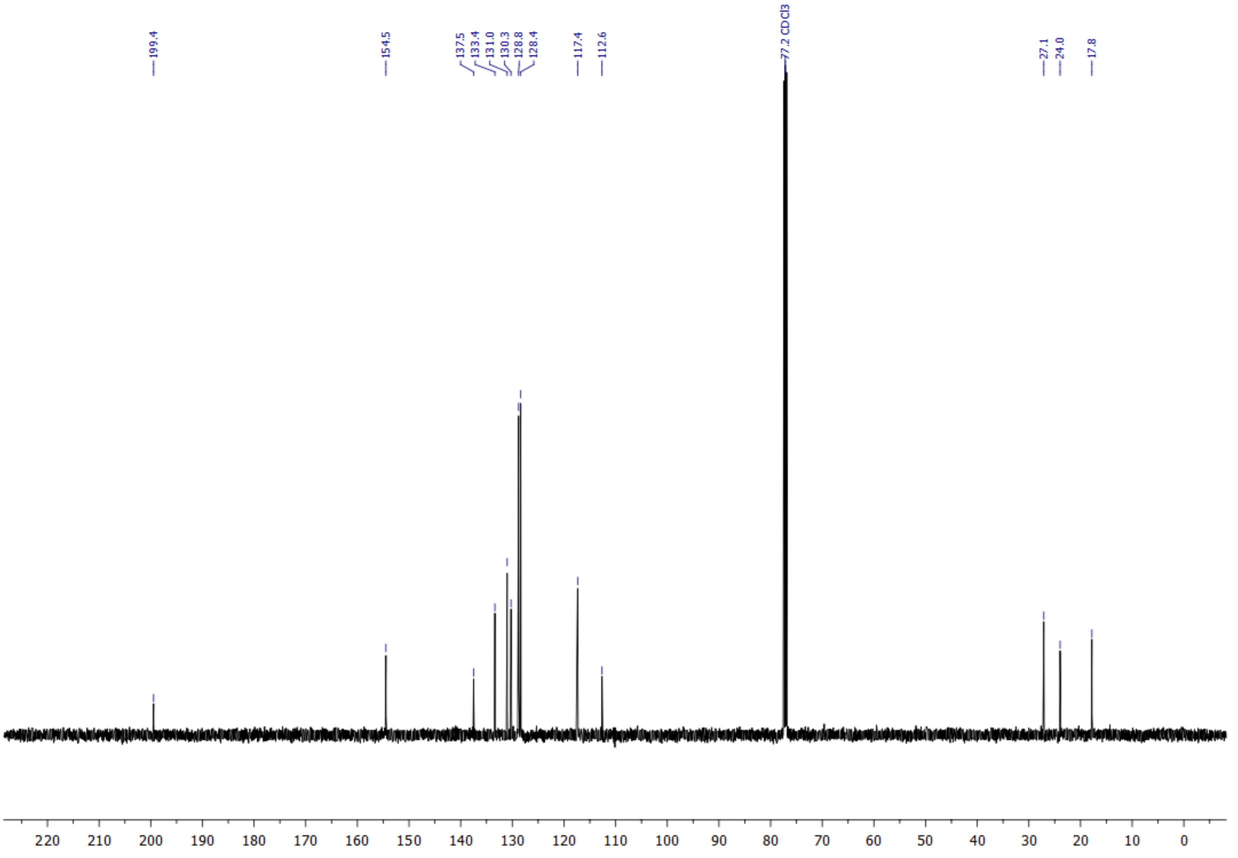

[(1RS,2RS)-2-(2-Hydroxy-5-nitrophenyl)cyclopropyl](phenyl)methanone (**2i**)

^{1}H NMR (400 MHz, DMSO-d_6)

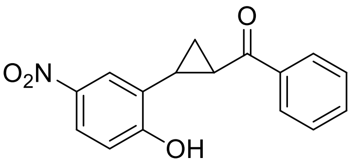

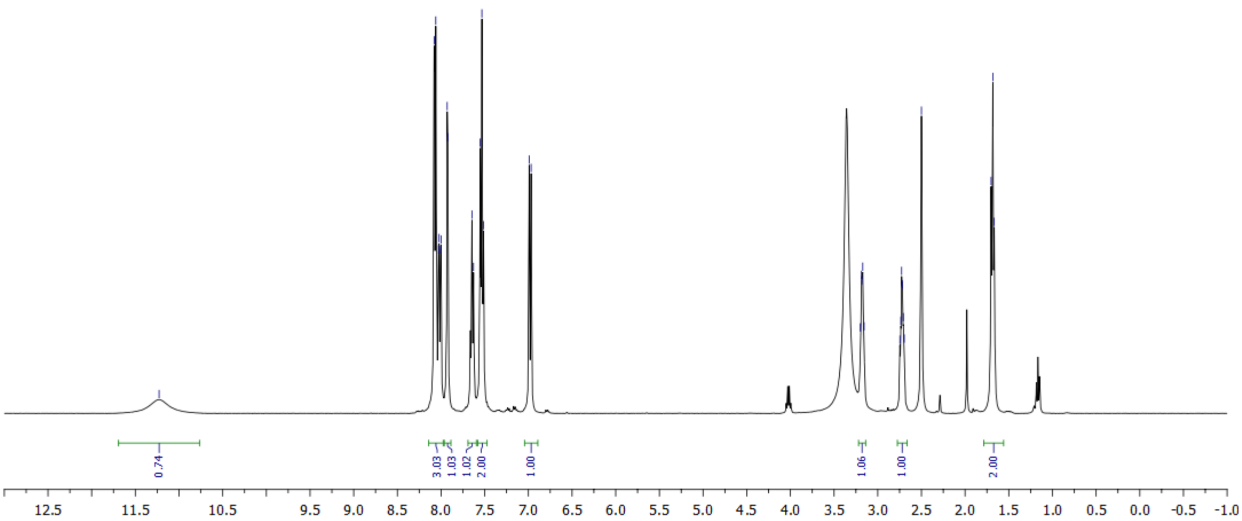

^{13}C NMR (101 MHz, DMSO-d_6)

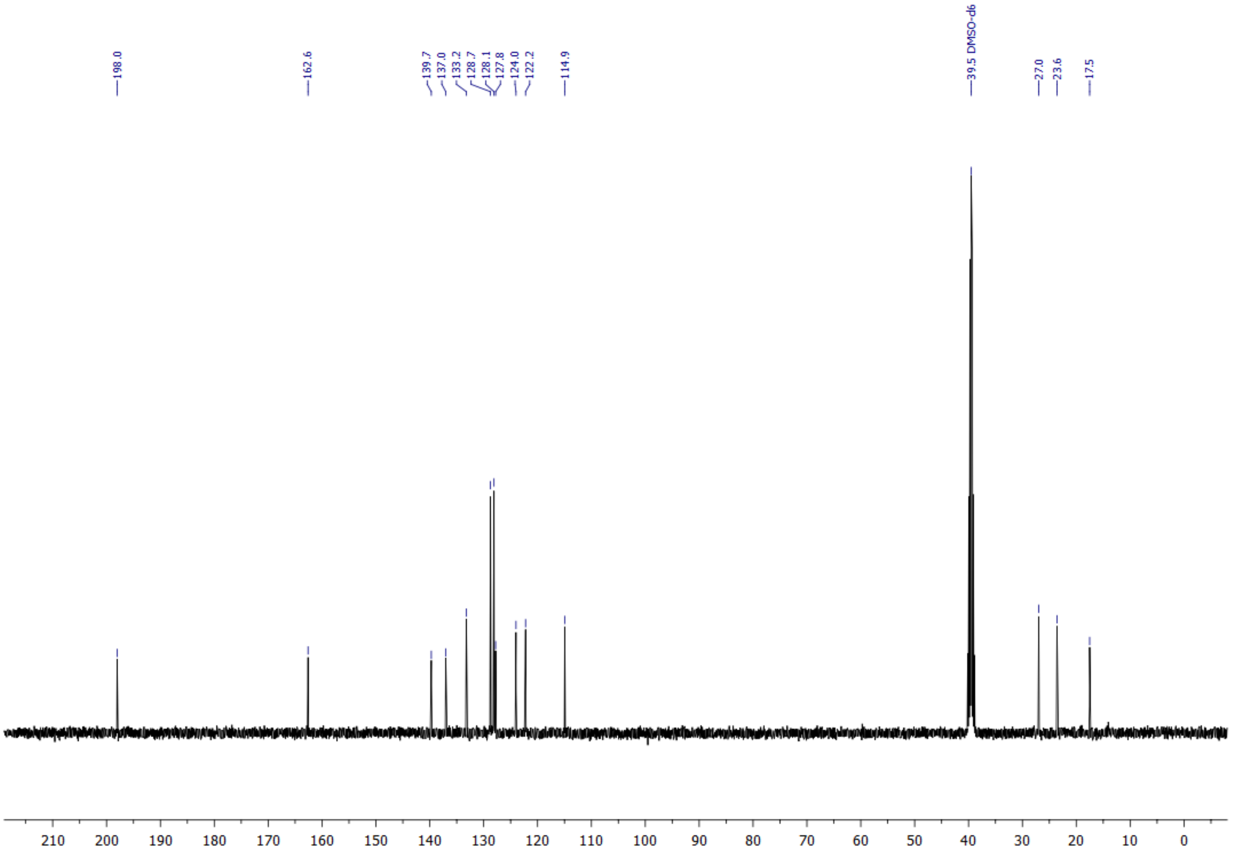

[(1RS,2RS)-2-(5-Fluoro-2-hydroxyphenyl)cyclopropyl](thiophen-2-yl)methanone (**2j**)

^{1}H NMR (500 MHz, $CDCl_3$)

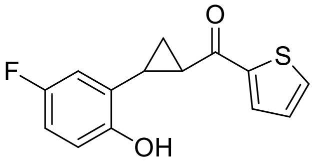

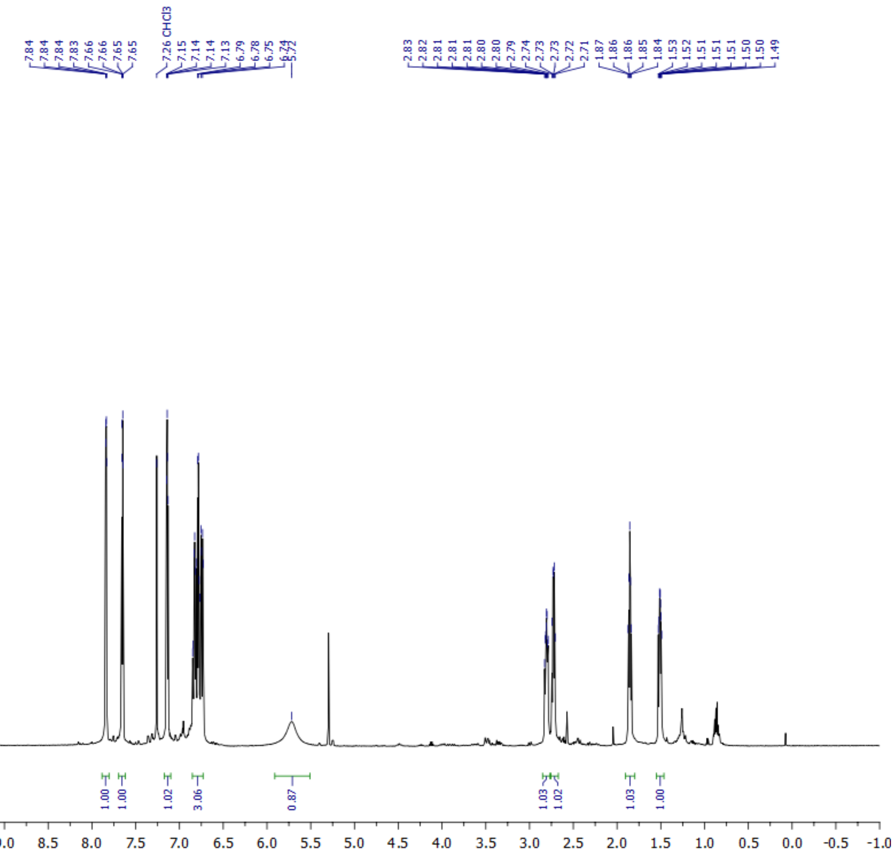

^{13}C NMR (126 MHz, $CDCl_3$)

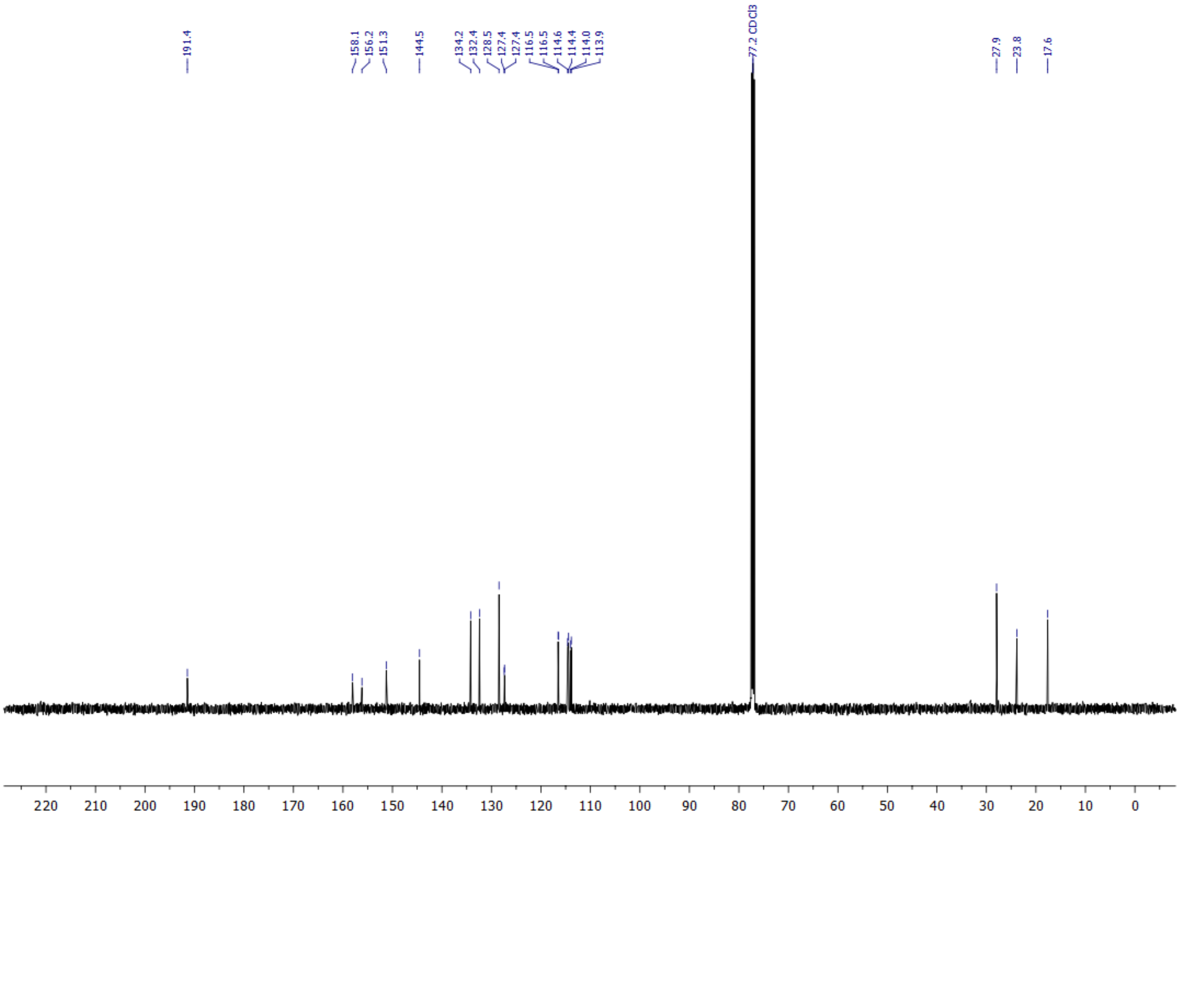

[(1RS,2RS)-2-(5-Fluoro-2-hydroxyphenyl)cyclopropyl](thiophen-2-yl)methanone (**2j**)

^{19}F NMR (471 MHz, $CDCl_3$)

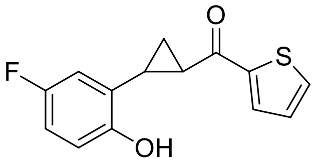

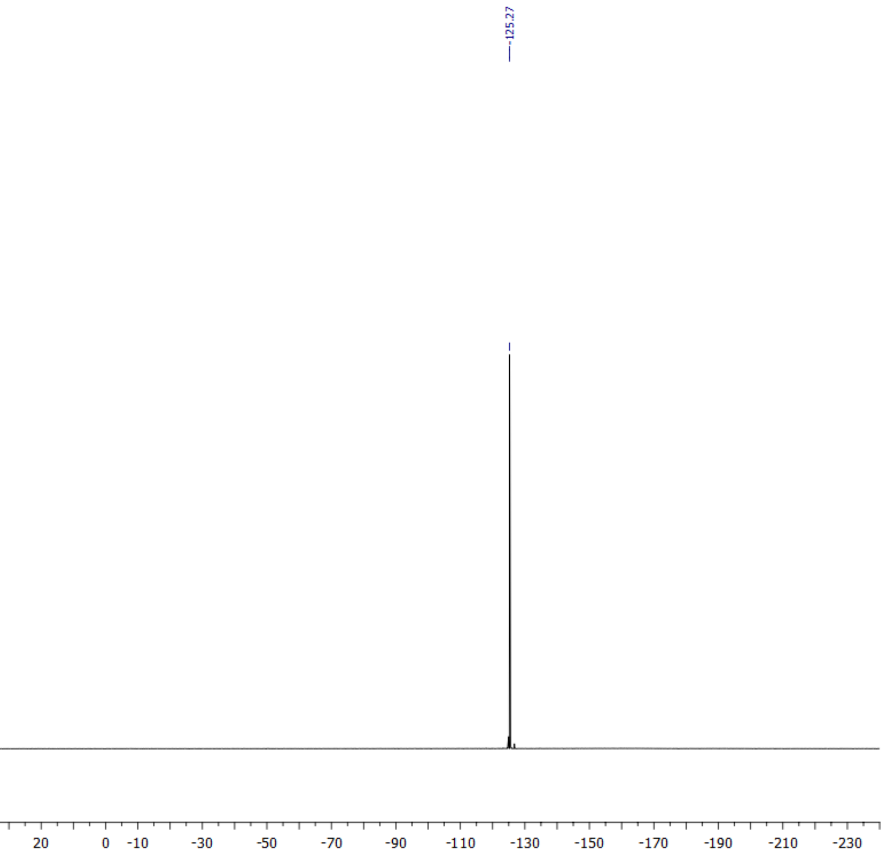

[(1RS,2RS)-2-(5-Chloro-2-hydroxyphenyl)cyclopropyl](thiophen-2-yl)methanone (**2k**)

^{1}H NMR (500 MHz, $CDCl_3$)

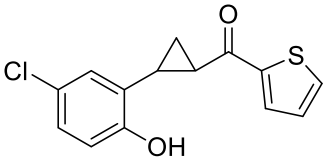

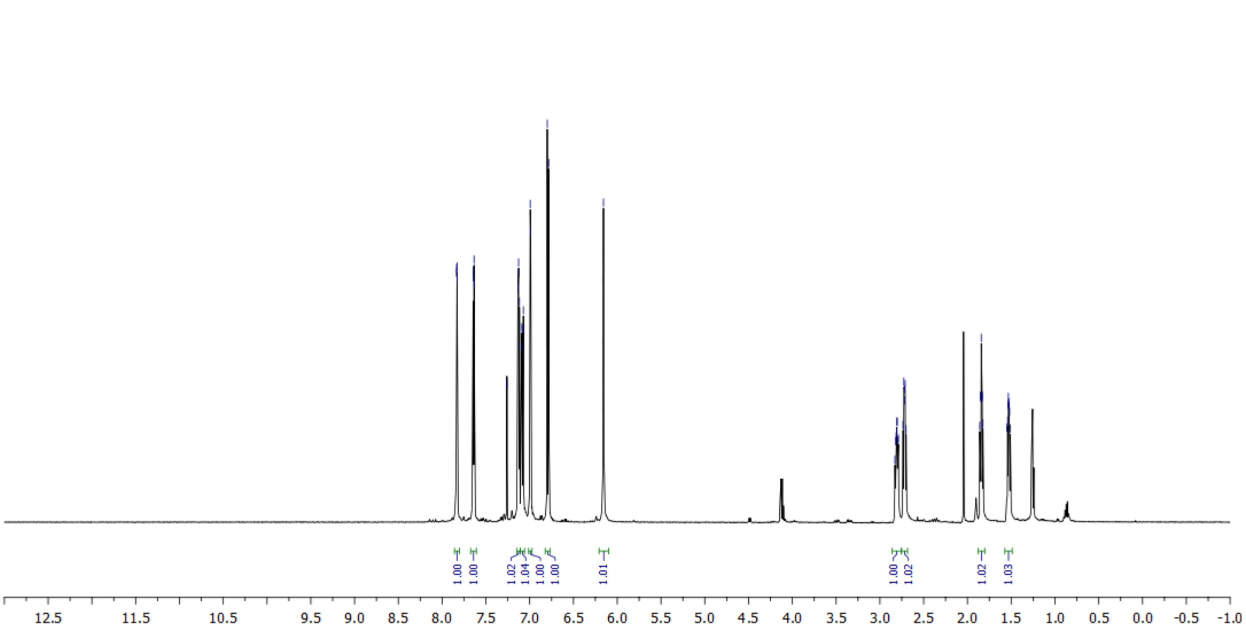

^{1}H NMR (500 MHz, DMSO-d_6)

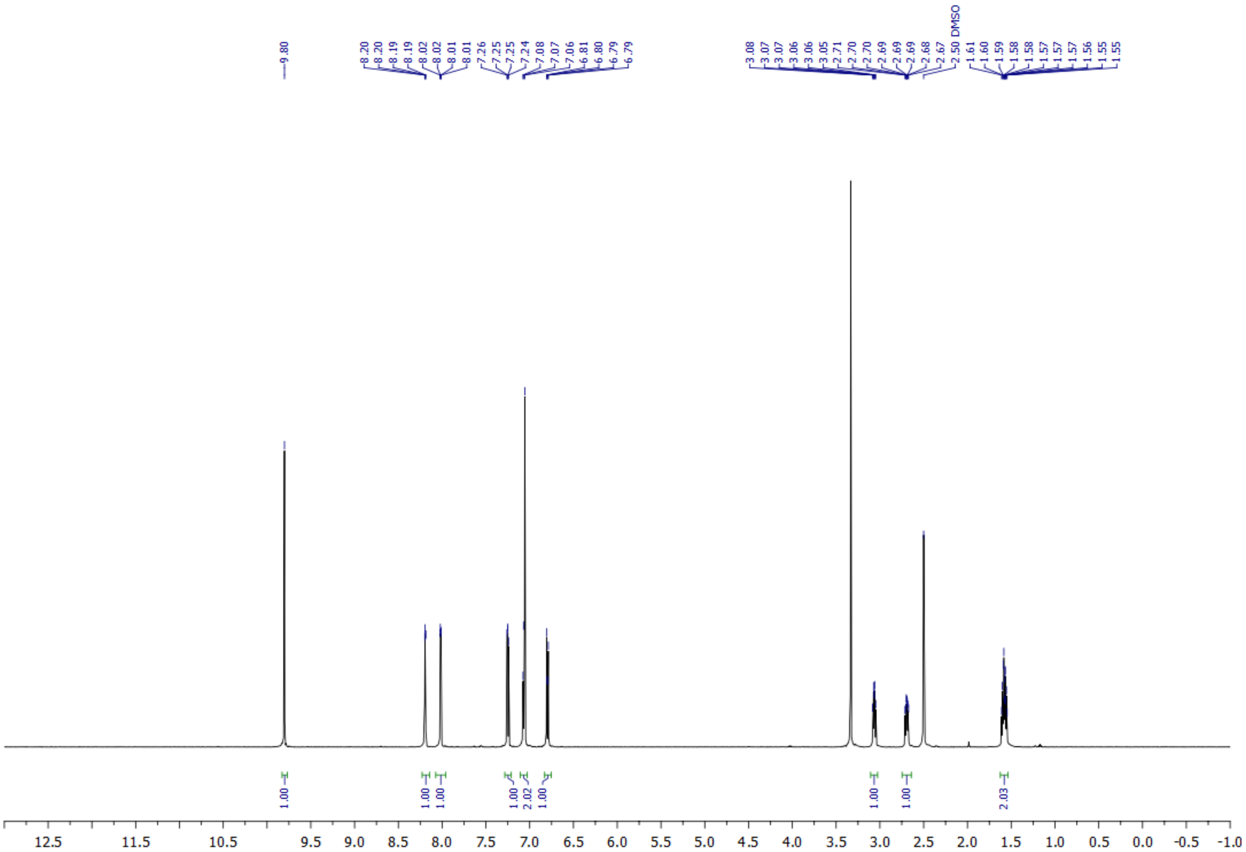

^{13}C NMR (126 MHz, $CDCl_3$)

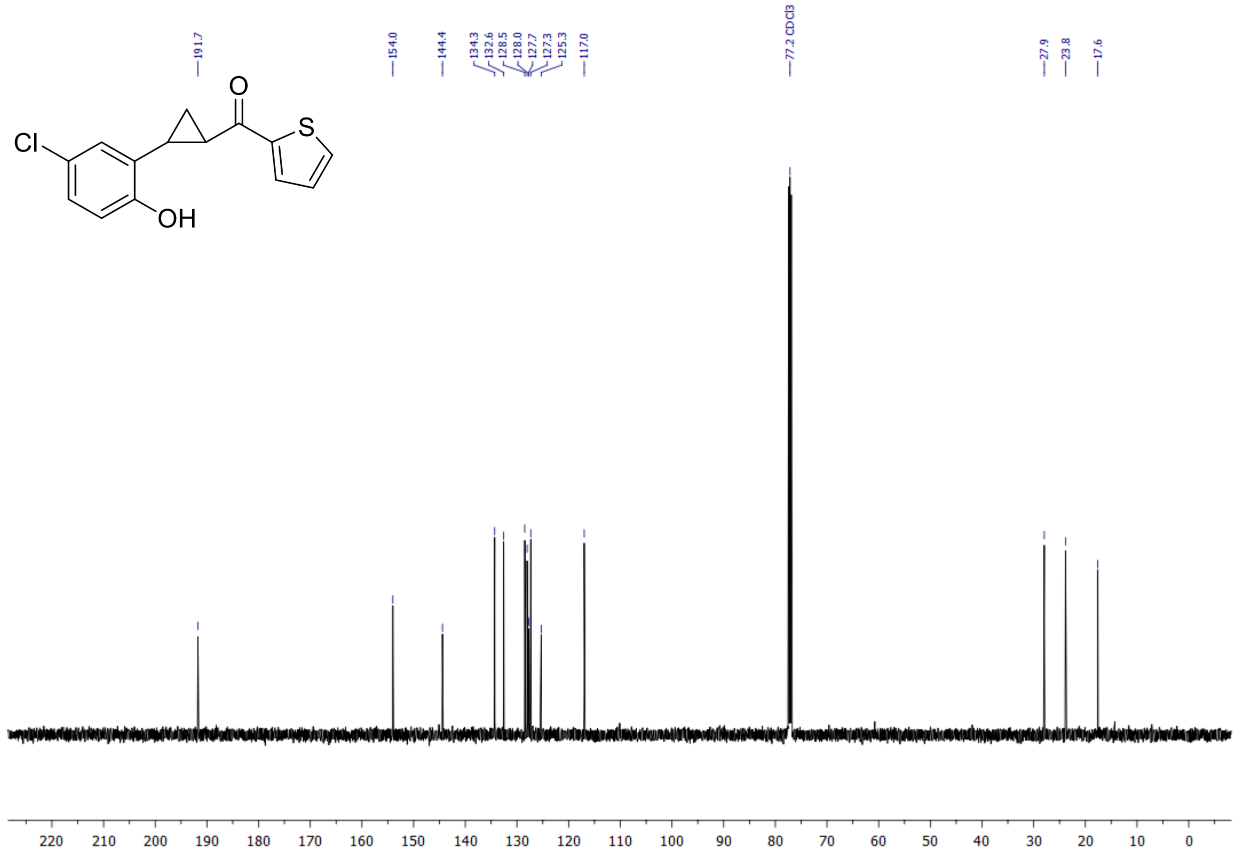

HSQC (^{1}H - ^{13}C)

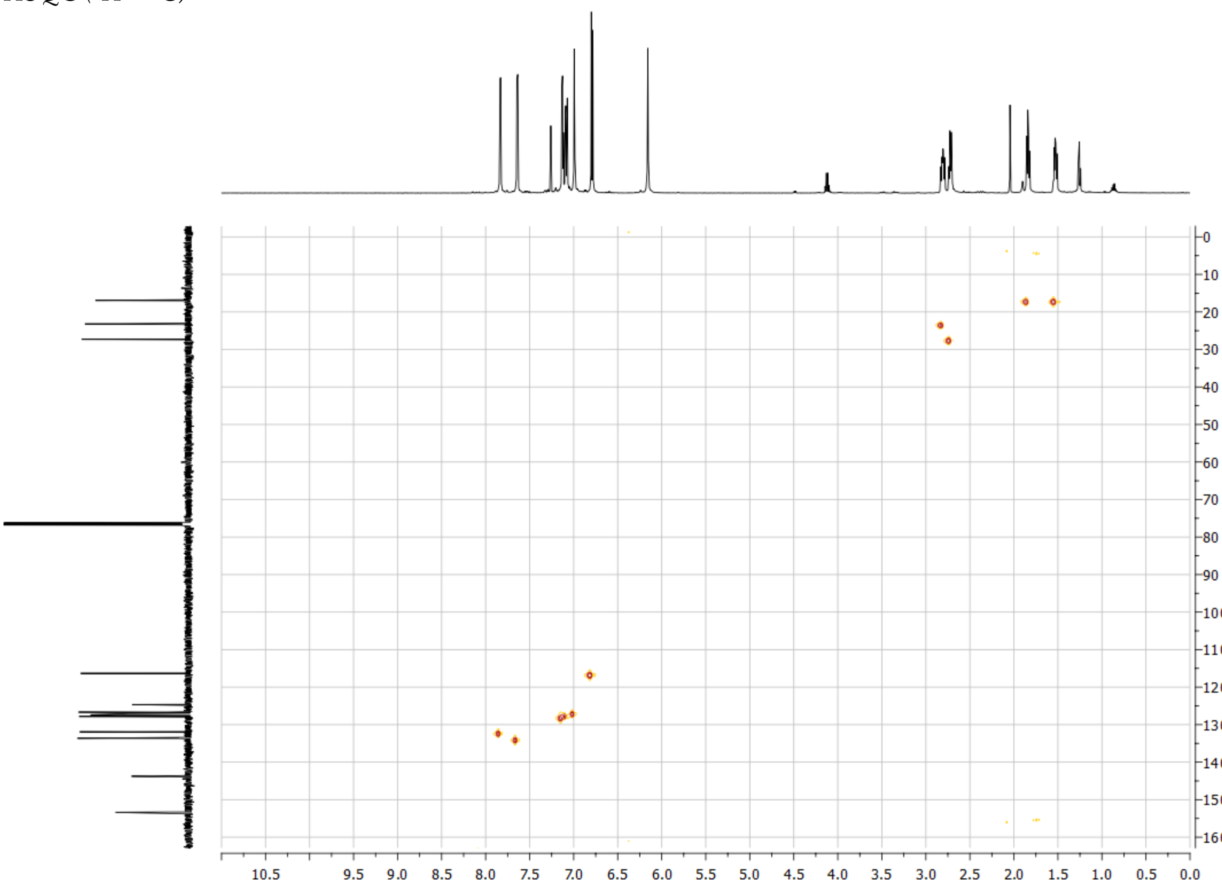

[(1RS,2RS)-2-(5-Bromo-2-hydroxyphenyl)cyclopropyl](thiophen-2-yl)methanone (**21**)

^{1}H NMR (500 MHz, DMSO-d$_6$)

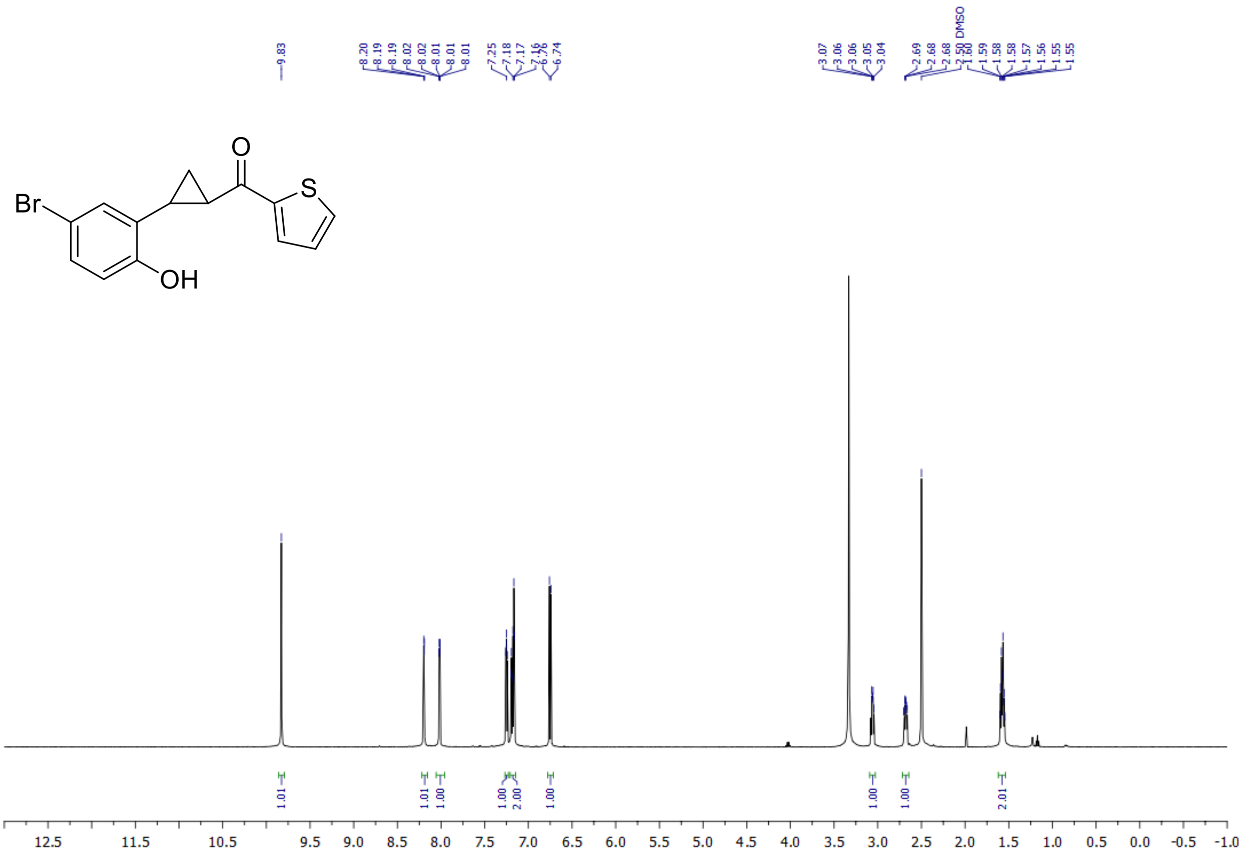

^{13}C NMR (126 MHz, DMSO-d$_6$)

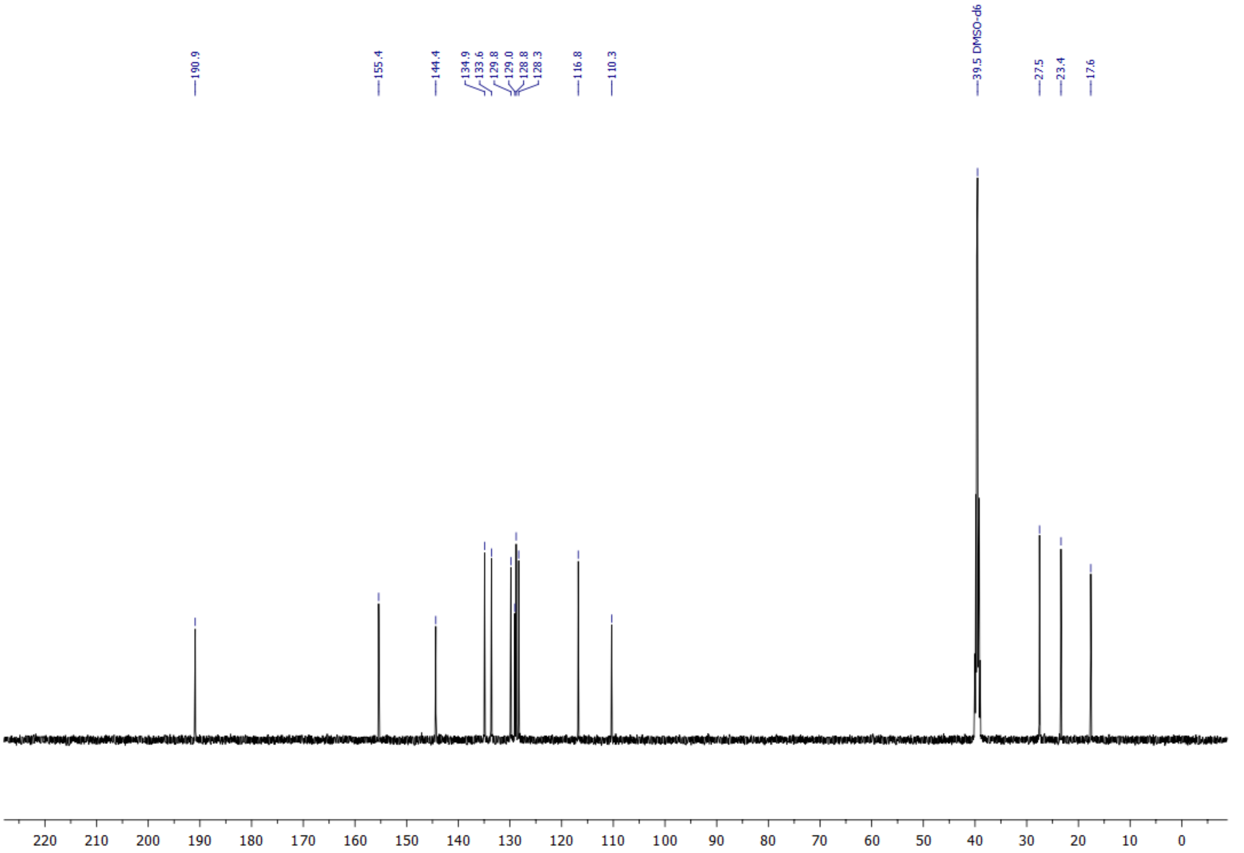

[(1RS,2RS)-2-(2-Hydroxy-5-nitrophenyl)cyclopropyl](thiophen-2-yl)methanone (**2m**)

^{1}H NMR (500 MHz, DMSO-d_6)

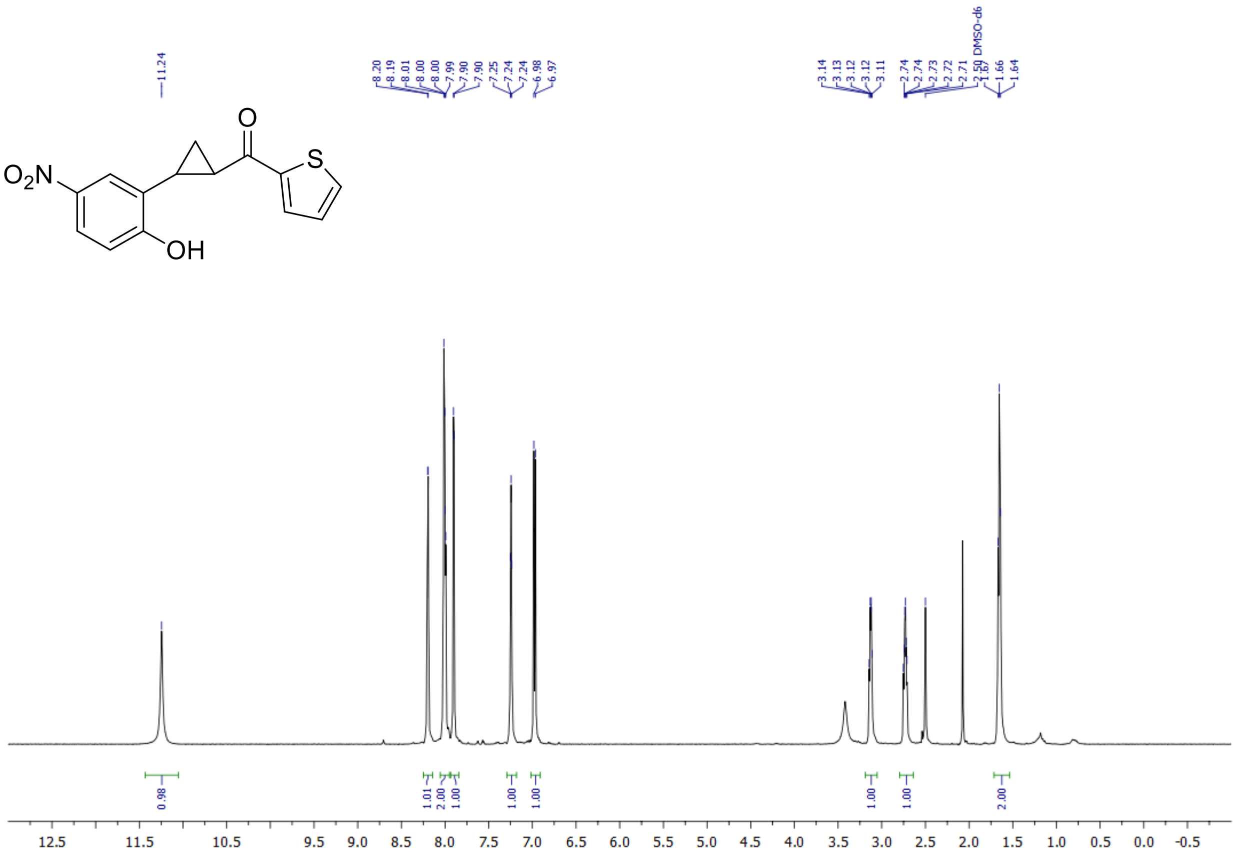

^{13}C NMR (126 MHz, DMSO-d_6)

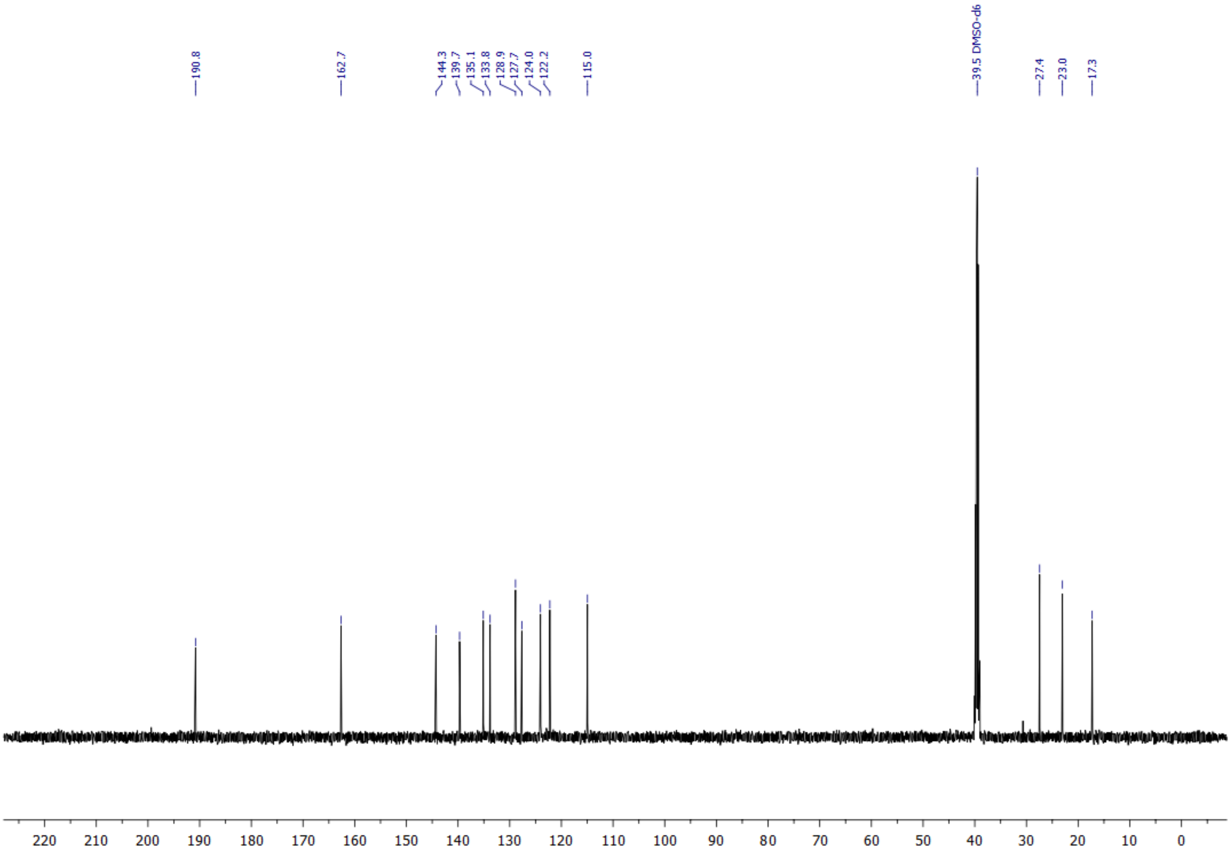

[(1RS,2RS)-2-(2-Hydroxy-3-methoxyphenyl)cyclopropyl](phenyl)methanone (**2n**)

^{1}H NMR (500 MHz, CDCl$_3$)

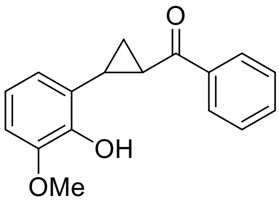

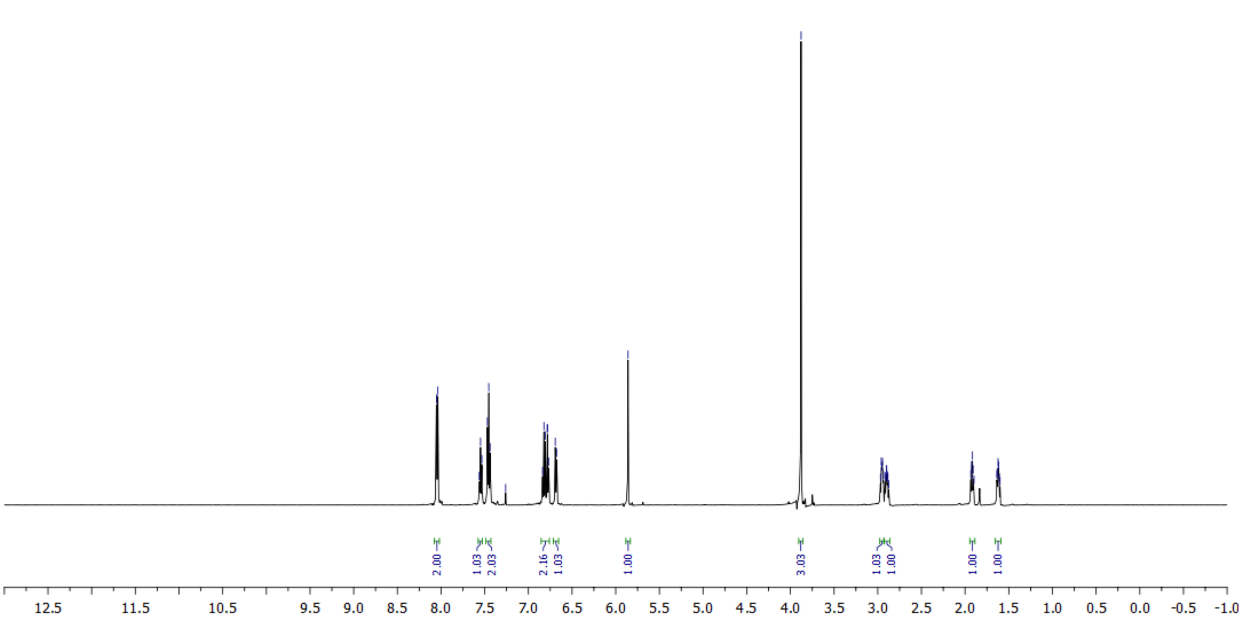

^{13}C NMR (126 MHz, CDCl$_3$)

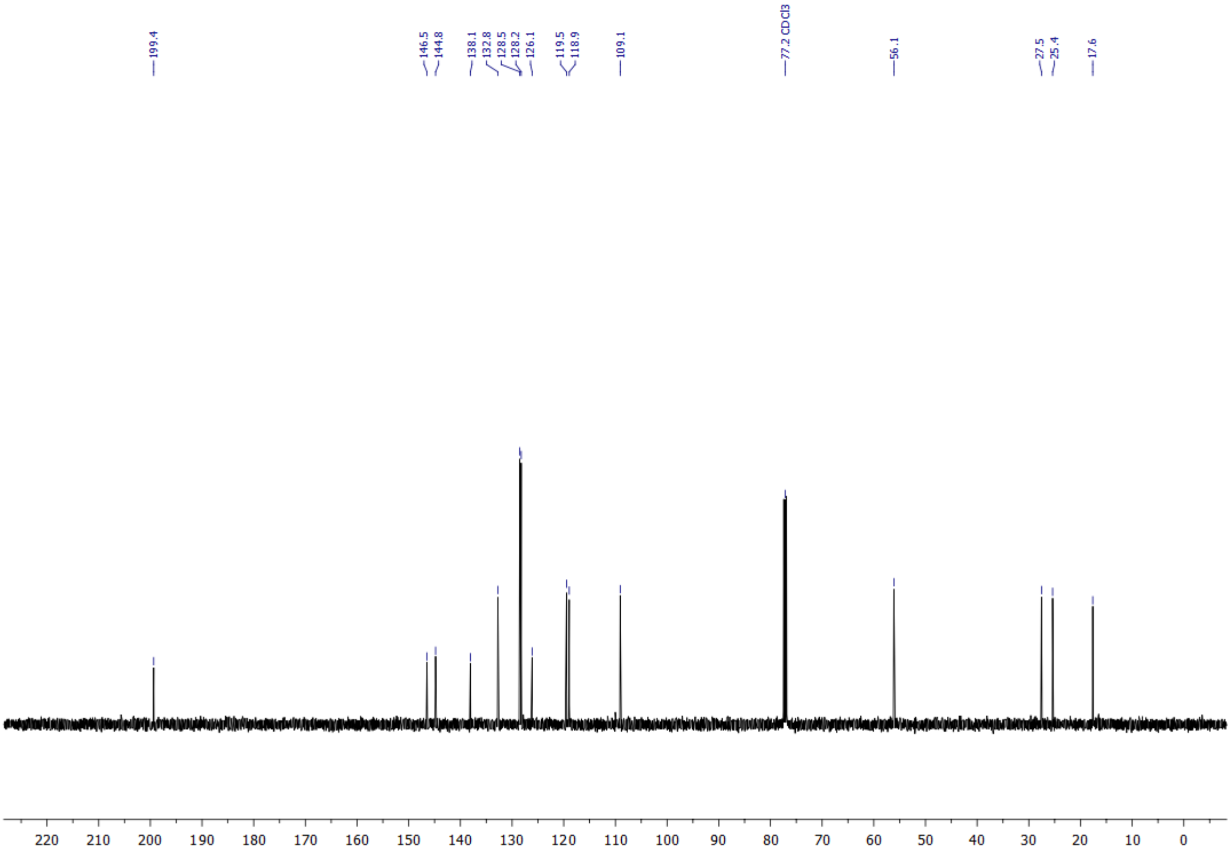

[(1RS,2RS)-2-(3-Ethoxy-2-hydroxyphenyl)cyclopropyl](phenyl)methanone (**2o**)

^{1}H NMR (500 MHz, DMSO-d_6)

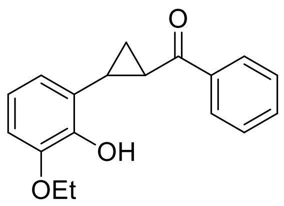

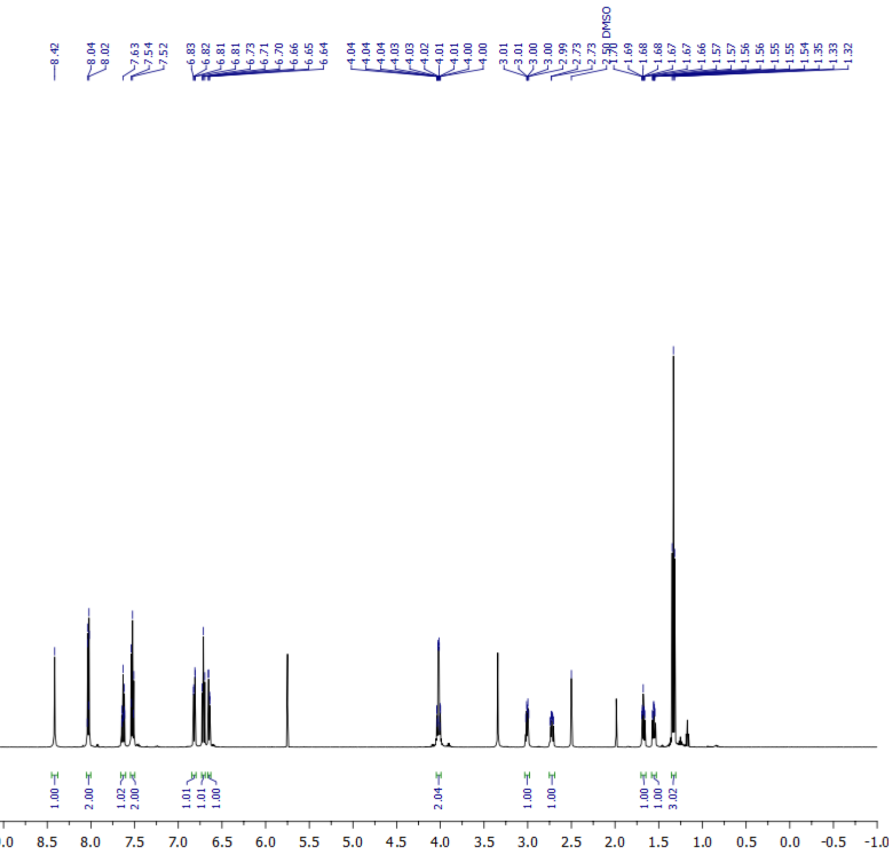

^{13}C NMR (126 MHz, DMSO-d_6)

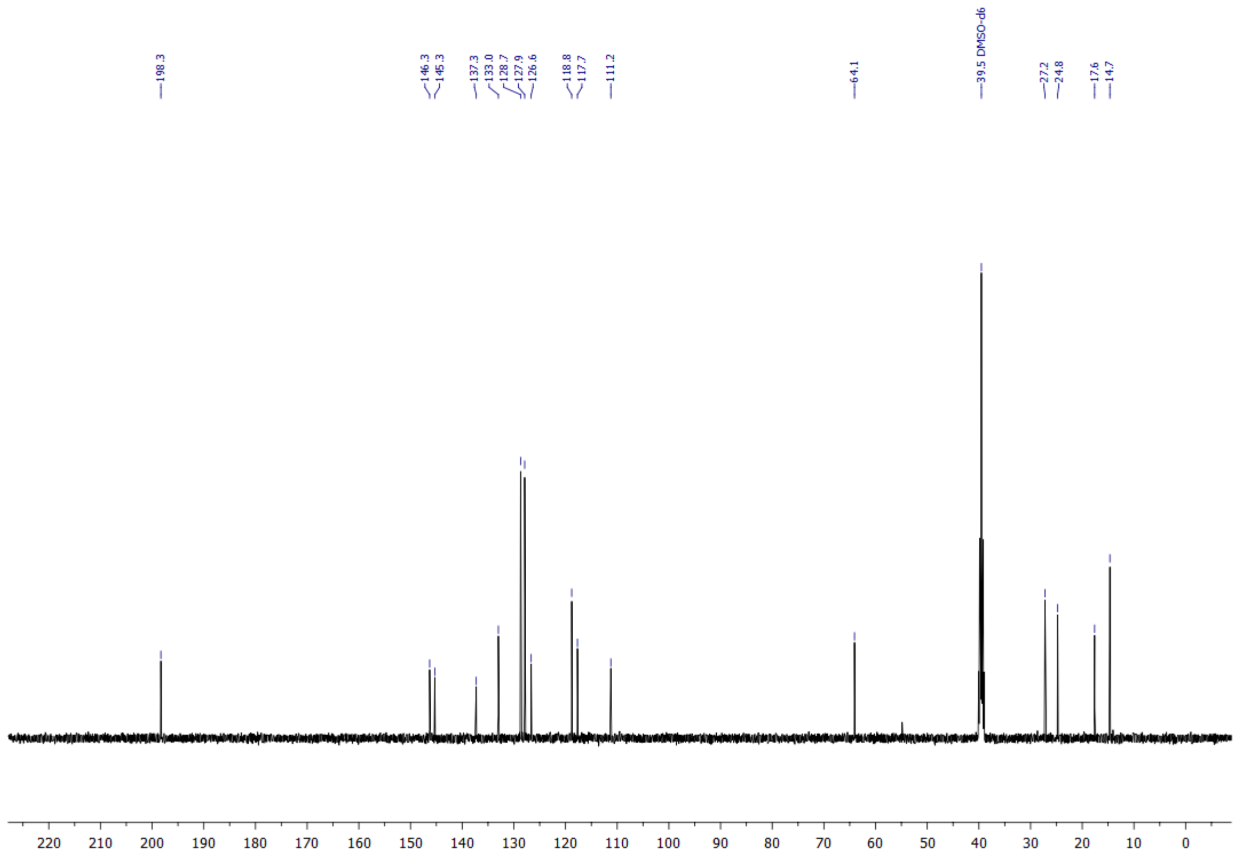

[(1RS,2RS)-2-(3-Ethoxy-2-hydroxyphenyl)cyclopropyl](thiophen-2-yl)methanone (**2p**)

^{1}H NMR (500 MHz, DMSO-d_6)

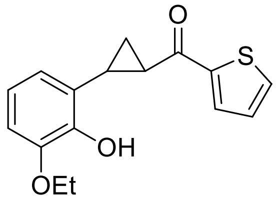

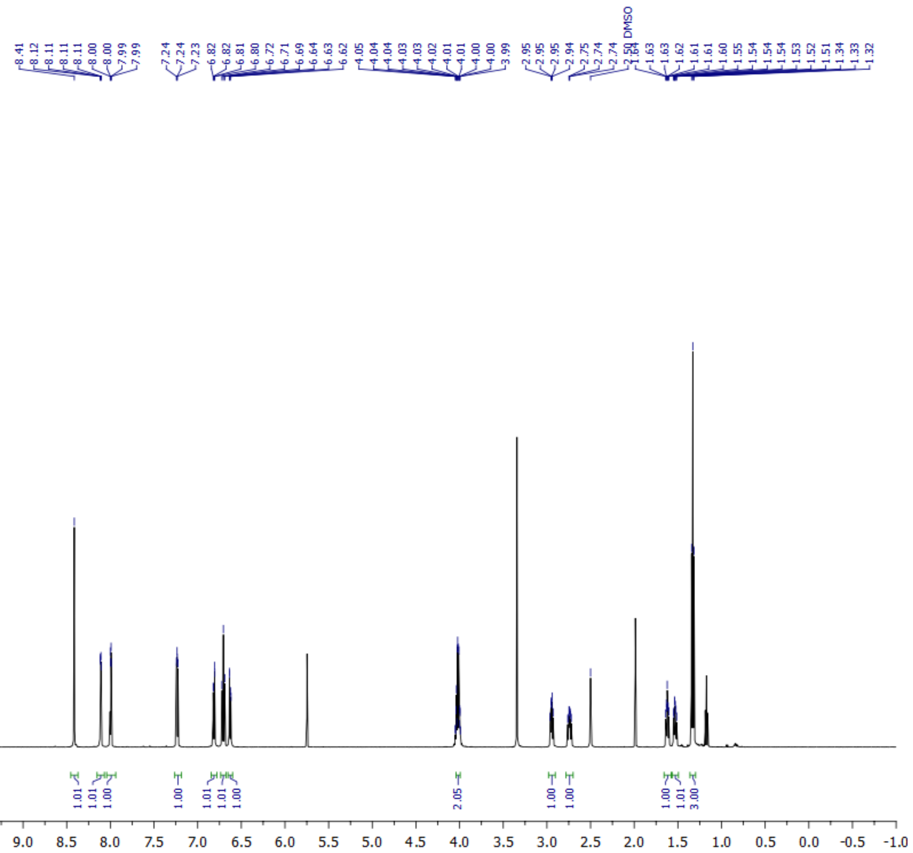

^{13}C NMR (126 MHz, DMSO-d_6)

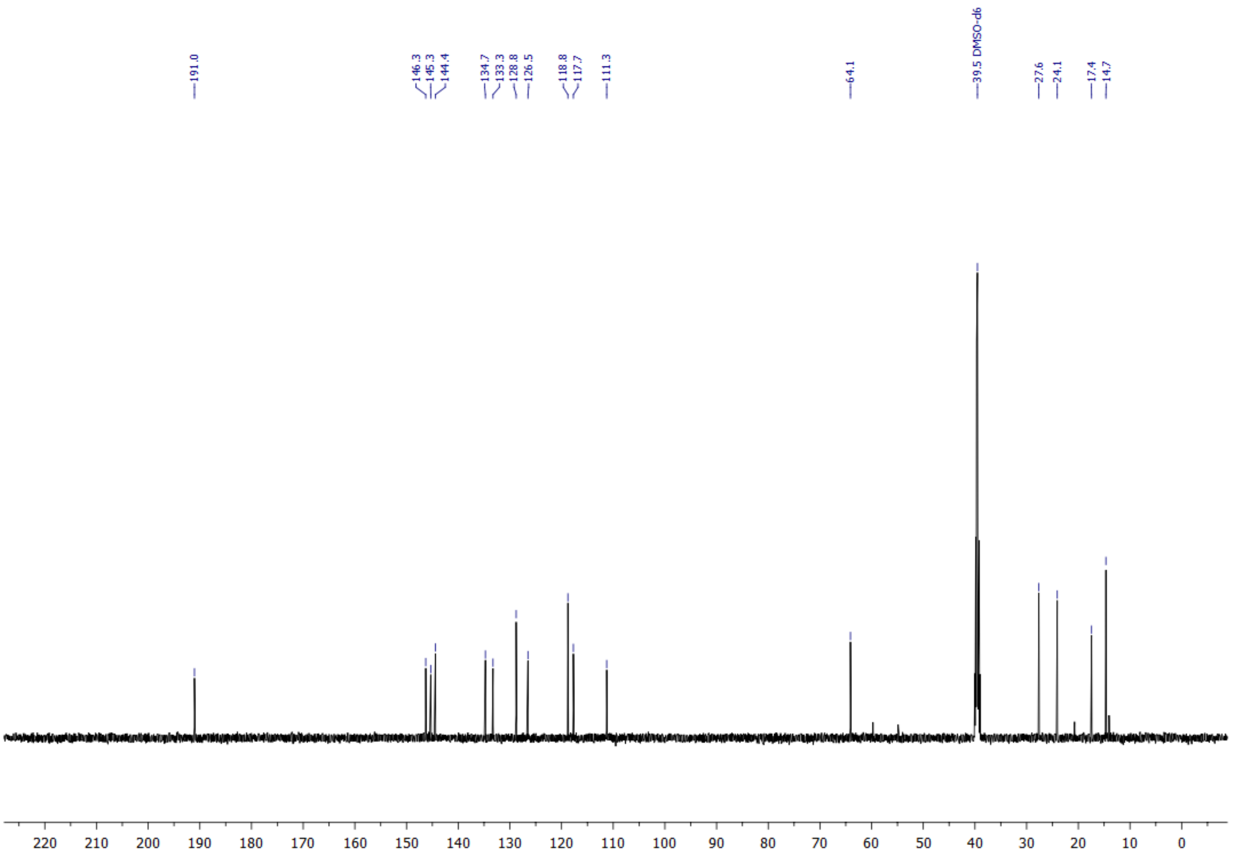

Supplement: Supplementary file 1 [file molecules-25-05748-s001.zip › Supporting information.pdf]
